# Supplementary material for: Mechanism of RACK1-dependent ZAKα activation at stalled and collided ribosomes
Source: Mol Cell. 2026 Jun 18;86(12):2341–2357.e10. doi: 10.1016/j.molcel.2026.04.034 (PMC13293227; doi:10.1016/j.molcel.2026.04.034)
Supplement: Document S1. Figures S1–S7 [file mmc1.pdf]

Molecular Cell, Volume *86*

Supplemental information

Mechanism of RACK1-dependent ZAKα activation

at stalled and collided ribosomes

Anna Constance Vind, José Francisco Martínez, Zhenzhen Wu, Andrii Bugai, Kelly Mordente, Giancarlo Abis, Sébastien Chamois, Sofia Ramalho, Catarina Pechincha, Laura Ryder, Qiuyan Chen, Mads Rasmussen, Xinyao Shi, Dandan He, Jesper Q. Svejstrup, Peter Haahr, David Gatfield, Maria R. Conte, Torben Heick Jensen, Melanie Blasius, and Simon Bekker-Jensen

Vind et al, Figure S1

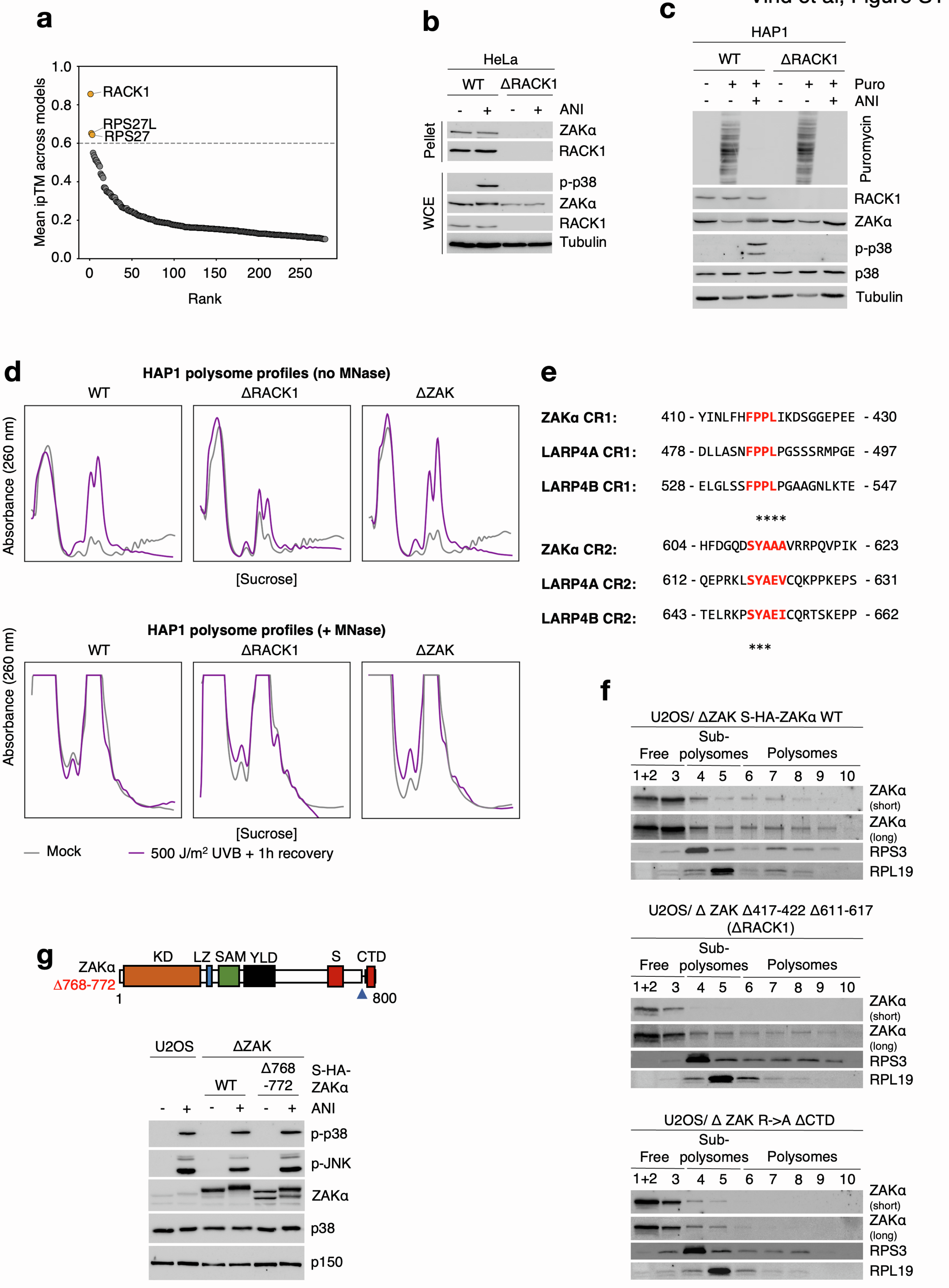

Figure S1.

RACK1 is required for ZAKα-ribosome interaction, related to Figure 1 and Figure 2

a. Alphafold3 (AF3) prediction scores for ZAKα binding to proteins in the Reactome (reactome.org) pathway "Translation", R-HSA-72766. Predictions are ranked according to ipTM score and dashed line indicates our cutoff score of ipTM = 0.6. **b.** Hela WT and ΔRACK1 cells were treated with ani (1 μM - 1 h) and lysates were ultracentrifuged through sucrose cushions. Whole cell extract (WCE) and pelleted material (pellet) containing ribosomes were analyzed by immunoblotting with the indicated antibodies. **c.** HAP1 WT and ΔRACK1 cells were treated with anisomycin (Ani - 0.5 μg/ml, 1 hour). Puromycin (10 μg/ml) was added to the culture 10 min prior to harvest and lysates were analyzed by immunoblotting with the indicated antibodies. **d.** HAP1 WT, ΔRACK1 and ΔZAK cells were irradiated with UVB (500 J/m^2 - 1 hour). Lysates were treated (bottom) or not (top) with MNase to convert polysomes to monosomes. Materials were separated on a linear sucrose gradient and UV absorbance was measured with a fraction collector to indicate RNA (ribosome) content. **e.** Alignment of ZAKα short linear interaction motifs (SLIMs) binding to RACK1 with motifs in LARP4A and LARP4B (CR1 and CR2) previously shown to occupy the same binding sites on RACK1. Note the high identity / similarity between residues that directly contact RACK1. **f.** Polysomal material from U2OS / ΔZAK cells stably rescued with WT and mutated forms of strep-HA-tagged ZAKα were separated by sucrose gradient centrifugation. Proteins in collected fractions were precipitated and analyzed by immunoblotting with the indicated antibodies. **g.** U2OS / ΔZAK cells stably rescued with WT and an RPS27 binding-deficient mutant of strep-HA-tagged ZAKα were treated as in (b) and analyzed by immunoblotting with the indicated antibodies.

Vind et al, Figure S2

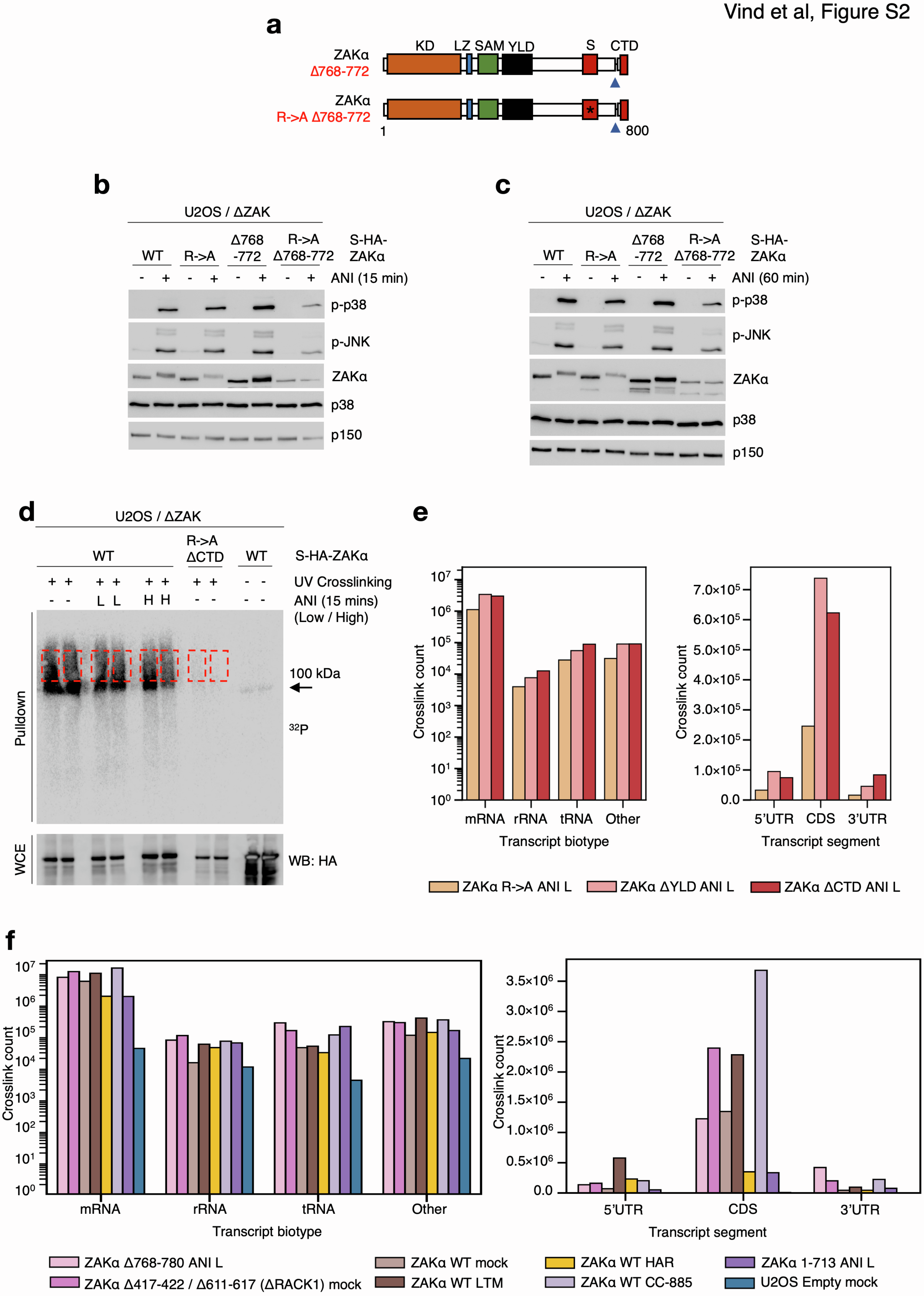

Figure S2.

RPS27 binding is dispensable for ZAKα activation, related to Figure 2

a. Schematics of ZAKα mutants deficient for RPS27 binding (Δ768-772) with and without (R->A) S domain functionality. **b.** U2OS / ΔZAK cells stably rescued with strep-HA-tagged ZAKα mutants from (a) were treated with anisomycin (ani – 1 μM, 15 min). Lysates were analyzed by immunoblotting with the indicated antibodies. **c.** As in (b), except that cells were treated with ani for 60 min. **d.** U2OS / ΔZAK cells stably rescued with WT and ribosome-binding deficient (R->A ΔCTD) forms of strep-HA-tagged ZAKα were treated with ZAK inhibitor (10 μM, 30 min) and anisomycin (ani L – 0.19 μM; Ani H - 76 μM) for 15 min as indicated. Cells were crosslinked by UVC irradiation (150 mJ/cm^2) and lysates were treated with DNase and RNase and subjected to strep purification. RNA 3' ends were ligated to L3-App linkers and radiolabelled with ^{32}P. Material was separated by PAGE and developed by autoradiography. Red boxes indicate the areas of the gel that were excised and processed for library preparation and sequencing. **e.** Left: Total number of ZAKα crosslinks from samples of sequencing batch 2 from Fig. 2f according to RNA category. Right: As in (left), except according to mRNA elements. **f.** As in (e), except for crosslinks from batch 3 from Fig. 2f.

Vind et al, Figure S3

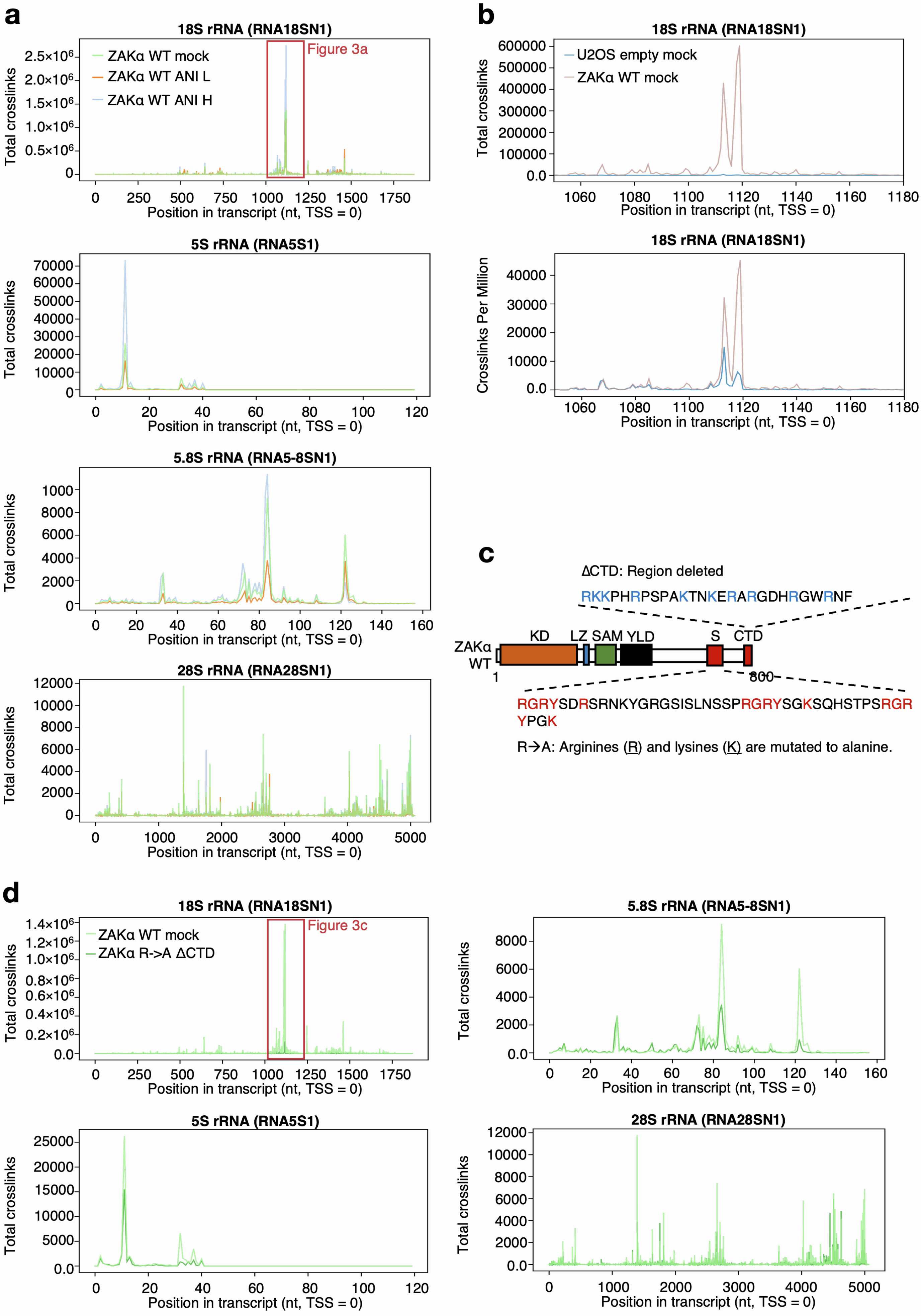

Figure S3.

iCLIP highlights a single prominent rRNA interaction site for ZAKα, related to Figure 3

a. Total number of sequenced ZAKα crosslinks for individual nucleotides across the four rRNAs. Mock treatment, low anisomycin (ani L) and high anisomycin (ani H) (15 min) conditions have been overlaid. **b.** Total (top) and normalized (bottom) 18S rRNA crosslink counts for WT strep-HA-ZAKα compared to empty U2OS cells subjected to strep purification. **c.** Schematic of ZAKα domain composition. The CTD consists of a span of positively charged amino acids (R, K). The S domain contains three peptide repeats of similar sequence. Mutation of Rs and Ks (red) to As in the S domain combined with deletion of the CTD gives rise to the activation- and ribosome binding-deficient mutant of ZAKα (R->A ΔCTD). KD, kinase domain; LZ, leucine zipper; SAM, sterile alpha-motif; YLD, Yeats-like domain; S, sensor domain; CTD, C-terminal domain. **d.** As in (a), except that crosslinks from WT and R->A ΔCTD ZAKα have been overlaid.

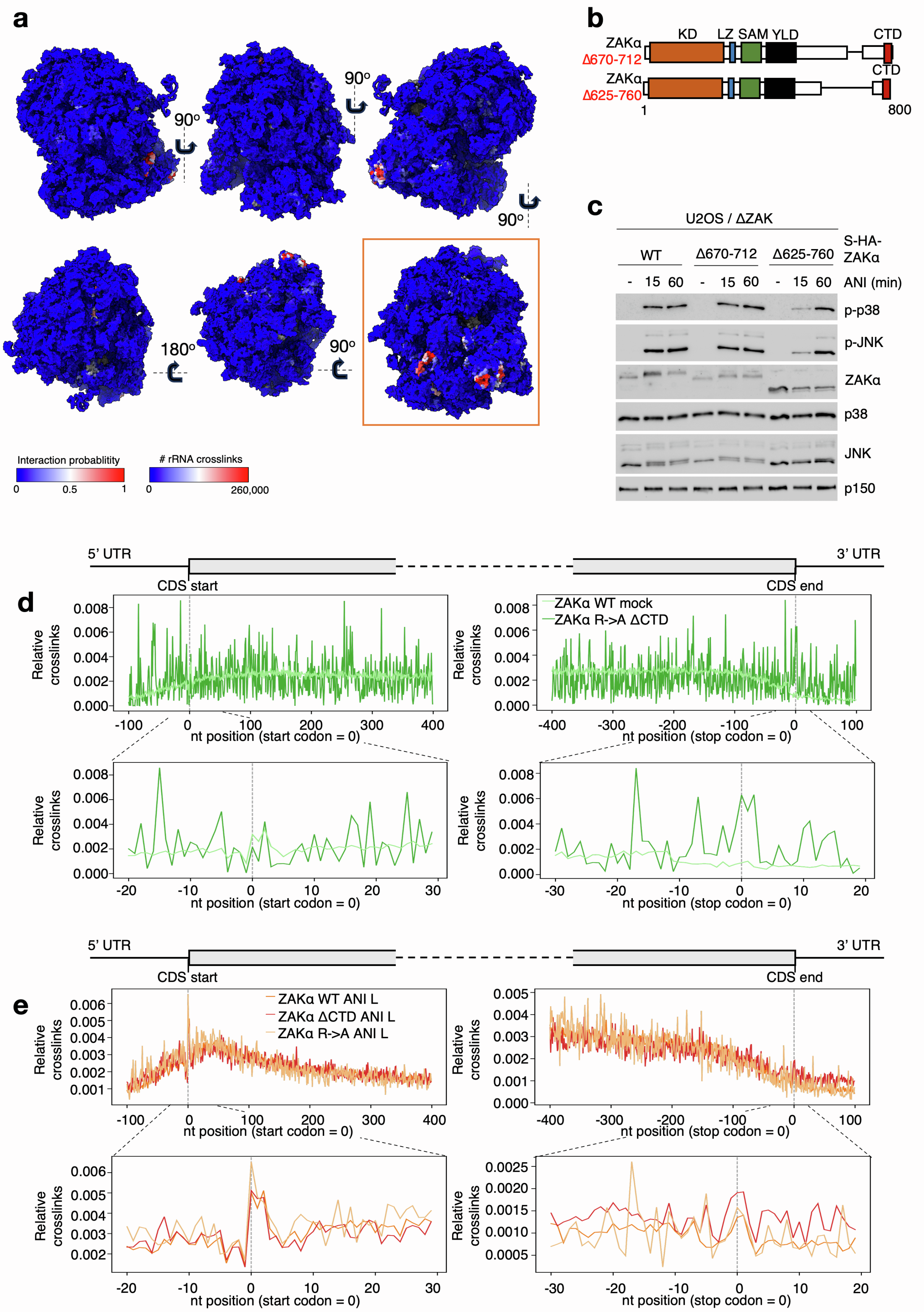
a
90°
90°
90°
180°
90°
Interaction probablitity
0
0.5
1
rRNA crosslinks
0
260,000
b
ZAKα
Δ670-712
KD
LZ
SAM
YLD
CTD
ZAKα
Δ625-760
CTD
1
800
c
U2OS / ΔZAK
WT
Δ670-712
Δ625-760
S-HA-ZAKα
- 15 60 - 15 60 - 15 60
ANI (min)
p-p38
p-JNK
ZAKα
p38
JNK
p150
d
5' UTR
CDS start
CDS end
3' UTR
ZAKα WT mock
ZAKα R->A ΔCTD
Relative crosslinks
nt position (start codon = 0)
nt position (stop codon = 0)
e
ZAKα WT ANI L
ZAKα ΔCTD ANI L
ZAKα R->A ANI L

Figure S4.

Crosslinking of ZAKα S and CTD mutants to mRNA, related to Figure 3 and Figure 4

a. Structure of the human ribosome (PDB 4UG0) painted by per-residue ZAKα interaction probabilities and number of ZAKα crosslinks per rRNA residue. Pictures represent the structure from Fig. 3f viewed from all six sides, highlighting two likely ZAKα interaction sites on the ribosome. **b.** Schematic of ZAKα mutants with shortened linkers (Δ670-712 and Δ625-760). **c.** U2OS / ΔZAK cells stably rescued with strep-HA-tagged ZAKα mutants from (b) were treated with anisomycin (ani – 1 µM) for the indicated times. Lysates were analyzed by immunoblotting with the indicated antibodies. **d.** Analysis of normalized mRNA crosslinks for WT and R->A ΔCTD ZAKα around the start (left) and stop codons (right) shown at low (top) and high (bottom) resolution. **e.** As in (d), except that cells expressing WT, R->A and ΔCTD forms of strep-HA-tagged ZAKα were treated with ani (1 µM, 15 min). nt, nucleotide.

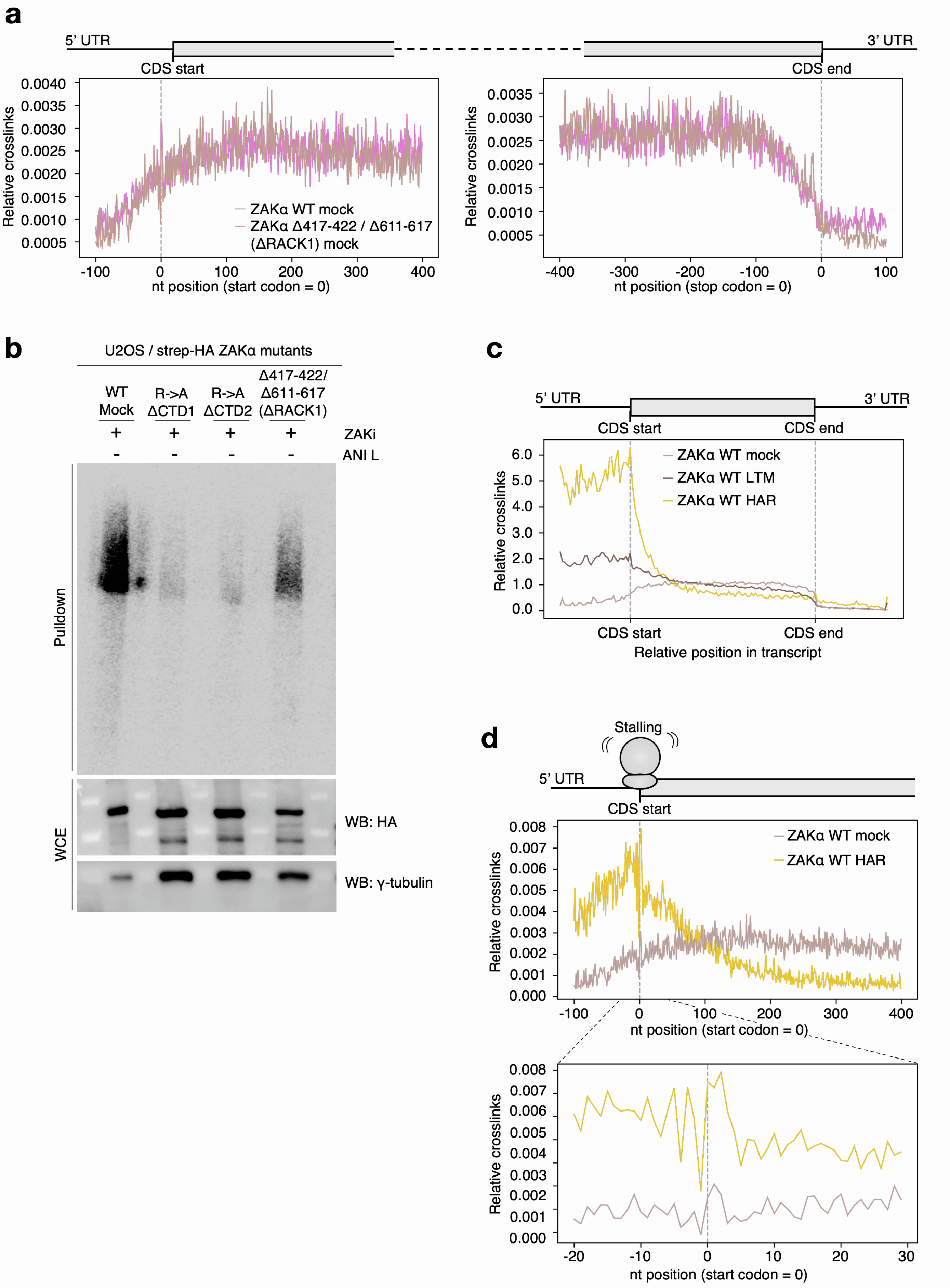
a
5' UTR
3' UTR
CDS start
CDS end
Relative crosslinks
ZAKα WT mock
ZAKα Δ417-422 / Δ611-617 (ΔRACK1) mock
nt position (start codon = 0)
nt position (stop codon = 0)
b
U2OS / strep-HA ZAKα mutants
WT Mock
R->A ΔCTD1
R->A ΔCTD2
Δ417-422/ Δ611-617 (ΔRACK1)
ZAKi
ANI L
Pulldown
WCE
WB: HA
WB: γ-tubulin
c
5' UTR
3' UTR
CDS start
CDS end
Relative crosslinks
ZAKα WT mock
ZAKα WT LTM
ZAKα WT HAR
Relative position in transcript
d
Stalling
5' UTR
CDS start
Relative crosslinks
ZAKα WT mock
ZAKα WT HAR
nt position (start codon = 0)

Figure S5.

mRNA crosslinking of ZAKα relative to position of stalled vs. collided ribosomes, related to Figure 6

a. Analysis of normalized mRNA crosslinks for RACK1 binding-deficient ZAKα (ZAKα Δ417-422 Δ611-617) around the start (left) and stop codons (right). **b.** Comparison of crosslinking efficiency of WT, mRNA binding-deficient (ZAKα R->A ΔCTD) and RACK1 binding-deficient (ZAKα Δ417-422 Δ611-617) mutants of ZAKα. Crosslinked RNA was radiolabelled with ^{32}P. Input materials were analyzed by immunoblotting with the indicated antibodies. **c.** Metagene profiles of total number of crosslinks for mock, LTM-treated (15 μM, 1 h) and HAR-treated (30 μM, 1 h) WT ZAKα along scaled length of spliced mRNAs determined by iCLIP. **d.** Analysis of normalized mRNA crosslinks for ZAKα from cells treated with HAR (30 μM – 1 h) around the start codon shown at low (top) and high (bottom) resolution. nt, nucleotide; LTM, lactimidomycin; HAR, harringtonine.

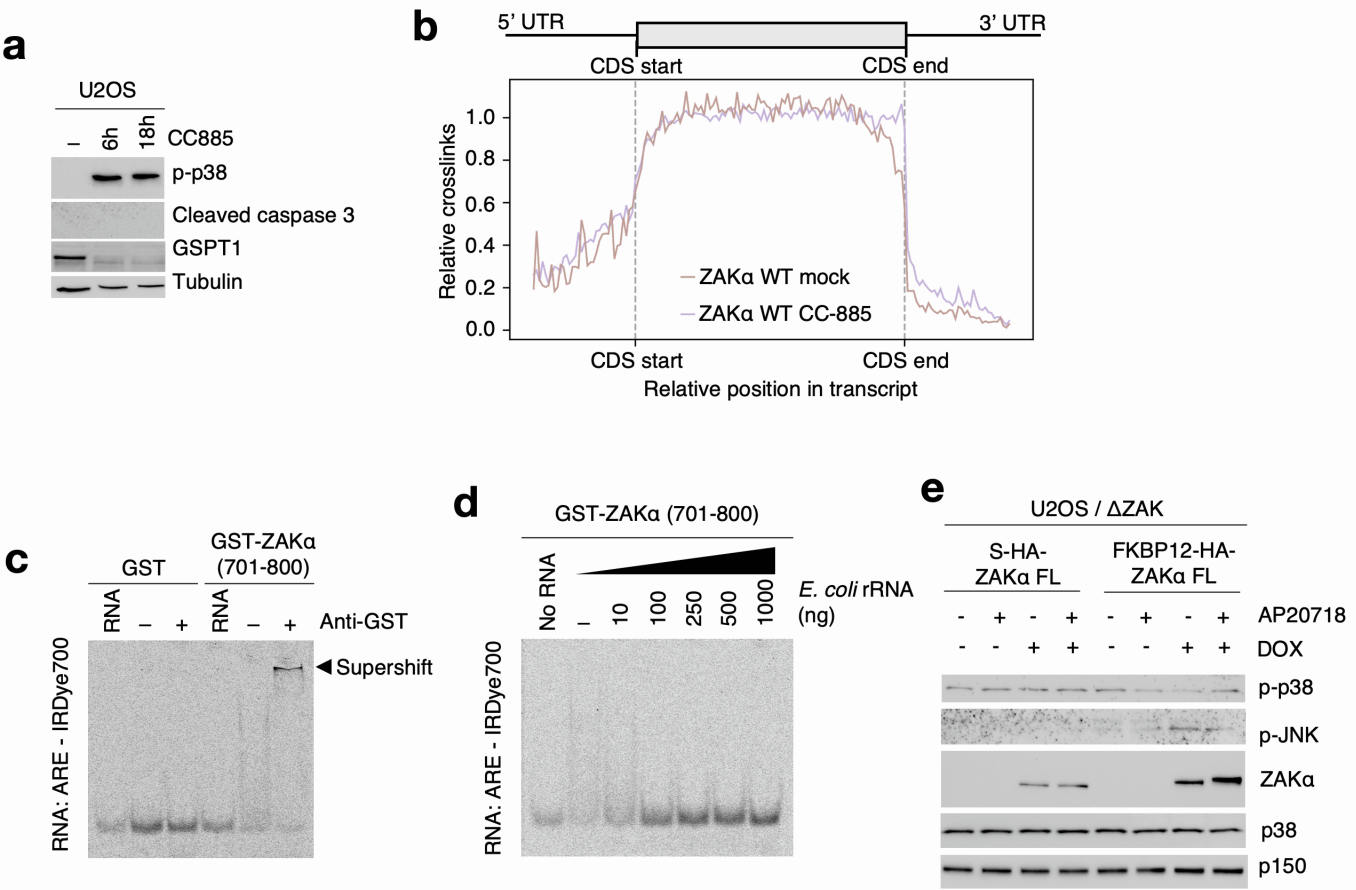
a
U2OS
– 6h 18h CC885
p-p38
Cleaved caspase 3
GSPT1
Tubulin
b
5' UTR
3' UTR
CDS start
CDS end
Relative crosslinks
1.0
0.8
0.6
0.4
0.2
0.0
ZAKα WT mock
ZAKα WT CC-885
Relative position in transcript
c
GST
GST-ZAKα (701-800)
RNA – + RNA – + Anti-GST
Supershift
RNA: ARE - IRDye700
d
GST-ZAKα (701-800)
No RNA – 10 100 250 500 1000
E. coli rRNA (ng)
RNA: ARE - IRDye700
e
U2OS / ΔZAK
S-HA-ZAKα FL
FKBP12-HA-ZAKα FL
AP20718
DOX
p-p38
p-JNK
ZAKα
p38
p150

Figure S6.

Forced degradation of GSPT1 leads to ribosome stalling and collision at stop codons and activation of ZAKα, related to Figure 6 and Figure 7

a. U2OS cells were treated with the GSPT1/ERF3 degrader CC-885 for 6 and 18 h. Lysates were analyzed by immunoblotting with the indicated antibodies. **b.** Metagene profiles of total number of crosslinks for mock and CC-885-treated (6 h) WT ZAKα along scaled length of spliced mRNAs determined by iCLIP. Notice the accumulation of termination-deficient ribosomes around stop codons. **c.** Recombinant GST or a GST-tagged ZAKα fragment (aa 701-800) was incubated with an AU-rich RNA probe labelled with an infrared dye. GST antibody was added to the indicated reactions (resulting in a supershifted band) and complexes were separated by native gel electrophoresis. **d.** GST-ZAKα-RNA complexes from (c) were incubated with unlabeled *E. coli* rRNA (to compete for binding) in the indicated amounts and analyzed as in (c). **e.** U2OS / ΔZAK cells conditionally expressing FKBP12-HA-ZAKα full-length (FL) from Fig. 7a were treated with doxycycline (DOX - overnight) and AP20187 (50 nM, 1 h) as indicated. Lysates were analyzed by immunoblotting with the indicated antibodies.

Vind et al, Figure S7

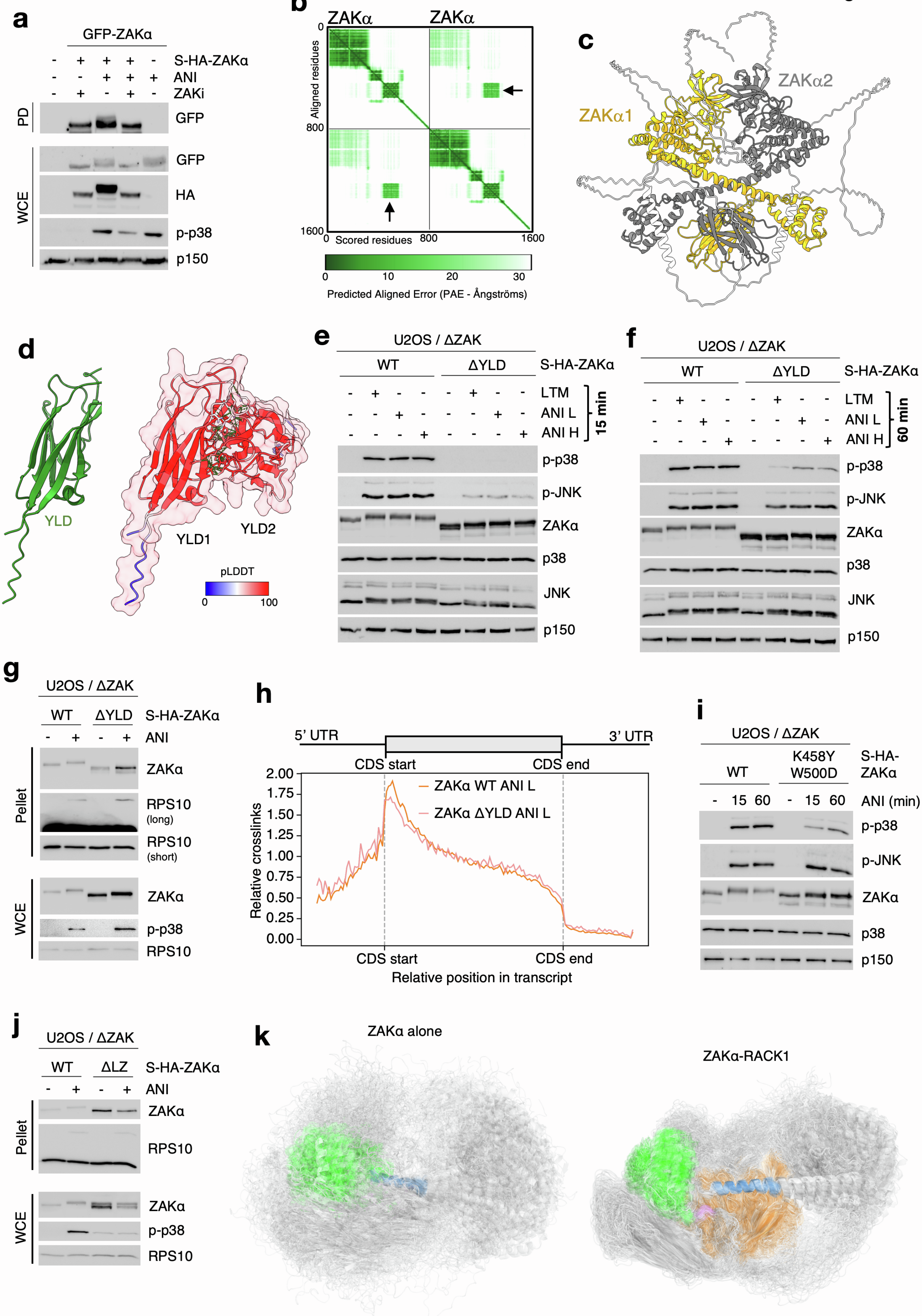

Figure S7.

ZAKα YLD is required for optimal kinase activation, related to Figure 7

a. U2OS cells stably expressing GFP-ZAKα were transfected with Strep-HA-ZAKα and treated with anisomycin (ani - 1 μM) and ZAK inhibitor (ZAKi - 10 μM) for 1 h as indicated. Lysates were subjected to strep purification and pull-down (PD) material and whole cell extract (WCE) were analyzed by immunoblotting with the indicated antibodies. **b.** Predicted aligned error (PAE) matrix plot of AF3-generated prediction of a ZAKα dimer. Values for YLD-YLD binding are indicated with black arrows. **c.** AF3-generated structure from (b). Sequences from start until end of the YLD domain are highlighted by color. **d.** Left: Predicted structure of an isolated ZAKα YLD domain. Right: Predicted structure of a ZAKα YLD dimer. Hydrogen bonds between chains are indicated and chains and interacting side chains are colored according to the predicted local distance difference test (pLDDT) score. **e.** U2OS / ΔZAK cells stably rescued with WT and ΔYLD forms of strep-HA-tagged ZAKα were treated with ribotoxic stress agents ani (L – 0.19 μM; H - 76 μM) or lactimidomycin (LTM – 1 μM) for 15 min and analyzed by immunoblotting with the indicated antibodies. **f.** As in (e), except that cells were treated with ani and LTM for 60 min. **g.** Cells from (e) were treated with ani (1 μM, 1 h) and lysates were ultracentrifuged through sucrose cushions. WCE and pelleted material (pellet) enriched for ribosomes were analyzed by immunoblotting with the indicated antibodies. **h.** Metagene profiles of total number of crosslinks for ani-treated (1 μM, 15 min) ZAKα WT and ΔYLD along scaled length of spliced mRNAs determined by iCLIP. **i.** U2OS / ΔZAK cells stably rescued with WT and YLD point mutated (K458Y W500D) forms of strep-HA-tagged ZAKα were treated with ani (1 μM) for the indicated times. Lysates were analyzed as in (e). **j.** As in (g), except that WT was compared to ΔLZ ZAKα. **k.** Full-length ensembles corresponding to (and colorcoded as in) Fig. 7g.
