# Supplementary material for: Mechanism of RACK1-dependent ZAKα activation at stalled and collided ribosomes
Source: Mol Cell. 2026 Jun 18;86(12):2341–2357.e10. doi: 10.1016/j.molcel.2026.04.034 (PMC13293227; doi:10.1016/j.molcel.2026.04.034)
Supplement: Document S2. Article plus supplemental information [file mmc4.pdf]

Molecular Cell

Article

Mechanism of RACK1-dependent ZAKα activation at stalled and collided ribosomes

Graphical abstract

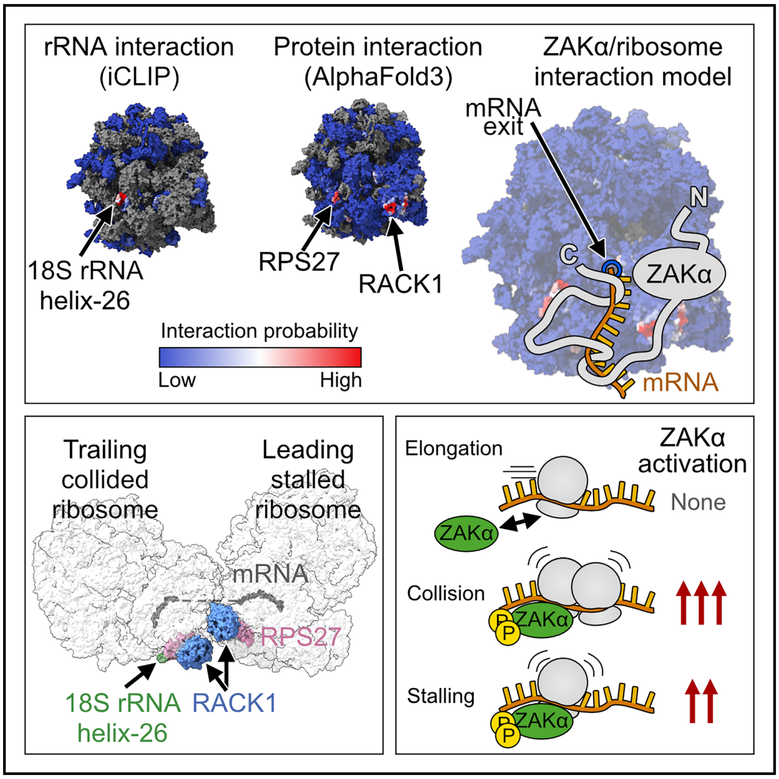

Authors

Anna Constance Vind, José Francisco Martínez, Zhenzhen Wu, ..., Torben Heick Jensen, Melanie Blasius, Simon Bekker-Jensen

Correspondence

sbj@sund.ku.dk

In brief

The ribotoxic stress response is activated upon sensing of impaired translation by the MAP3 kinase ZAKα. Vind et al. demonstrate how ZAKα surveils the translation process and interacts with elongating, stalled, and collided ribosomes. ZAKα activation depends on ribosome-templated sequestration of inhibitory domains, dimerization, and activation-loop autophosphorylation *in trans*.

Highlights

- AlphaFold modeling and RNA crosslinking reveals how ZAKα binds to ribosomes
- ZAKα contacts RACK1, RPS27, 18S rRNA helix-26, and mRNA on the ribosome
- The ribotoxic stress response is activated by ribosome-templated ZAKα dimerization
- The ribotoxic stress response responds to stalled as well as collided ribosomes

Vind et al., 2026, Molecular Cell *86*, 2341–2357
June 18, 2026 © 2026 The Author(s). Published by Elsevier Inc.
https://doi.org/10.1016/j.molcel.2026.04.034

CellPress

Molecular Cell

CellPress
OPEN ACCESS

Article

Mechanism of RACK1-dependent ZAKα activation at stalled and collided ribosomes

Anna Constance Vind,[1,5] José Francisco Martínez,[1,5] Zhenzhen Wu,[1,5] Andrii Bugai,[2] Kelly Mordente,[1] Giancarlo Abis,[3] Sébastien Chamois,[4] Sofia Ramalho,[1] Catarina Pechincha,[1] Laura Ryder,[1] Qiuyan Chen,[1] Mads Rasmussen,[1] Xinyao Shi,[1] Dandan He,[1] Jesper Q. Svejstrup,[1] Peter Haahr,[1] David Gatfield,[4] Maria R. Conte,[3] Torben Heick Jensen,[2] Melanie Blasius,[1] and Simon Bekker-Jensen[1,6,*]

[1]Center for Gene Expression, Department of Cellular and Molecular Medicine, University of Copenhagen, Blegdamsvej 3, 2200 Copenhagen, Denmark
[2]Department of Molecular Biology and Genetics, Aarhus University, Aarhus, Denmark
[3]Randall Centre for Cell and Molecular Biophysics, King's College London, London SE1 1UL, UK
[4]Center for Integrative Genomics, University of Lausanne, 1015 Lausanne, Switzerland
[5]These authors contributed equally
[6]Lead contact
*Correspondence: sbj@sund.ku.dk
https://doi.org/10.1016/j.molcel.2026.04.034

SUMMARY

Despite a growing interest in the ribotoxic stress response (RSR), it remains unknown how the upstream p38- and JNK-activating MAP3 kinase ZAKα senses translational impairment. Combining AlphaFold3 prediction and RNA crosslinking and immunoprecipitation (CLIP), we uncover that ZAKα dynamically monitors the mRNA exit channel of elongating ribosomes. This is accomplished by ZAKα via direct interactions with the ribosomal proteins RACK1 and RPS27 as well as 18S rRNA helix-26. In this conformation, the RNA-binding S (sensing) and C-terminal domain of ZAKα span across the mRNA exit channel. Loss of ribosome processivity and mRNA stasis stabilizes the interaction allowing for kinase activation. Prolonged binding of ZAKα to stalled and collided ribosomes is associated with sequestration of the sterile alpha-motif (SAM) domain on RACK1, which allows for transient ZAKα dimerization, activation loop trans-autophosphorylation, and RSR activation. Our findings highlight how ZAKα senses both stalled and collided ribosomes in human cells through overlapping mechanisms.

INTRODUCTION

The ribotoxic stress response (RSR) denotes a cellular stress response pathway in which the MAP3 kinase ZAKα senses translational aberrations and signals through p38 and JNK kinases.[1] RSR signaling thus holds a potential to impact directly on stress response outcomes such as cell-cycle arrest, programmed cell death, and inflammation. RSR-activating insults can be sustained by a plethora of environmental and endogenous stressors (e.g., UV irradiation[2] and reactive oxygen species[3]), plant and microbial toxins (e.g. ricin and anisomycin[4]), and can even be inflicted on purpose by cellular enzymes (e.g. SLFN11[5,6] and RNase L[7,8]). Recently, the RSR pathway has been shown to be critically important for a range of biologically important phenomena, which includes, but is not limited to, UV-radiation-induced cell death,[9–11] dissemination of *Legionella* bacteria from infected cells,[12] sensitivity of cancer cells to chemotherapy,[6] endoreplication during tumorigenesis,[13] UV-induced skin inflammation,[9] and metabolic regulation in obesity and aging.[3]

Despite the recent surge in RSR research, it remains unknown how the proximal component, the ZAKα kinase, senses translational impairment to mediate these diverse responses. ZAKα has been posited to specifically recognize collided ribosomes,[14] which is a common hallmark of translational stress.[15,16] In this scenario, ZAKα would need to associate with two ribosomes simultaneously or recognize the collision interface to gain specificity in ribotoxic stress sensing. Several well-established examples of signaling from collided ribosomes from eukaryotes[17–19] and bacteria[20–22] lend inspirational support to this notion. ZAKα has also been posited to recognize single stalled ribosomes,[3,23] which is supported by an apparent lack of correlation between the cellular amounts of collided ribosomes and RSR signaling output. Unlike other translation surveillance sensors (GCN2, ZNF598, and RNF10), ZAKα appears to be dynamically associated with elongating ribosomes,[24] which surveil the translation process in a continuous scanning fashion. Given the propensity for rapid and potent RSR activation and the strong imbalance between the number of ZAKα molecules (approx. 5–15,000 per cell) and ribosomes (1–10 mio. per cell), this interaction must

© 2026 The Author(s). Published by Elsevier Inc.
This is an open access article under the CC BY license (http://creativecommons.org/licenses/by/4.0/).

be highly transient and have a high off rate during unperturbed translation. In addition to an N-terminal kinase domain, ZAKα harbors three structured domains; a leucine zipper (LZ), a sterile alpha-motif (SAM), and a YEATS-like domain (YLD) with unknown functions. Ribosome binding has been shown to depend on the C-terminal domain (CTD), which together with the sensor (S) domain is redundantly required for ZAKα activation by all known ribotoxic stress signals.[24] Both the CTDs and the S domains reside in the unstructured C terminus of ZAKα that likely mediates transient interactions with the ribosome.

The current notion in the field is that ZAKα can detect both stalled and collided ribosomes with a preference for the latter. This raises several questions related to the identity of the elusive ribotoxic stress signal(s) that remain unanswered and precludes a proper understanding of the signal-sensor relationship between the ribosome and ZAKα. Here, we provide an explanation for how ZAKα can respond to a plethora of translation-perturbing insults by recognizing a common feature of elongation-impaired and collided ribosomes. ZAKα binds to the ribosome in the vicinity of the mRNA exit channel and further attaches to the exiting mRNA via the S and CTD domains. As the multiple binding sites for ZAKα on mRNA and the ribosome naturally separate from each other during elongation, thus ribosomal processivity inversely correlates with binding time. This binding mode, even when transient, exposes ZAKα in a dimerization-competent and thus activation-prone state. While binding to a single stalled ribosome allows for such activation to take place, the process is exacerbated by ribosome collision, which brings two ZAKα binding platforms into close proximity. We propose a model to explain how the RSR is activated by highly diverse insults and ribosomal conformations with a preference for ribosomal collisions.

RESULTS

RACK1 is critical for ZAKα ribosome interaction and activation

We used AlphaFold3 (AF3)[25] modeling to predict potential interactions between full-length (FL) human ZAKα and all proteins associated with the term "translation" in Reactome[26] (reactome.org, R-HSA-72766). This protein set included all the known ribosomal proteins and translation factors. AF3 predicted with high confidence (ipTM > 0.8) an interaction with the ribosomal protein RACK1 and with somewhat lower confidence (0.8 > ipTM > 0.6) RPS27 and its paralog RPS27L (Figures 1A and S1A). We then extracted the maximal ZAKα interaction probability (from 0 to 1) from AF3 for each amino acid from all ribosomal proteins and painted it onto a structure of the human ribosome (PDB: 4UG0[27]). This analysis exclusively highlighted RACK1 (two surface-exposed patches) and RPS27 (one surface-exposed patch) as potential protein-protein interaction sites for ZAKα on the ribosome, which are in relatively close proximity to each other (Figure 1B). By revisiting a historical genetic screen reporting on ZAKα activation,[28] we noticed that the semi-essential *RACK1* consistently scored as a strong positive regulator while *RPS27* provided a weaker and less significant hit (Figure 1C). We could validate the requirement of RACK1 for anisomycin-induced ZAKα activation in both HAP1 and Hela cells deleted for *RACK1*, and this effect was fully rescued by re-introduction of ectopic RACK1 (Figures 1D, S1B, and S1C). Furthermore, even though RACK1-deficient ribosomes from these cells could both translate and collide (Figures S1C and S1D), they did not co-purify ZAKα, which suggests that RACK1 is indeed critical for ribosome binding of the RSR-activating kinase (Figures 1D and S1B). To further understand the structural basis of this interaction, we examined the AF3-provided dimeric complexes consisting of ZAKα/RACK1 and ZAKα/RPS27. The resulting PAE matrices and inspection of the proposed structures suggested that the interactions are based on three short linear interaction motifs (SLIMs) with a length of 5–6 amino acids. Two of these reside in the unstructured C-terminal 200 amino acids of ZAKα, while the second RACK1-binding motif is located in close proximity to the SAM domain (Figures 1E–1I). Deletion of either of the two SLIMs predicted to bind RACK1 (ZAKα Δ417–422 and ZAKα Δ611–617) rendered ZAKα refractory to activation by anisomycin (Figure 1J), which underscores the critical nature of both interactions. A virtually identical mode of binding to the same two RACK1 pockets via two SLIMs ("CR1" and "CR2") was recently described for the translation-regulating factors LARP4A and LARP4B.[29] The two RACK1-binding SLIMs in ZAKα are almost identical to these sequences (Figure S1E), which suggests a conserved mode of ribosome interaction. We performed ITC (isothermal titration calorimetry) with a peptide spanning one of these SLIMs (similar to CR2 in LARP4A/B) and recombinant RACK1 and observed an *in vitro* binding K_d of around 11 μM for the wild-type (WT) peptide and no binding to a mutated peptide (Figures 2A and 2B). While the ZAKα Δ417–422 mutant retained the ability to co-purify with ribosomes, the deletion of the other RACK1-binding SLIM (ZAKα Δ611–617) completely abrogated this interaction (Figures 2C and S1F), which is consistent with the requirement of the RACK1 protein for the same (Figures 1D and S1B). Finally, we mutated two residues in RACK1 that were predicted by AF3 to destroy the ZAKα 611–617/CR2 binding site (Δα-helix; dAH mutant) (Figure 2D). While transduction of untagged WT RACK1 rescued ZAKα activation in HAP1 ΔRACK1 cells, the dAH mutant failed to do so (Figure 2E). Of note, deletion of the predicted RPS27-binding SLIM alone (ZAKα Δ768–772) did not impair ZAKα activation by anisomycin (Figure S1G), but in combination with the mutation of the S domain (ZAKα R→A Δ768–772), it resulted in a slightly hypomorphic protein (Figures S2A–S2C). Our data shows that RACK1 is a critical hub for both ribosome binding and activation of ZAKα.

iCLIP highlights an rRNA component of the ZAKα-ribosome interaction

For mapping of rRNA bases in close proximity to ZAKα, we employed individual-nucleotide resolution crosslinking and immunoprecipitation (iCLIP) technology[30] (Figures 2F, 2G, and S2D–S2F). We compared rRNA crosslinks of strep-HA-tagged WT ZAKα in the absence or presence of low (0.19 μM; "L") or high (76 μM; "H") anisomycin for 15 min, which are conditions that distinguish collision (L) from stalling/"freezing" (H) of individual ribosomes.[14,31,32] A very prominent double peak centered at U1114 and U1120 of 18S rRNA (Figures 3A and S3A) caught our attention, as this corresponds to 18S helix-26, which is

CellPress
OPEN ACCESS

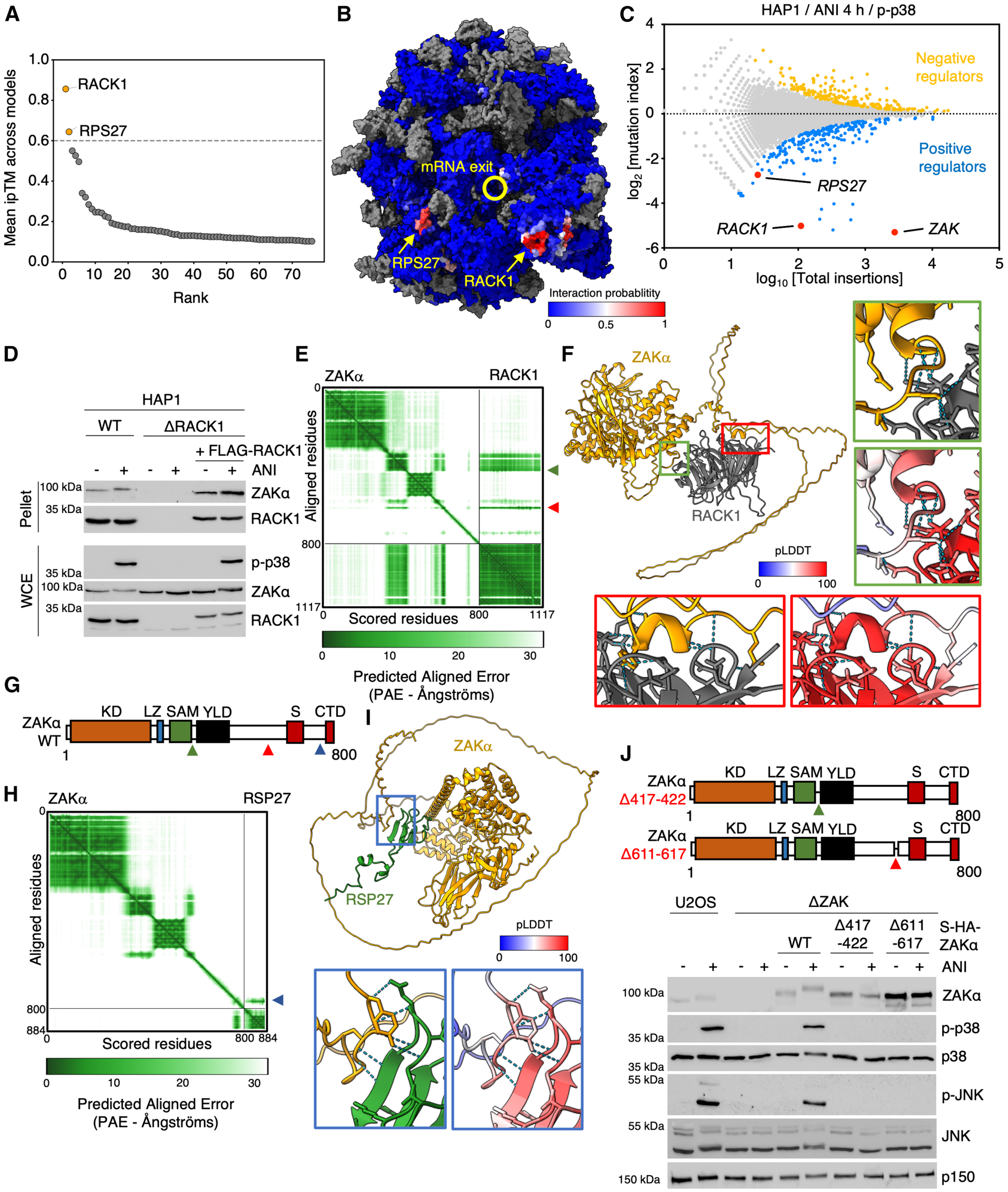

Figure 1. AlphaFold modeling identifies ribosomal protein components binding to ZAKα

(A) AlphaFold3 (AF3) ipTM-ranked scores for ZAKα binding to ribosomal proteins; cutoff ipTM = 0.6.

(B) Per-residue interaction probabilities from (A) mapped onto the ribosome (PDB: 4UG0; 0/blue–1/red).

(C) Gene-trap haploid-cell screen for regulators of anisomycin (ani)-induced p38 activation.

(D) HAP1 WT and ΔRACK1 cells were treated with ani (1 μM, 1 h) and lysates were ultracentrifuged through sucrose cushions. Whole-cell extracts (WCEs) and ribosome pellets were analyzed by immunoblotting.

(E) PAE plot for an AF3 ZAKα-RACK1 model.

(F) AF3 model from (E); interface zooms with H bonds, which are colored by pLDDT.

(G) ZAKα domain map with RACK1-binding (green/red) and RPS27-binding (blue) regions. KD, kinase domain; LZ, leucine zipper; SAM, sterile alpha-motif; YLD, YEATS-like domain; S, sensor domain; CTD, C-terminal domain.

(H) PAE plot for an AF3 ZAKα-RPS27 complex.

(I) AF3-generated structure from (H). Interface zoom as in (F).

(J) U2OS/ΔZAK cells stably rescued with WT and mutated forms of strep-HA-tagged ZAKα were treated as in (D) and analyzed by immunoblotting.

See also Figure S1.

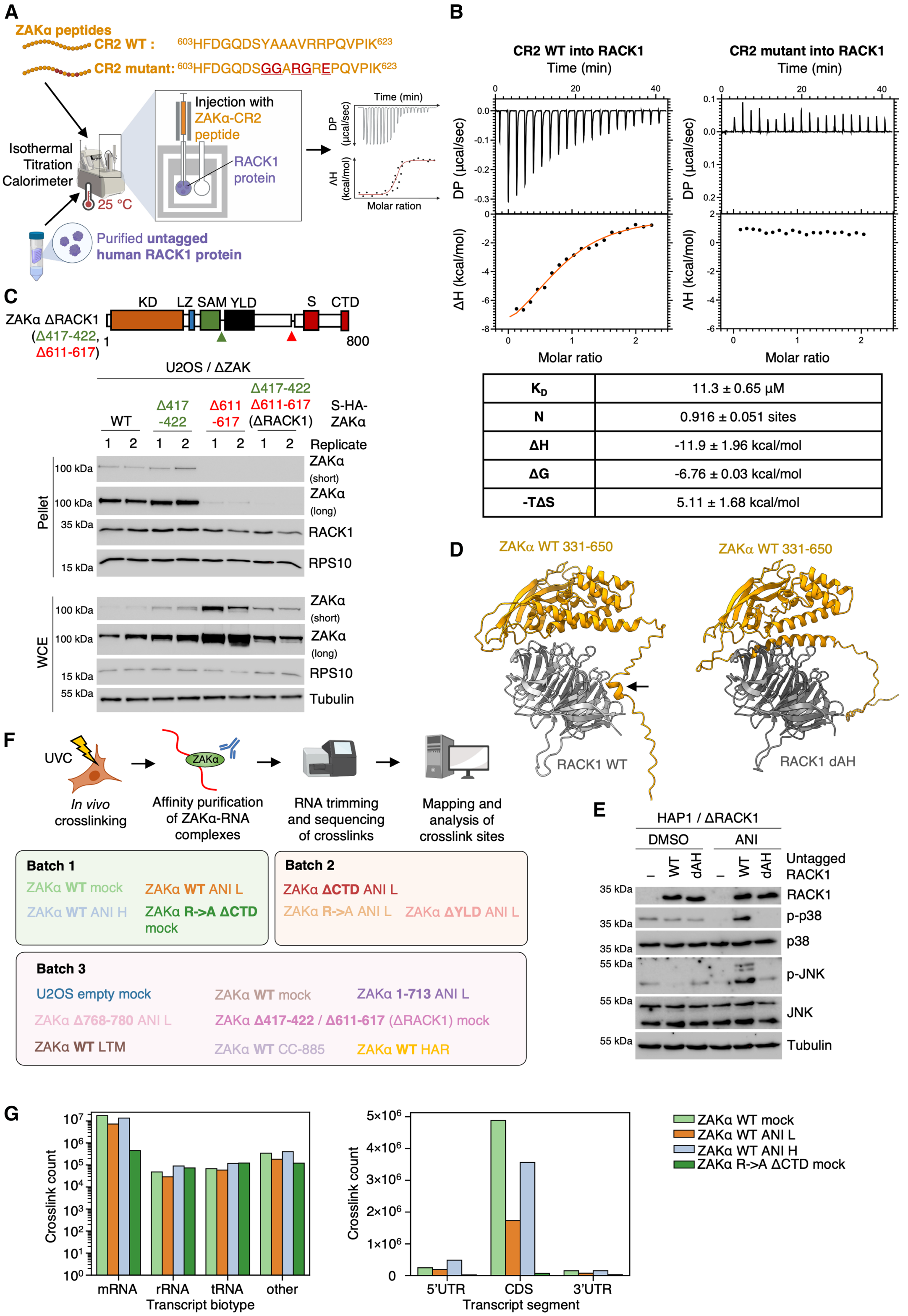

K_D	11.3 ± 0.65 µM
N	0.916 ± 0.051 sites
ΔH	-11.9 ± 1.96 kcal/mol
ΔG	-6.76 ± 0.03 kcal/mol
-TΔS	5.11 ± 1.68 kcal/mol

Figure 2. ZAKα physically interacts with RACK1 for ribosome binding

(A) Isothermal titration calorimetry (ITC) schematic and ZAKα peptides tested for direct RACK1 binding.

(B) ITC of peptides from (A) with recombinant RACK1; raw data/isotherms with K_d and thermodynamics (triplicate).

(legend continued on next page)

placed immediately adjacent to the AF3-proposed ZAKα-RPS27 binding site in the ribosome structure. To control for the validity of this result, we compared WT ZAKα with empty U2OS cells exposed to the same protocol, which only returned very few (background) crosslinks of U1114 and U1120 (Figure S3B). Conversely, the RACK1-binding mutant of ZAKα (Figure 2C) crosslinked to these nucleotides with an efficiency similar to the WT (Figure 3B). We also analyzed our previously described double S and CTD mutant of ZAKα (ZAKα R→A ΔCTD [Figure S3C]) that is refractory to both ribosome binding and activation.[24,33] While this mutant returned as many crosslinks to 28S, 5S, and 5.8S rRNA as WT ZAKα, the amount of crosslinking to 18S rRNA was strongly reduced (Figure S3D). After normalization (crosslinks per million crosslinks), the crosslinking efficiency of the ZAKα mutant, especially to U1120, still remained considerable however (Figure 3C), which demonstrated that its rRNA binding capacity per se is not impaired. Structural alignment of the ribosome with an AF3-generated structure of RPS27 complexed with the last 50 amino acids of ZAKα (± CTD) was consistent with this mutant still being able to contact RPS27 and crosslink to the adjacent nucleotides of 18S helix-26 (Figure 3D). We thus constructed two further mutants, which we expected would abolish 18S helix-26 crosslinking. One had a deletion of the RPS27-binding SLIM and adjacent positively charged amino acids (ZAKα Δ768-780), while the other one had a deletion of the entire region downstream of the S domain (ZAKα 1–713). Rewardingly, neither of these mutants crosslinked considerably to 18S rRNA (Figure 3E), which validated our observation that RPS27 and helix-26 are jointly contacted by a ZAKα SLIM and its downstream amino acids. Combining AF3 modeling and iCLIP analysis by simultaneously color coding the ribosomal surface for AF3-based interaction probabilities and iCLIP-based crosslink counts highlighted how ZAKα engages two sites on either side of the ribosomal mRNA exit channel (Figure 3F). Inspection of this combined model of ribosome-ZAKα interaction from all angles did not point to any alternative interpretation of our data (Figure S4A). Importantly, the overall iCLIP profile was largely unchanged upon addition of either concentration of anisomycin (Figures 3A and S3A), which suggested that interaction with this ribosomal surface is equally relevant for the scanning and sensing modes of ZAKα binding. We investigated the importance of this dual interaction mode by generating and testing several deletion mutants of ZAKα. First, we shortened the linker between the second RACK1-binding SLIM and the RPS27-binding SLIM to an extent where ZAKα cannot simultaneously occupy both binding sites (ZAKα Δ625–712), and second, we enlisted the above mutant that was deficient for both the CTD and binding to RPS27 as well as 18S helix-26 (ZAKα 1–713). Surprisingly, both of these mutants were quite capable of activation when assayed 1 h after anisomycin treatment and only slight hypomorphs when assayed at a shorter time (15 min) after exposure to anisomycin (Figures 3G, S4B, and S4C). These results suggest that while the RACK1-binding SLIMs and one of the S and CTD domains are essential for activation of ZAKα after anisomycin treatment, the composite RPS27-18S helix-26 binding site is relatively unimportant after this specific translational insult.

ZAKα S and CTD domains associate with mRNA to achieve sufficient affinity for ribosome interaction

Our iCLIP experiments also returned abundant ZAKα mRNA crosslinks (Figures 2G, S2E, and S2F). Metagene profiles of total and normalized counts showed enrichment in the 5′ UTR and coding sequences (Figures 4A and 4B). In contrast to WT ZAKα, the ribosome binding-deficient ZAKα R/K→A ΔCTD mutant yielded almost no mRNA crosslinks (Figures 4A and 2G), which indicated that mRNA binding correlates with ribosome interaction. Although both single S and CTD mutants were refractory for ribosome binding during unperturbed translation, each interacted robustly with ribosomes in anisomycin-treated cells and supported RSR signaling, while the double mutant was refractory to both (Figure 4C). Analyzing normalized crosslinks along mRNA segments, the double mutant produced only a signal of noise (Figure S4D). Each single mutant, however, resembled the mRNA crosslinking profile observed for WT ZAKα (Figure S4E). The RACK1-binding mutant (ZAKα Δ417–422 Δ611–617) also crosslinked mRNA (Figures S2F, S5A, and S5B) despite the lack of biochemically tractable ribosome binding (Figure 2C) and anisomycin-induced activation (Figure 1J). Our results explain the curious redundancy of the S and CTD domains,[9,24] in that they both contact mRNA to mediate ribosome binding and ribotoxic stress sensing.

The normalized metagene profile of WT ZAKα across the mRNA highlighted that the bulk of binding occurred in the middle of the coding sequence (CDS) and in the 5′ UTR but not in the 3′ UTR (Figure 4B). This profile changed dramatically with anisomycin treatment with a bias toward the 5′ UTR and the upstream parts of the CDS and with lower occupancy around the stop codon (Figure 4B). To understand this shift in ZAKα mRNA occupancy, we performed ribosome profiling of U2OS cells treated for 15 min with low (L) and high (H) anisomycin. Plotting footprint densities on both a metagene plot and aligned relative to start and stop codons, we unexpectedly observed that anisomycin treatment primarily inhibits ribosomes that are localized toward the beginning of the CDS (Figures 4D and 4E). Thus, our iCLIP results and ribosome profiling data in aggregate indicate that ZAKα follows ribosomes where they are localized on mRNA. The

(C) Lysates from U2OS/ΔZAK cells stably rescued with WT and mutated forms of strep-HA-tagged ZAKα were ultracentrifuged through sucrose cushions. WCEs and ribosome pellets were analyzed by immunoblotting.
(D) AlphaFold3 (AF3) prediction of complexes of a ZAKα fragment spanning amino acids (aa) 331–650 with WT RACK1 (left) and mutated RACK1 (Y246E/L261R; "dAH") (right).
(E) HAP1 ΔRACK1 cells were virally transduced to express WT or dAH RACK1 and treated with ani (1 μM, 1 h). Lysates were analyzed by immunoblotting.
(F) iCLIP schematic and sample overview (three sequencing batches).
(G) (Left) Total number of ZAKα crosslinks from sequencing batch 1 according to RNA category (gene biotype). (Right) As in (left), except according to mRNA elements.
See also Figures S1 and S2.

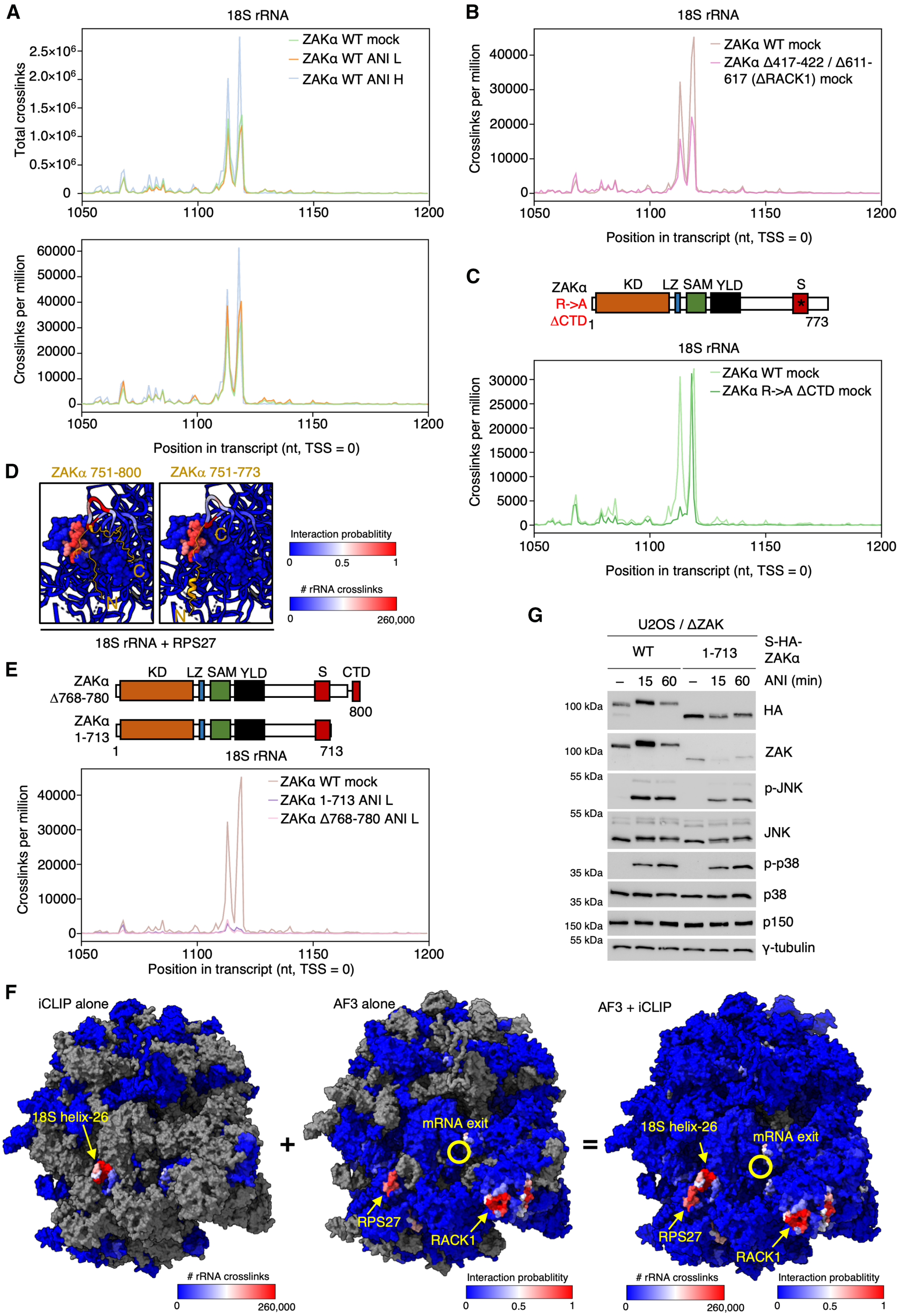

Figure 3. iCLIP reveals an RPS27-proximal rRNA binding site for ZAKα

(A) WT ZAKα crosslinks and library-size-normalized rRNA crosslinks across an 18S rRNA region, overlaid for mock, ani L (0.19 μM), and ani H (76 μM) (15 min).

(B) As in (A), except that normalized WT ZAKα crosslinks were compared with normalized crosslinks for the RACK1-binding mutant (ZAKα Δ417–422 Δ611–617).

(legend continued on next page)

profiles (both iCLIP and ribosome profiling) were highly similar between low ("collision stress") and high anisomycin (ribosome freezing conditions)-treated samples (Figures 4B and 4D), which indicated that ZAKα does not bind mRNA or ribosomes differentially between these two conditions. Our map of ribosomal-binding surfaces indicated that ZAKα surveils the mRNA exit channel of the ribosome (Figure 3F). A closer inspection of mRNA crosslink sites around the stop codon supported this hypothesis, which highlighted a decrease in crosslinks in all conditions at around 12 nt upstream of the stop codon (Figure 4F, right). This gap is reminiscent of a ribosome-protected mRNA fragment originating from a ribosome occupying the last codon of the open reading frame (ORF).[34] A similar inspection of the start codon-proximal region revealed the presence of crosslinks toward the end of the 5′ UTR, which arise preferentially from anisomycin-treated samples and likely represent binding to ribosomes that have been arrested at or closely after the start codon (Figure 4F, left).

ZAKα is activated by stalled *and* collided ribosomes

We treated U2OS cells with L and H concentrations of anisomycin and performed sucrose-gradient centrifugation of MNase-digested polysomal material. In the L condition, we observed clear peaks corresponding to widespread ribosome collision, and these signals were completely gone in the samples from cells treated with the H concentration (Figure 5A). These conditions are identical to those we used for iCLIP and ribosome profiling (Figure 4). We subsequently treated U2OS cells that had the *ZAK* gene deleted (U2OS/ΔZAK), or not (WT), with increasing concentrations of anisomycin. At saturating conditions (100 μg/mL; even higher than H), 15 min of anisomycin treatment was associated with ZAKα-dependent p38 and JNK activation (Figure 5B). Albeit this activation was somewhat less detectable than after lower doses of anisomycin, it was still clearly detectable. When we extended the treatment period to 1 h, no difference in RSR signaling could be observed across the different anisomycin concentrations (Figure 5C). Based on these results, previously published evidence,[3,23] and the fact that lactimidomycin (LTM) and harringtonine (HAR), which inhibit only initiating 80S ribosomes activate the RSR (Figures 5D and 5E), we emphasize that a single stalled ribosome is sufficient to activate ZAKα and RSR signaling in U2OS. Revisiting this conclusion among a panel of commonly used cell lines, we noticed that there are striking differences in the activation potential of ZAKα with respect to treatment and cell line background. Compared with L anisomycin, stalling-inducing H anisomycin treatment was associated with clearly lower ZAKα activation in HAP1 cells and barely detectable activation in HEK293T cells (Figures 5E–5G). HAR treatment also differentially activated ZAKα across these cell lines. While activation was clearly visible in U2OS (Figure 5E) as we previously reported,[24] it was non-existent in HAP1 and HEK293T cells (Figures 5F and 5G). It was recently demonstrated that knockout of GCN1 leads to HAR-induced ZAKα activation in HEK293T cells.[35] This happened in the absence of any detectable ribosome collision and was hypothesized to be the result of impaired activation of the integrated stress response kinase GCN2 and resulting collisions between initiating/scanning 40S subunits and start codon-stalled 80S ribosomes.[35] Here, using HAP1 cells, we could perfectly validate that GCN1 is a powerful suppressor of HAR-induced ZAKα activation in some cell lines (Figure 5h). This is, however, not related to ISR signaling and continued translation initiation, as ΔGCN2 cells did not phenocopy this effect (Figure 5H). Nor is it related to ribosome collision, as HAR treatment of neither WT nor ΔGCN1 HAP1 cells gave rise to MNase-resistant ribosome peaks other than the expected monosome peak (Figures 5I and 5J). We do not, at present, understand the underlying reasons for these cell line differences with regards to ZAKα activation and its repression by GCN1. Disparate conclusions regarding ribosome stalling, collision, and ZAKα activation in the literature could stem from the choice of cell line used for interrogation.

Impaired displacement from the ribosome leads to ZAKα activation

iCLIP of WT ZAKα from cells treated with a high dose of LTM (15 μM) for 60 min (Figures 2F and S2F) returned a large number of mRNA crosslinks upstream of the start codon (Figure S5C). Inspection of the normalized metagene profiles for LTM-treated and unperturbed cells around the start codon revealed that this peak had its zenith at approx. 10 nt upstream of the start codon and did not extend into the CDS (Figure 6A). This paucity of crosslinks in the 5′ UTR corresponds to the length of an mRNA fragment that is protected by a single ribosome stalled at the initiation codon. Inspection of individual gene tracks highlighted many clear examples of ZAKα crosslinking exclusively to the region just upstream of the start codon in LTM-treated cells (Figure 6B). Having demonstrated that the initiating 80S inhibitor HAR also activates ZAKα in WT U2OS cells (Figure 5E), we also performed iCLIP of WT ZAKα from cells treated with this single ribosome-stalling agent (30 μM, 1 h) (Figures 2F and S2F). Analysis of

(C) (Top) Schematic of a ribosome-binding and activation-deficient mutant of ZAKα (R→A ΔCTD). (Bottom) As in (A), except that normalized crosslinks for ZAKα WT vs. R→A ΔCTD were analyzed.

(D) Structural overlay of an experimental structure of the human ribosome (PDB: 4UG0) with AF3-predicted complexes of RPS27 and the C-terminal 50 aa of ZAKα with (left) or without (right) the CTD included. RPS27 in 4UG0 (other ribosomal proteins are hidden) is colored according to interaction probability (protein, AF3 prediction) and total number of WT ZAKα crosslinks (rRNA).

(E) As in (A), except that normalized WT ZAKα crosslinks were compared with normalized crosslinks for two 18S rRNA helix-26 binding mutants (ZAKα Δ768–780 and ZAKα 1–713).

(F) 4UG0 painted by total number of ZAKα crosslinks per rRNA residue (left), per-residue ZAKα interaction probabilities from Figure 1B (middle), and a combination of the two (right), which highlights two ribosomal-binding patches for ZAKα.

(G) U2OS/ΔZAK cells stably rescued with WT and the 1–713 deletion mutant of strep-HA-tagged ZAKα were treated with ani (1 μM, 1 h). Lysates were analyzed by immunoblotting.

See also Figures S3 and S4.

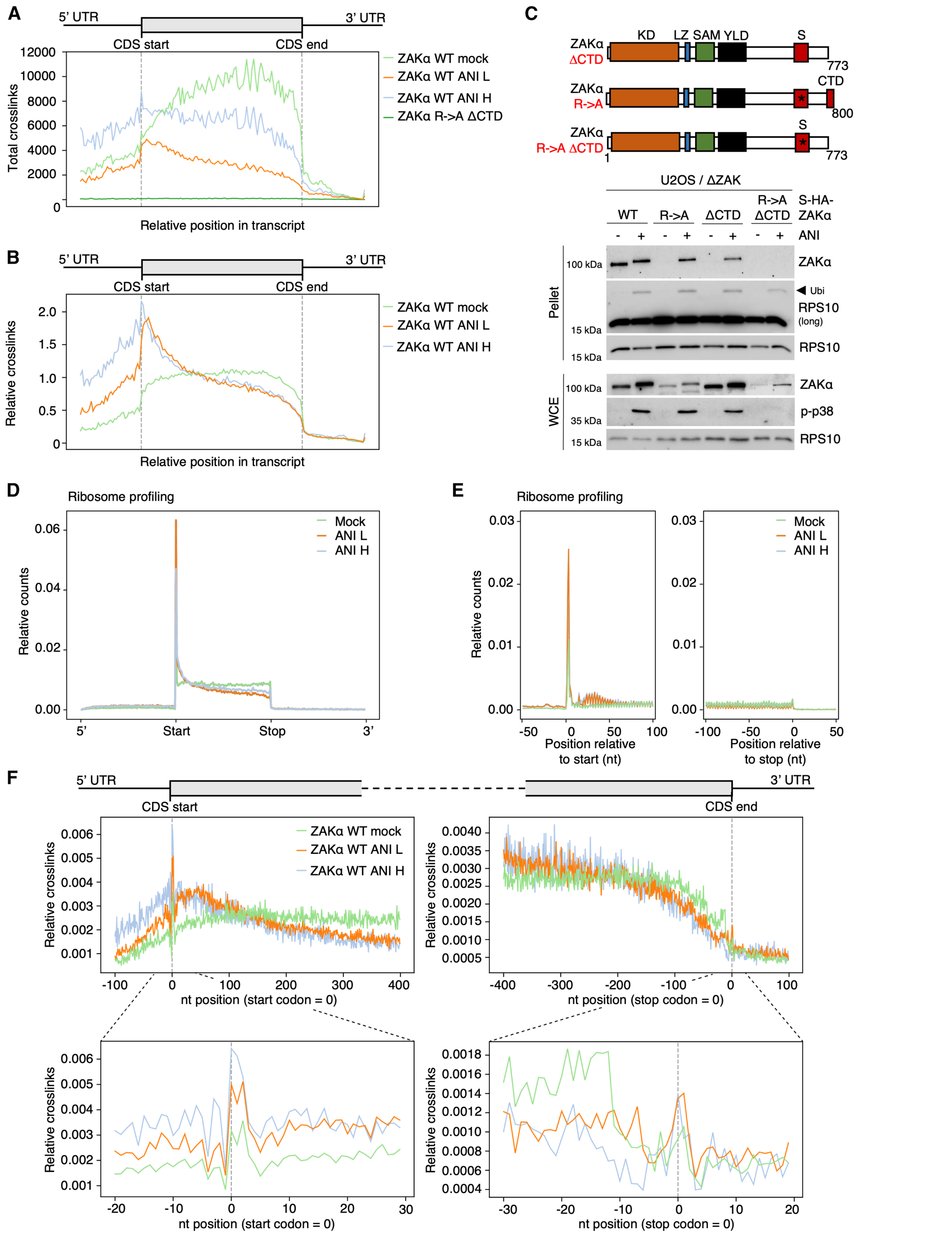

Figure 4. S and CTD domains are required for mRNA crosslinking and critical for ZAKα-ribosome interaction

(A) Metagene profiles of total number of crosslinks for ZAKα WT and R→A ΔCTD along scaled length of spliced mRNAs determined by iCLIP. Cells were treated with ani L, 0.19 μM or ani H, 76 μM (15 min).

(legend continued on next page)

CellPress
OPEN ACCESS

mRNA crosslinks similar to those above essentially replicated the result for LTM (Figures S5C and S5D), albeit with an even more pronounced crosslinking of ZAKα in the 5′ UTR. We then went on to perform similar analyses of ZAKα mRNA crosslinking in the context of localized ribosome collisions. To this end, we employed the GSPT1/ERF3 degrader CC-885,[36] which should lead to the accumulation of dissociation-impaired ribosomes at stop codons. In U2OS cells, treatment of CC-885 for 6 h led to a marked decrease in GSPT1 levels, strong ZAKα activation (Figure S6A), and increased crosslinking of ZAKα to stop codon-proximal mRNA (Figure S6B). Also, in these metagene profiles, we could observe a strong crosslinking peak upstream of the stop codon at a distance (12 nt) that corresponded to the ribosome-protected fragment (Figure 6C). However, CC-885 treatment also gave rise to a second peak starting at −44 nt, which is consistent with the spacing of a full ribosome-protected fragment (28–30 nt[34]) from the first peak (Figure 6C). We interpret these peaks as evidence for ZAKα crosslinking to mRNA exiting a stalled and a collided ribosome, respectively.

These results, along with our AF3-modeling and other iCLIP data, allowed us to formulate a model for ribosome binding by ZAKα. Here, ZAKα associates transiently with RACK1, RPS27, 18S helix-26, and mRNA protruding from the mRNA exit channel. On elongating ribosomes, movement of the mRNA component of this composite binding reaction will destabilize the interaction, which effectively ejects ZAKα from the ribosome shortly upon arrival (Figure 6D). We further propose that ZAKα, from this position, can sense the compromised processivity of stalled 80S ribosomes when they are loaded onto mRNA. A key component of this sensing model is the direct contact between ZAKα S and CTD domains with mRNA. We have previously demonstrated that a ZAKα fragment encompassing a part of the S domain as well as the whole CTD domain (amino acids 701–800) binds directly to structured RNA.[24] By electrophoretic mobility shift assay (EMSA), we could show that this is also true for a seemingly unstructured RNA. A random AU-rich RNA probe bound to ZAKα 701–800 was indicated by an antibody-based mobility supershift (Figure S6C) and out-competition by unlabeled RNA (Figure S6D). By interrogating a structure of a disome (PDB: 7QVP[18]), it appeared unlikely that ZAKα can simultaneously connect to RACK1 and RPS27-18S helix-26 of the leading (stalled) ribosome while also gaining access to the very short stretch of mRNA bases that are partially buried at the collision interface (Figure 6E). It is, however, entirely possible for ZAKα to simultaneously employ the same binding and sensing mode on the stalled and the collided ribosome in the disome structure as the one we propose for single stalled ribosomes. This would place the structured domains of two ZAKα molecules in direct vicinity of each other (Figure 6E), which supports the notion that this sensor responds to mRNA stasis (Figure 6F).

Ribosome-templated unfolding and transient dimerization underlies ZAKα kinase activation

ZAKα activation critically depends on activation loop auto-phosphorylation,[11,37] a process that most often occurs *in trans*.[38] We fused FL HA-tagged ZAKα or the kinase domain plus LZ fragment (amino acids 1–332) to FKBP12-F36V. To enforce dimerization, we treated transfected cells with the FKBP12-binding compound AP20718 (Figure 7A). For both constructs, this induced an autophosphorylation-associated mobility shift of ZAKα (Figures 7B and 7C), which was consistent with kinase activation under conditions of forced dimerization. For the 1–332 construct, AP20718 also activated p38 and JNK even in the absence of a ribotoxic stress signal (Figure 7B). FL ZAKα showed no AP20718-induced p38/JNK activation (Figure S6E), which suggests that additional structured domains impair downstream signaling. While ZAKα appears to be a predominantly monomeric protein, it does have a propensity for self-interaction. We could thus co-purify transiently transfected GFP-tagged ZAKα and strep-HA-tagged ZAKα (Figure S7A).

When presented with two ZAKα molecules, AF3 suggested a dimer conformation with the two kinase domains positioned face to face (which is consistent with activation loop trans-autophosphorylation) and guided by the two LZs (Figures S7B and S7C). It furthermore indicated that the YLD is a self-interacting domain that may stabilize or favor a dimerized state (Figures S7B–S7D). Deletion of the YLD (ZAKα ΔYLD) yielded a mutant severely compromised for activation 15 min after LTM, "Ani L," and "Ani H" (Figures 7D and S7E), yet relatively proficient in RSR signaling at 1 h (Figure S7F), which is consistent with a hypomorphic phenotype. This was not due to decreased ribosome binding (sucrose cushion, iCLIP; Figures S7G and S7H). A composite point mutant (ZAKα K458Y W500D) that was predicted[39] to abolish YLD dimerization behaved similarly (Figures 7D and S7I). We also deleted the LZ in its entirety (ZAKα ΔLZ), and this mutant behaved as a null mutant despite being proficient in ribosome binding (Figures 7D, 7E, and S7J).We conclude that the YLD is important and the LZ is critical for translating ribotoxic stress sensing by ZAKα into kinase activation, which is likely by enabling an activation-competent dimeric or oligomeric complex. We previously reported that a patient-derived (ZAKα F368C[40]), as well as an engineered (ZAKα W347S) point mutation in the SAM domain, were associated with constitutive ZAKα activity in the absence of an exogenous ribotoxic stress insult.[24] In aggregate, all of these results imply that the YLD and LZ domains are positively acting mediators of ZAKα

(B) As in (A), except that normalized crosslink numbers were plotted.
(C) U2OS/ΔZAK cells stably rescued with strep-HA-tagged ZAKα WT and mutants (top) were treated with ani (1 μM, 1 h), and lysates were ultracentrifuged through sucrose cushions (bottom). WCE and ribosome pellets were analyzed by immunoblotting.
(D) Mean distribution of relative Ribo-seq counts across transcripts (*n* = 14,691) under conditions from (A).
(E) Ribosome position expressed relative to start and stop codons from analysis in (D).
(F) Normalized ZAKα mRNA crosslinks around start (left) and stop (right) codons at low/high resolution.
See also Figures S4 and S5.

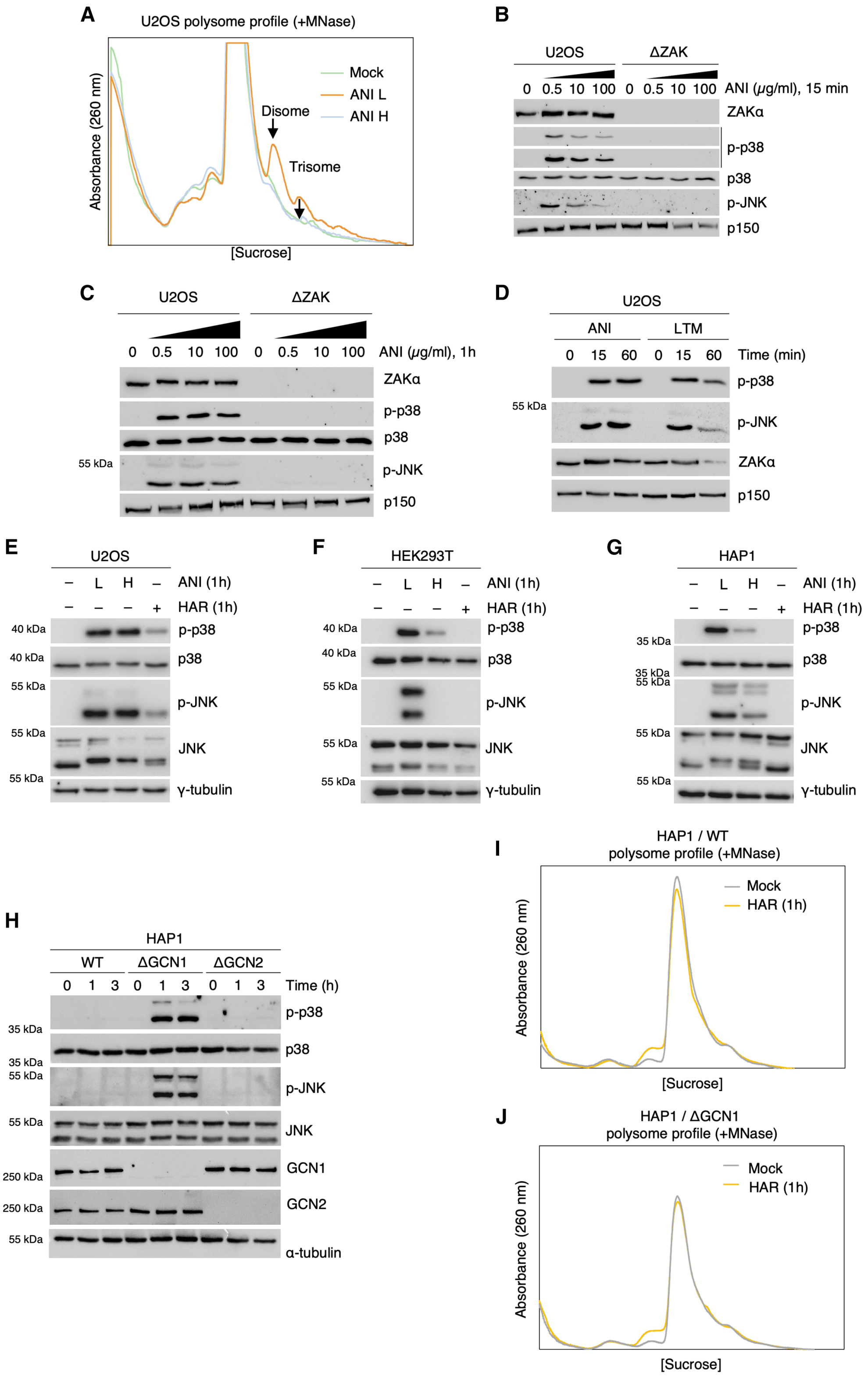

Figure 5. Activation of ZAKα can occur in the absence of a ribosome collision

(A) MNase assay to measure ribosome collision in U2OS cells treated with mock and ani L, 0.19 μM or ani H, 76 μM (15 min).

(B) U2OS and ΔZAK cells were treated with increasing concentrations of ani (1, 10, and 100 μg/mL) for 15 min. Lysates were analyzed by immunoblotting.

(legend continued on next page)

dimerization and activation, while the SAM domain exerts negative regulation over this activity in the absence of ribosome binding and ribotoxic stress signals. One of the two SLIMs that ZAKα inserts into RACK1 upon ribosome binding is located right after the SAM domain, which suggests that this interaction (Figures 1E–1G) could restrain an inhibitory influence of the SAM domain on the neighboring and critical LZ. To test this, we conducted molecular dynamics simulations of FL ZAKα either in isolation or complexed with RACK1 (in both cases, an AF3 model was used as the starting point [Figure 7F]). We simulated in triplicate for 100 ns using 2 fs time steps. Inspection of the resulting videos highlighted that the SAM vigorously explored the space around the distal part of the LZ, which was likely prohibiting the formation of a dimeric state that is competent for alignment of the two kinase domains (Video S1). In the RACK1-complexed state, however, the SAM domain was effectively sequestered on the surface of RACK1 and restrained from influencing the LZ (Figures 7G and S7K; Video S2). We furthermore computed the minimum distance between any residue in SAM and any residue in the distal (SAM-neighboring) part of the LZ over time from all simulation replicates. This analysis demonstrated the stochastic and transient nature of LZ-SAM proximity when ZAKα was simulated alone, which is a behavior that was completely obfuscated by binding to RACK1 (Figure 7H). Finally, we computed the Jensen-Shannon divergence (JSD)[41] between ZAKα residue-residue distances in ZAKα alone vs. ZAKα-RACK1 simulations (Figure 7I). This metric indicates the similarity between two probability distributions and also highlights how the presence of RACK1 negatively affected the proximity over time of the distal part of LZ and SAM. In aggregate, our previous work, biochemical analyses, and molecular dynamics simulations are consistent with a model in which intramolecular crosstalk between the negatively acting SAM domain and the essential LZ prohibits unscheduled ZAKα activation. It also suggests an explanation for how this auto-regulatory principle is bypassed by prolonged or stable binding of ZAKα to the ribosome.

DISCUSSION

Here, we used AF3 modeling (protein) combined with iCLIP (RNA) to unravel the ribosome-binding mode of the ribotoxic stress sensor ZAKα. We furthermore provide several lines of evidence for activation of ZAKα by single stalled ribosomes (LTM, HAR, and high anisomycin, etc.) (Figure 5) in addition to collided ribosomes. It has been experimentally shown that ribosome collision results in stronger ZAKα activation than does ribosome stalling.[14,24,31] A likely mechanistic explanation is that ZAKα is more prone to dimerize at a collision interface, given the very close vicinity of additional low-affinity (mRNA-independent) binding sites on the stalled ribosome in a disome (Figure 6E). During the revision phase of our work, an article with a cryo-electron microscopy (cryo-EM)-derived structure of parts of ZAKα bound to the interface of collided ribosomes was published. This structure validates the three ribosomal protein interactions between ZAKα, RACK1, and RPS27 that we report here and further shows dimerization through the SAM domains of two ZAKα monomers.[42] Our work highlights that activation is associated with trans-autophosphorylation and thus obligatory self-interaction of ZAKα, which is most likely brought about by the stabilized binding of a ZAKα monomer to RACK1. Molecular dynamics simulations suggest that this interaction sequesters the SAM domain of ZAKα from exerting a negative influence on the formation of an activation-competent dimer coordinated by the LZ domain. This activation mechanism resembles that of another mixed-lineage kinase family member, MLK3. In the monomeric and auto-inhibited form of this kinase, the LZ is occluded by an intramolecular interaction between an SH3 domain and a proline residue.[43] In the case of MLK3, the binding partner that disrupts this auto-inhibited state is GTP-bound Cdc42/Rac1 that can occupy a CRIB (Cdc42- and Rac-interactive binding) motif adjacent to the negatively acting proline. Once this intramolecular interaction is disrupted, the LZ of MLK3 mediates dimerization, trans-autophosphorylation, and activation of MLK3.[44]

Binding of ZAKα to RACK1 depends on a virtually identical interaction mode to that recently described for the mRNA stabilizing factors LARP4 and LARP4B.[29] One of the two sites are also occupied by the highly abundant factor SERBP1.[45] Thus, the SLIMs are highly similar between these proteins, and they all appear to occupy the same interaction cleft(s) in RACK1. The composite binding site comprised of RPS27 protein and 18S helix-26 is also occupied by factors other than ZAKα. One of these is the initiation factor complex EIF3, which both masks this site completely and blocks access to the mRNA exit channel.[46] In addition, several IRESs and the proteins USP10 and G3BP1 are known to bind 18S helix-26.[47,48]

Central to ribosome binding and ribotoxic stress sensing are the S and CTD domains of ZAKα, which underlie the observed mRNA crosslinking. The CTD contains a large number of lysine and arginine residues that when mutated to alanines render this domain unfunctional. S domain function depends on the integrity of at least one of three peptide repeats with the consensus sequence *R*G*R*YXX*R*/*K*.[33] These repeats are dispersed across the linker connecting the RACK1 and RPS27-helix-26 binding sites in the ZAKα C terminus. We infer from the above that the positively charged amino acids in CTD as well as S (highlighted with italics and mutated to alanines in our mutant) connect to mRNA protruding from the ribosomal mRNA exit channel. In sum, our modeling and experimental validation places ZAKα at the mRNA exit channel where it monitors ribosome processivity. By surveying this feature, ZAKα can detect stalling and collision through a common mechanism.

(C) As in (B), except that cells were treated for 1 h.
(D) U2OS cells were treated with ribotoxic stress agents ani (1 μM) and lactimidomycin (LTM, 1 μM) for the indicated times. Lysates were analyzed as in (B).
(E–G) (E) U2OS, (F) HEK293, and (G) HAP1 cells were treated with ani L, ani H, or harringtonine (HAR, 10 μM) for 1 h and analyzed as in (B).
(H) HAP1 WT, ΔGCN1, and ΔGCN2 cells were treated with HAR (10 μM) for the indicated times. Lysates were analyzed as in (B).
(I and J) lysates from (H) were treated with MNase and analyzed as in (A).

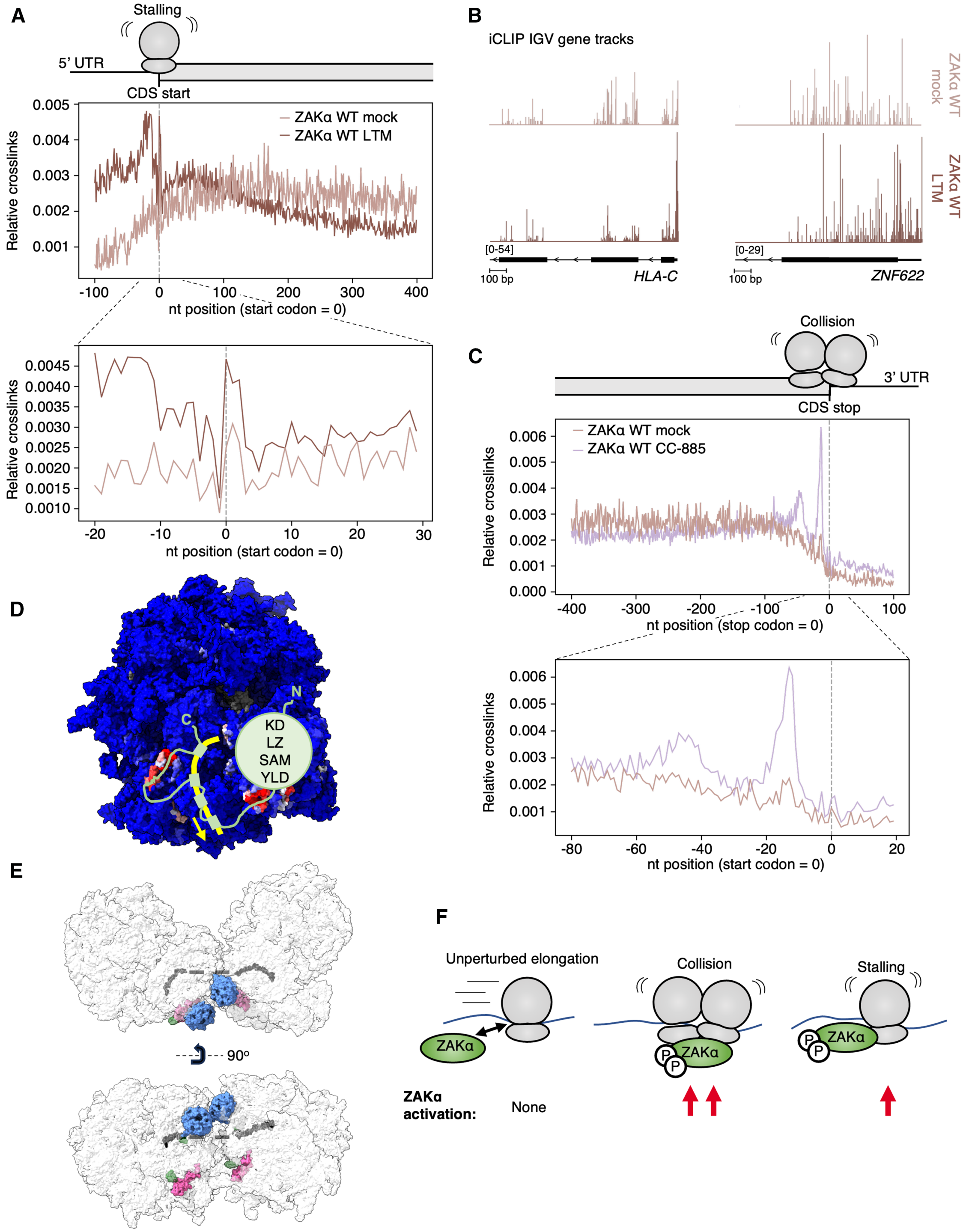

Figure 6. A unifying model for sensing of stalled and collided ribosomes by ZAKα

(A) Normalized ZAKα mRNA crosslinks around the start codon after LTM (15 μM, 1 h) at low/high resolution.

(B) Exon-intron structure of start (right side) of HLA-C and ZNF622 transcripts and iCLIP read tracks for mock and LTM conditions, which indicates increased crosslinking to 5′ UTR.

(C) As in (A), but around the stop codon and from cells treated with the GSPT1/ERF3 degrader CC-885 (1 μM, 6 h).

(D) The model shows ZAKα C terminus anchors on two ribosomal surface patches while peptide motifs contact mRNA around the exit channel; during elongation these contact points separate and ZAKα dissociates from the ribosome. Green sphere, folded N-terminal domains.

(legend continued on next page)

CellPress
OPEN ACCESS

Limitations of the study

Our iCLIP data highlight a near-complete loss of mRNA crosslinking for the ZAKα R→A ΔCTD (mRNA-binding-deficient mutant) but only a reduced mRNA crosslinking for the ZAKα Δ417–422 and Δ611–617 (RACK binding-deficient mutant) (Figures S5B and 2G vs. Figure S2F). We also document that the last 100 amino acids of ZAKα binds to a random unstructured RNA probe with EMSA (Figures S6C and S6D). Further *in vitro* studies (e.g., NMR and EMSA) to characterize the direct mRNA-binding properties of ZAKα S and CTD domains should be performed in future research.

RESOURCE AVAILABILITY

Lead contact

Further information and requests for resources and reagents should be directed to and will be fulfilled by the lead contact, Simon Bekker-Jensen (sbj@sund.ku.dk).

Materials availability

Plasmids, cell lines, and other materials generated in this study are available upon reasonable request to the lead contact.

Data and code availability

Demultiplexed iCLIP fastq files and processed crosslinking BED files have been deposited in NCBI GEO archive under accession GEO: GSE292064. Demultiplexed Ribo-seq fastq files and A-site count tables (.sga) have been deposited in NCBI GEO under accession GEO: GSE314163. Original image-based data files have been deposited in Mendeley Data (DOI 10.17632/tr4wzcp7h4.1). All deposited data are publicly available as of the date of publication.

The code used to map AF3 contact probability scores and iCLIP crosslinks to a structure is available at Zenodo (DOI 10.5281/zenodo.19709634).

Any additional information required to reanalyze the data reported in this paper is available from the lead contact upon request.

ACKNOWLEDGMENTS

We thank Dr. Eric Bennett (University of California, San Diego, USA) for reagents, and we wish to acknowledge the SUND Genomics Platform (University of Copenhagen, Denmark) for the sequencing of iCLIP libraries. G.A. and M.R.C. thank the Centre for Biomolecular Spectroscopy (for ITC equipment), which was funded by the Wellcome Trust and BBSRC. Work in the Bekker-Jensen lab was supported by the European Research Council (ERC) under the European Union's Horizon 2020 research and innovation program (grant agreement 863911—PHYRIST), the Independent Research Fund Denmark (grant no. 3101-00344B), and the LEO Foundation (grant no. LF-OC-23-001458). The Center for Gene Expression (CGEN) is a Center of Excellence funded by The National Danish Research Foundation (grant no. DNRF166). Work in the Heick Jensen lab was supported by the Novo Nordisk Foundation (ExoAdapt grant no. 31199). A.B. was supported by the Marie Curie Individual Fellowship (EXOonRNA, grant no. 101026781) and Lundbeck Foundation Experiment Grant (grant no. R346-2020-1610). G.A. and M.R.C. are supported by a Leverhulme Trust and a BBSRC grant, grant nos. RPG-2020-264 and UKRI1921, respectively. Collaboration between S.B.J. and D.G. was funded by the Swiss National Science Foundation International Co-Investigator Scheme (grant no. 10002692).

AUTHOR CONTRIBUTIONS

A.C.V., K.M., A.S.R., C.P., L.R., Q.C., M.R., X.S., D.H., and M.B. performed the biochemical and cell biological experiments. J.F.M. developed the AlphaFold analysis platform and performed bioinformatic analysis and the molecular dynamics simulations. Z.W. and A.B. conducted the iCLIP experiments. G.A. performed the ITC experiments. S.C. conducted the ribosome profiling experiments. P.T., J.Q.S., D.G., M.R.C., T.H.J., and S.B.-J. supervised the research. A.C.V., J.F.M., and S.B.-J. conceived the project. S.B.-J. wrote the manuscript. All authors discussed the results and commented on the manuscript.

DECLARATION OF INTERESTS

The authors have no positions, patents, or financial interests to declare.

STAR★METHODS

Detailed methods are provided in the online version of this paper and include the following:

- KEY RESOURCES TABLE
- EXPERIMENTAL MODEL AND STUDY PARTICIPANT DETAILS
  - Cell lines
  - Bacterial strains
- METHOD DETAILS
  - Plasmids
  - Cell culture and reagents
  - Lentivirus production and transduction
  - Phosphatase treatment
  - Western blotting, pull-down and antibodies
  - Sucrose cushions
  - Ribo-seq libraries
  - Ribo-seq data processing
  - Polysome profiling
  - Protein expression and purification
  - Isothermal titration calorimetry (ITC)
  - UV crosslinking, extraction, and immunoprecipitation of crosslinked ZAKα -RNA complexes
  - Electrophoretic mobility shift assay (EMSA)
  - iCLIP library preparation and sequencing
  - Alignment to reference genome and crosslink mapping
  - Crosslinked features quantification
  - Ribosomal RNA crosslinking visualization
  - Metagene profiles of whole transcripts
  - Metagene profiles around start and stop codons
  - Visualization of Alphafold scores and iCLIP values in ChimeraX
  - Molecular dynamics simulation
- QUANTIFICATION AND STATISTICAL ANALYSIS

SUPPLEMENTAL INFORMATION

Supplemental information can be found online at https://doi.org/10.1016/j.molcel.2026.04.034.

Received: May 26, 2025
Revised: December 19, 2025
Accepted: April 30, 2026
Published: May 29, 2026

(E) Representation of a collided human ribosome structure (PDB: 7QVP) with RACK1 (blue), RPS27 (magenta), 18S-helix 26 (green), and mRNA (gray) highlighted.
(F) Model for sensing of a common ribotoxic stress signal by ZAKα based on mRNA stasis elicited by stalled and collided ribosomes. In this model, activation of ZAKα is determined by prolonged and/or stable ribosome interaction.
(A and C) Lengths of mRNA fragments protected by stalled and collided ribosomes are indicated.
See also Figures S5 and S6.

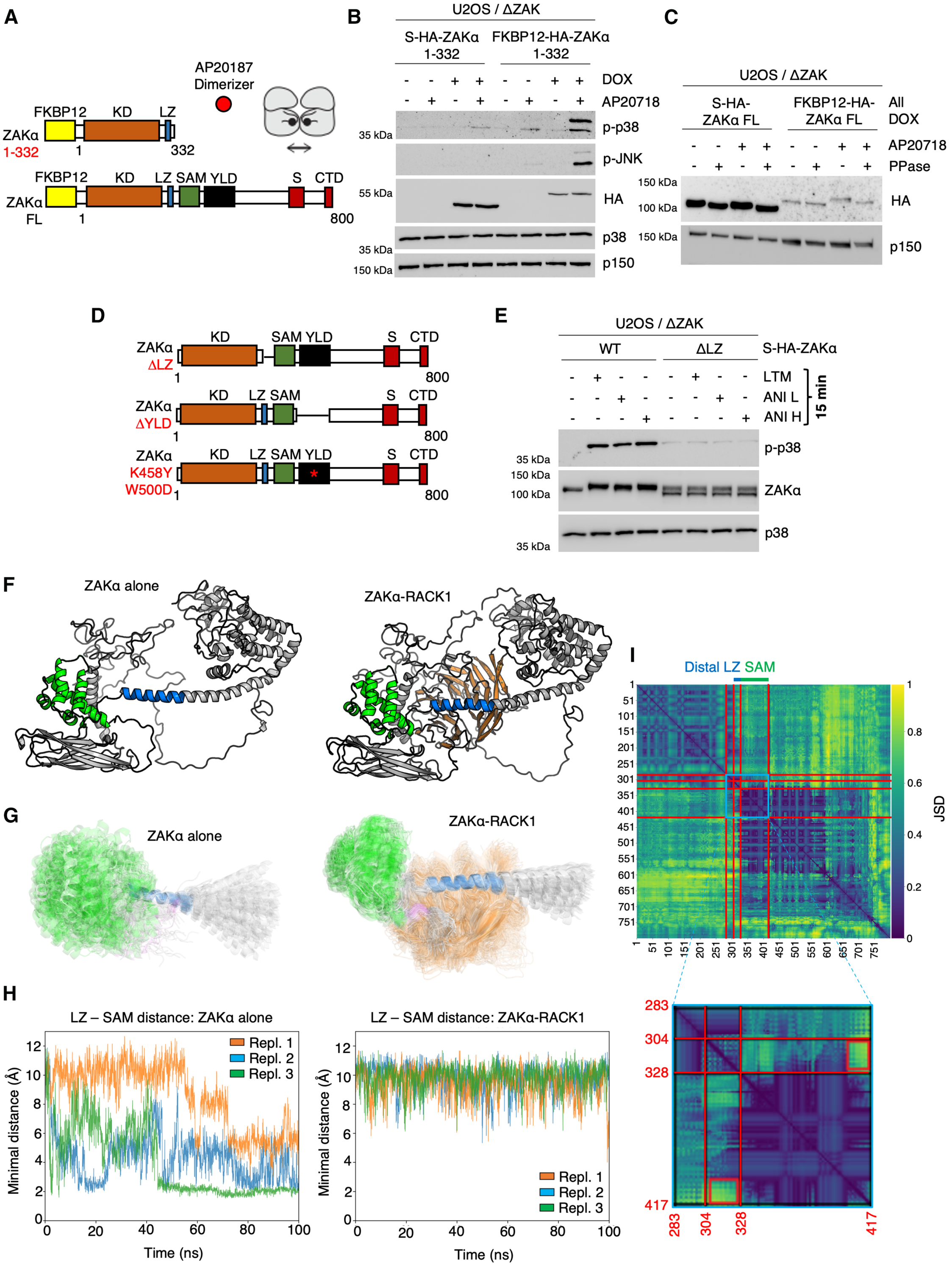

Figure 7. Ribosome-bound ZAKα is competent for dimerization and activation

(A) Experimental strategy for forced dimerization of ZAKα monomers. Full-length (FL) and truncated (aa 1–332) ZAKα were N-terminally fused to FKBP12-F36V, which can be forcibly dimerized using the bivalent molecule AP20187.

(B) U2OS/ΔZAK cells expressing FKBP12-HA-ZAKα (1–332) were treated with doxycycline (DOX) overnight and AP20187 (50 nM, 1 h). Lysates were analyzed by immunoblotting.

(legend continued on next page)

REFERENCES

1. Vind, A.C., Zhong, F.L., and Bekker-Jensen, S. (2024). Death by ribosome. Trends Cell Biol. *35*, 615–626. https://doi.org/10.1016/j.tcb.2024.10.013.
2. Iordanov, M.S., Pribnow, D., Magun, J.L., Dinh, T.H., Pearson, J.A., and Magun, B.E. (1998). Ultraviolet radiation triggers the ribotoxic stress response in mammalian cells. J. Biol. Chem. *273*, 15794–15803. https://doi.org/10.1074/jbc.273.25.15794.
3. Snieckute, G., Ryder, L., Vind, A.C., Wu, Z., Arendrup, F.S., Stoneley, M., Chamois, S., Martinez-Val, A., Leleu, M., Dreos, R., et al. (2023). ROS-induced ribosome impairment underlies ZAKalpha-mediated metabolic decline in obesity and aging. Science *382*, eadf3208. https://doi.org/10.1126/science.adf3208.
4. Iordanov, M.S., Pribnow, D., Magun, J.L., Dinh, T.H., Pearson, J.A., Chen, S.L., and Magun, B.E. (1997). Ribotoxic stress response: activation of the stress-activated protein kinase JNK1 by inhibitors of the peptidyl transferase reaction and by sequence-specific RNA damage to the alpha-sarcin/ricin loop in the 28S rRNA. Mol. Cell. Biol. *17*, 3373–3381. https://doi.org/10.1128/MCB.17.6.3373.
5. Zhang, P., Hu, X., Li, Z., Liu, Q., Liu, L., Jin, Y., Liu, S., Zhao, X., Wang, J., Hao, D., et al. (2024). Schlafen 11 triggers innate immune responses through its ribonuclease activity upon detection of single-stranded DNA. Sci. Immunol. *9*, eadj5465. https://doi.org/10.1126/sciimmunol.adj5465.
6. Boon, N.J., Oliveira, R.A., Körner, P.R., Kochavi, A., Mertens, S., Malka, Y., Voogd, R., van der Horst, S.E.M., Huismans, M.A., Smabers, L.P., et al. (2024). DNA damage induces p53-independent apoptosis through ribosome stalling. Science *384*, 785–792. https://doi.org/10.1126/science.adh7950.
7. Xi, J., Snieckute, G., Martínez, J.F., Arendrup, F.S.W., Asthana, A., Gaughan, C., Lund, A.H., Bekker-Jensen, S., and Silverman, R.H. (2024). Initiation of a ZAKalpha-dependent ribotoxic stress response by the innate immunity endoribonuclease RNase L. Cell Rep. *43*, 113998. https://doi.org/10.1016/j.celrep.2024.113998.
8. Karasik, A., Lorenzi, H.A., DePass, A.V., and Guydosh, N.R. (2024). Endonucleolytic RNA cleavage drives changes in gene expression during the innate immune response. Cell Rep. *43*, 114287. https://doi.org/10.1016/j.celrep.2024.114287.
9. Vind, A.C., Wu, Z., Firdaus, M.J., Snieckute, G., Toh, G.A., Jessen, M., Martínez, J.F., Haahr, P., Andersen, T.L., Blasius, M., et al. (2024). The ribotoxic stress response drives acute inflammation, cell death, and epidermal thickening in UV-irradiated skin in vivo. Mol. Cell *84*, 4774–4789.e9. https://doi.org/10.1016/j.molcel.2024.10.044.
10. Robinson, K.S., Toh, G.A., Rozario, P., Chua, R., Bauernfried, S., Sun, Z., Firdaus, M.J., Bayat, S., Nadkarni, R., Poh, Z.S., et al. (2022). ZAKalpha-driven ribotoxic stress response activates the human NLRP1 inflammasome. Science *377*, 328–335. https://doi.org/10.1126/science.abl6324.
11. Sinha, N.K., McKenney, C., Yeow, Z.Y., Li, J.J., Nam, K.H., Yaron-Barir, T.M., Johnson, J.L., Huntsman, E.M., Cantley, L.C., Ordureau, A., et al. (2024). The ribotoxic stress response drives UV-mediated cell death. Cell *187*, 3652–3670.e40. https://doi.org/10.1016/j.cell.2024.05.018.
12. Subramanian, A., Wang, L., Moss, T., Voorhies, M., Sangwan, S., Stevenson, E., Pulido, E.H., Kwok, S., Chalkley, R.J., Li, K.H., et al. (2023). A Legionella toxin exhibits tRNA mimicry and glycosyl transferase activity to target the translation machinery and trigger a ribotoxic stress response. Nat. Cell Biol. *25*, 1600–1615. https://doi.org/10.1038/s41556-023-01248-z.
13. McKenney, C., Lendner, Y., Guerrero Zuniga, A., Sinha, N., Veresko, B., Aikin, T.J., and Regot, S. (2024). CDK4/6 activity is required during G(2) arrest to prevent stress-induced endoreplication. Science *384*, eadi2421. https://doi.org/10.1126/science.adi2421.
14. Wu, C.C.C., Peterson, A., Zinshteyn, B., Regot, S., and Green, R. (2020). Ribosome Collisions Trigger General Stress Responses to Regulate Cell Fate. Cell *182*, 404–416.e14. https://doi.org/10.1016/j.cell.2020.06.006.
15. Inada, T., and Beckmann, R. (2024). Mechanisms of Translation-coupled Quality Control. J. Mol. Biol. *436*, 168496. https://doi.org/10.1016/j.jmb.2024.168496.
16. Müller, M.B.D., Becker, T., Denk, T., Hashimoto, S., Inada, T., and Beckmann, R. (2025). The ribosome as a platform to coordinate mRNA decay. Nucleic Acids Res. *53*, gkaf049. https://doi.org/10.1093/nar/gkaf049.
17. Pochopien, A.A., Beckert, B., Kasvandik, S., Berninghausen, O., Beckmann, R., Tenson, T., and Wilson, D.N. (2021). Structure of Gcn1 bound to stalled and colliding 80S ribosomes. Proc. Natl. Acad. Sci. USA *118*, e2022756118. https://doi.org/10.1073/pnas.2022756118.
18. Narita, M., Denk, T., Matsuo, Y., Sugiyama, T., Kikuguchi, C., Ito, S., Sato, N., Suzuki, T., Hashimoto, S., Machová, I., et al. (2022). A distinct mammalian disome collision interface harbors K63-linked polyubiquitination of uS10 to trigger hRQT-mediated subunit dissociation. Nat. Commun. *13*, 6411. https://doi.org/10.1038/s41467-022-34097-9.
19. Kim, K.Q., Li, J.J., Nanjaraj Urs, A.N., Pacheco, M.E., Lasehinde, V., Denk, T., Tesina, P., Tomomatsu, S., Matsuo, Y., McDonald, E., et al. (2024). Multiprotein bridging factor 1 is required for robust activation of the integrated stress response on collided ribosomes. Mol. Cell *84*, 4594–4611.e9. https://doi.org/10.1016/j.molcel.2024.10.029.
20. Park, E.N., Mackens-Kiani, T., Berhane, R., Esser, H., Erdenebat, C., Burroughs, A.M., Berninghausen, O., Aravind, L., Beckmann, R., Green, R., et al. (2024). B. subtilis MutS2 splits stalled ribosomes into subunits without mRNA cleavage. EMBO J. *43*, 484–506. https://doi.org/10.1038/s44318-023-00010-3.
21. Campbell, A., Esser, H.F., Burroughs, A.M., Berninghausen, O., Aravind, L., Becker, T., Green, R., Beckmann, R., and Buskirk, A.R. (2025). The RNA helicase HrpA rescues collided ribosomes in E. coli. Mol. Cell *85*, 999–1007.e7. https://doi.org/10.1016/j.molcel.2025.01.018.
22. Saito, K., Kratzat, H., Campbell, A., Buschauer, R., Burroughs, A.M., Berninghausen, O., Aravind, L., Green, R., Beckmann, R., and Buskirk, A.R. (2022). Ribosome collisions induce mRNA cleavage and ribosome

(C) U2OS/ΔZAK cells expressing FL FKBP12-HA-ZAKα from (A) were treated as in (B). Lysates were treated with λ phosphatase (PPase) for 30 min (room temperature) and analyzed as in (B).
(D) Schematic of ZAKα mutants with deletion of LZ (ΔLZ) and YLD (ΔYLD) and a composite point mutant of the YLD domain (K458Y and W500D).
(E) U2OS/ΔZAK cells stably rescued with WT and ΔLZ forms of strep-HA-tagged ZAKα were treated with ribotoxic stress agents ani L (0.19 μM), ani H (76 μM), or LTM (1 μM) for 15 min and analyzed as in (B).
(F) Structure at t = 0 for ZAKα alone (left) and ZAKα-RACK1 (right) molecular dynamics (MD) simulations. ZAKα residues 336 to 417 (SAM domain, green), 309 to 329 (C-terminal part of LZ, blue), and RACK1 (orange) are highlighted.
(G) Structural ensemble samples from the ZAKα alone (left) and ZAKα-RACK1 (right) MD simulations colored as in (F). In addition, ZAKα residues 418 to 422 (RACK1-binding SLIM, pink) are highlighted. For clarity, only residues 286–425 are shown, and FL ensembles are shown in Figure S7K.
(H) Minimum distance in angstroms (Å) between ZAKα residues 309 to 329 and residues 336 to 417 per replicate in the ZAKα alone (left) and ZAKα-RACK1 (right) MD simulations.
(I) Jensen-Shannon divergence (JSD) between ZAKα residue-residue distances in ZAKα alone vs. ZAKα-RACK1 100-ns MD simulations. Red lines indicate residues 283, 304, 328, and 417; red squares highlight JSD between residues 400–417 and 304–328. Repl, replicates.
See also Figures S6 and S7.; Videos S1 and S2.

rescue in bacteria. Nature *603*, 503–508. https://doi.org/10.1038/s41586-022-04416-7.

23. Snieckute, G., Genzor, A.V., Vind, A.C., Ryder, L., Stoneley, M., Chamois, S., Dreos, R., Nordgaard, C., Sass, F., Blasius, M., et al. (2022). Ribosome stalling is a signal for metabolic regulation by the ribotoxic stress response. Cell Metab. *34*, 2036–2046.e8. https://doi.org/10.1016/j.cmet.2022.10.011.
24. Vind, A.C., Snieckute, G., Blasius, M., Tiedje, C., Krogh, N., Bekker-Jensen, D.B., Andersen, K.L., Nordgaard, C., Tollenaere, M.A.X., Lund, A.H., et al. (2020). ZAKalpha Recognizes Stalled Ribosomes through Partially Redundant Sensor Domains. Mol. Cell *78*, 700–713.e7. https://doi.org/10.1016/j.molcel.2020.03.021.
25. Abramson, J., Adler, J., Dunger, J., Evans, R., Green, T., Pritzel, A., Ronneberger, O., Willmore, L., Ballard, A.J., Bambrick, J., et al. (2024). Accurate structure prediction of biomolecular interactions with AlphaFold 3. Nature *630*, 493–500. https://doi.org/10.1038/s41586-024-07487-w.
26. Milacic, M., Beavers, D., Conley, P., Gong, C., Gillespie, M., Griss, J., Haw, R., Jassal, B., Matthews, L., May, B., et al. (2024). The Reactome Pathway Knowledgebase 2024. Nucleic Acids Res. *52*, D672–D678. https://doi.org/10.1093/nar/gkad1025.
27. Khatter, H., Myasnikov, A.G., Natchiar, S.K., and Klaholz, B.P. (2015). Structure of the human 80S ribosome. Nature *520*, 640–645. https://doi.org/10.1038/nature14427.
28. Brockmann, M., Blomen, V.A., Nieuwenhuis, J., Stickel, E., Raaben, M., Bleijerveld, O.B., Altelaar, A.F.M., Jae, L.T., and Brummelkamp, T.R. (2017). Genetic wiring maps of single-cell protein states reveal an off-switch for GPCR signalling. Nature *546*, 307–311. https://doi.org/10.1038/nature22376.
29. Ranjan, A., Mattijssen, S., Charlly, N., Gallardo, I.C., Pitman, L.F., Coleman, J.C., Conte, M.R., and Maraia, R.J. (2025). The short conserved region-2 of LARP4 interacts with ribosome-associated RACK1 and promotes translation. Nucleic Acids Res. *53*, gkaf053. https://doi.org/10.1093/nar/gkaf053.
30. Xiang, J.S., Schafer, D.M., Rothamel, K.L., and Yeo, G.W. (2024). Decoding protein-RNA interactions using CLIP-based methodologies. Nat. Rev. Genet. *25*, 879–895. https://doi.org/10.1038/s41576-024-00749-3.
31. Stoneley, M., Harvey, R.F., Mulroney, T.E., Mordue, R., Jukes-Jones, R., Cain, K., Lilley, K.S., Sawarkar, R., and Willis, A.E. (2022). Unresolved stalled ribosome complexes restrict cell-cycle progression after genotoxic stress. Mol. Cell *82*, 1557–1572.e7. https://doi.org/10.1016/j.molcel.2022.01.019.
32. Fedry, J., Silva, J., Vanevic, M., Fronik, S., Mechulam, Y., Schmitt, E., des Georges, A., Faller, W.J., and Förster, F. (2024). Visualization of translation reorganization upon persistent ribosome collision stress in mammalian cells. Mol. Cell *84*, 1078–1089.e4. https://doi.org/10.1016/j.molcel.2024.01.015.
33. Johansen, V.B.I., Snieckute, G., Vind, A.C., Blasius, M., and Bekker-Jensen, S. (2023). Computational and Functional Analysis of Structural Features in the ZAKalpha Kinase. Cells *12*, 969. https://doi.org/10.3390/cells12060969.
34. Ahmed, N., Sormanni, P., Ciryam, P., Vendruscolo, M., Dobson, C.M., and O'Brien, E.P. (2019). Identifying A- and P-site locations on ribosome-protected mRNA fragments using Integer Programming. Sci. Rep. *9*, 6256. https://doi.org/10.1038/s41598-019-42348-x.
35. Nugent, P.J., Park, H., Wladyka, C.L., Yelland, J.N., Sinha, S., Chen, K.Y., Bynum, C., Quarterman, G., Lee, S.C., Hsieh, A.C., et al. (2025). Decoding post-transcriptional regulatory networks by RNA-linked CRISPR screening in human cells. Nat. Methods *22*, 1237–1246. https://doi.org/10.1038/s41592-025-02702-6.
36. Matyskiela, M.E., Lu, G., Ito, T., Pagarigan, B., Lu, C.C., Miller, K., Fang, W., Wang, N.Y., Nguyen, D., Houston, J., et al. (2016). A novel cereblon modulator recruits GSPT1 to the CRL4(CRBN) ubiquitin ligase. Nature *535*, 252–257. https://doi.org/10.1038/nature18611.
37. Tosti, E., Waldbaum, L., Warshaw, G., Gross, E.A., and Ruggieri, R. (2004). The stress kinase MRK contributes to regulation of DNA damage checkpoints through a p38gamma-independent pathway. J. Biol. Chem. *279*, 47652–47660. https://doi.org/10.1074/jbc.M409961200.
38. Reinhardt, R., and Leonard, T.A. (2023). A critical evaluation of protein kinase regulation by activation loop autophosphorylation. eLife *12*, e88210. https://doi.org/10.7554/eLife.88210.
39. Zhou, Y., Pan, Q., Pires, D.E.V., Rodrigues, C.H.M., and Ascher, D.B. (2023). DDMut: predicting effects of mutations on protein stability using deep learning. Nucleic Acids Res. *51*, W122–W128. https://doi.org/10.1093/nar/gkad472.
40. Spielmann, M., Kakar, N., Tayebi, N., Leettola, C., Nürnberg, G., Sowada, N., Lupiáñez, D.G., Harabula, I., Flöttmann, R., Horn, D., et al. (2016). Exome sequencing and CRISPR/Cas genome editing identify mutations of ZAK as a cause of limb defects in humans and mice. Genome Res. *26*, 183–191. https://doi.org/10.1101/gr.199430.115.
41. Nielsen, F. (2020). On a Generalization of the Jensen-Shannon Divergence and the Jensen-Shannon Centroid. Entropy (Basel) *22*, 221. https://doi.org/10.3390/e22020221.
42. Huso, V.L., Niu, S., Catipovic, M.A., Saba, J.A., Denk, T., Park, E., Cheng, J., Berninghausen, O., Becker, T., Green, R., et al. (2025). ZAK activation at the collided ribosome. Nature *649*, 1051–1060. https://doi.org/10.1038/s41586-025-09772-8.
43. Zhang, H., and Gallo, K.A. (2001). Autoinhibition of mixed lineage kinase 3 through its Src homology 3 domain. J. Biol. Chem. *276*, 45598–45603. https://doi.org/10.1074/jbc.M107176200.
44. Rattanasinchai, C., and Gallo, K.A. (2016). MLK3 Signaling in Cancer Invasion. Cancers (Basel) *8*, 51. https://doi.org/10.3390/cancers8050051.
45. Zheng, W., Zhang, Y., Wang, J., Wang, S., Chai, P., Bailey, E.J., Zhu, C., Guo, W., Devarkar, S.C., Wu, S., et al. (2025). Visualizing the translation landscape in human cells at high resolution. Nat. Commun. *16*, 10757. https://doi.org/10.1038/s41467-025-65795-9.
46. Brito Querido, J., Sokabe, M., Díaz-López, I., Gordiyenko, Y., Fraser, C.S., and Ramakrishnan, V. (2024). The structure of a human translation initiation complex reveals two independent roles for the helicase eIF4A. Nat. Struct. Mol. Biol. *31*, 455–464. https://doi.org/10.1038/s41594-023-01196-0.
47. Yamamoto, H., Collier, M., Loerke, J., Ismer, J., Schmidt, A., Hilal, T., Sprink, T., Yamamoto, K., Mielke, T., Bürger, J., et al. (2015). Molecular architecture of the ribosome-bound Hepatitis C Virus internal ribosomal entry site RNA. EMBO J. *34*, 3042–3058. https://doi.org/10.15252/embj.201592469.
48. Meyer, C., Garzia, A., Morozov, P., Molina, H., and Tuschl, T. (2020). The G3BP1-Family-USP10 Deubiquitinase Complex Rescues Ubiquitinated 40S Subunits of Ribosomes Stalled in Translation from Lysosomal Degradation. Mol. Cell *77*, 1193–1205.e5. https://doi.org/10.1016/j.molcel.2019.12.024.
49. Dobin, A., Davis, C.A., Schlesinger, F., Drenkow, J., Zaleski, C., Jha, S., Batut, P., Chaisson, M., and Gingeras, T.R. (2013). STAR: ultrafast universal RNA-seq aligner. Bioinformatics *29*, 15–21. https://doi.org/10.1093/bioinformatics/bts635.
50. Park, C., and Walsh, D. (2022). RACK1 Regulates Poxvirus Protein Synthesis Independently of Its Role in Ribosome-Based Stress Signaling. J. Virol. *96*, e0109322. https://doi.org/10.1128/jvi.01093-22.
51. McGlincy, N.J., and Ingolia, N.T. (2017). Transcriptome-wide measurement of translation by ribosome profiling. Methods *126*, 112–129. https://doi.org/10.1016/j.ymeth.2017.05.028.
52. Ran, F.A., Hsu, P.D., Wright, J., Agarwala, V., Scott, D.A., and Zhang, F. (2013). Genome engineering using the CRISPR-Cas9 system. Nat. Protoc. *8*, 2281–2308. https://doi.org/10.1038/nprot.2013.143.

CellPress
OPEN ACCESS

53. Cong, L., Ran, F.A., Cox, D., Lin, S., Barretto, R., Habib, N., Hsu, P.D., Wu, X., Jiang, W., Marraffini, L.A., et al. (2013). Multiplex genome engineering using CRISPR/Cas systems. Science *339*, 819–823. https://doi.org/10.1126/science.1231143.

54. Stewart, S.A., Dykxhoorn, D.M., Palliser, D., Mizuno, H., Yu, E.Y., An, D.S., Sabatini, D.M., Chen, I.S.Y., Hahn, W.C., Sharp, P.A., et al. (2003). Lentivirus-delivered stable gene silencing by RNAi in primary cells. RNA *9*, 493–501. https://doi.org/10.1261/rna.2192803.

55. Wilkins, M.R., Gasteiger, E., Bairoch, A., Sanchez, J.C., Williams, K.L., Appel, R.D., and Hochstrasser, D.F. (1999). Protein identification and analysis tools in the ExPASy server. Methods Mol. Biol. *112*, 531–552. https://doi.org/10.1385/1-59259-584-7:531.

56. Li, F., Xing, X., Xiao, Z., Xu, G., and Yang, X. (2020). RiboMiner: a toolset for mining multi-dimensional features of the translatome with ribosome profiling data. BMC Bioinform. *21*, 340. https://doi.org/10.1186/s12859-020-03670-8.

57. Dunn, J.G., and Weissman, J.S. (2016). Plastid: nucleotide-resolution analysis of next-generation sequencing and genomics data. BMC Genomics *17*, 958. https://doi.org/10.1186/s12864-016-3278-x.

58. Quinlan, A.R., and Hall, I.M. (2010). BEDTools: a flexible suite of utilities for comparing genomic features. Bioinformatics *26*, 841–842. https://doi.org/10.1093/bioinformatics/btq033.

59. Li, H., Handsaker, B., Wysoker, A., Fennell, T., Ruan, J., Homer, N., Marth, G., Abecasis, G., and Durbin, R.; 1000 Genome Project Data Processing Subgroup (2009). The Sequence Alignment/Map format and SAMtools. Bioinformatics *25*, 2078–2079. https://doi.org/10.1093/bioinformatics/btp352.

60. Liao, Y., Smyth, G.K., and Shi, W. (2013). The Subread aligner: fast, accurate and scalable read mapping by seed-and-vote. Nucleic Acids Res. *41*, e108. https://doi.org/10.1093/nar/gkt214.

61. Vögele, M., Thomson, N.J., Truong, S.T., McAvity, J., Zachariae, U., and Dror, R.O. (2025). Systematic analysis of biomolecular conformational ensembles with PENSA. J. Chem. Phys. *162*, 014101. https://doi.org/10.1063/5.0235544.

62. Michaud-Agrawal, N., Denning, E.J., Woolf, T.B., and Beckstein, O. (2011). MDAnalysis: a toolkit for the analysis of molecular dynamics simulations. J. Comput. Chem. *32*, 2319–2327. https://doi.org/10.1002/jcc.21787.

63. Meng, E.C., Goddard, T.D., Pettersen, E.F., Couch, G.S., Pearson, Z.J., Morris, J.H., and Ferrin, T.E. (2023). UCSF ChimeraX: Tools for structure building and analysis. Protein Sci. *32*, e4792. https://doi.org/10.1002/pro.4792.

64. Liu, H., and Naismith, J.H. (2008). An efficient one-step site-directed deletion, insertion, single and multiple-site plasmid mutagenesis protocol. BMC Biotechnol. *8*, 91. https://doi.org/10.1186/1472-6750-8-91.

65. Jha, S., Rollins, M.G., Fuchs, G., Procter, D.J., Hall, E.A., Cozzolino, K., Sarnow, P., Savas, J.N., and Walsh, D. (2017). Trans-kingdom mimicry underlies ribosome customization by a poxvirus kinase. Nature *546*, 651–655. https://doi.org/10.1038/nature22814.

66. Janich, P., Arpat, A.B., Castelo-Szekely, V., Lopes, M., and Gatfield, D. (2015). Ribosome profiling reveals the rhythmic liver translatome and circadian clock regulation by upstream open reading frames. Genome Res. *25*, 1848–1859. https://doi.org/10.1101/gr.195404.115.

67. Ruiz Carrillo, D., Chandrasekaran, R., Nilsson, M., Cornvik, T., Liew, C.W., Tan, S.M., and Lescar, J. (2012). Structure of human Rack1 protein at a resolution of 2.45 A. Acta Crystallogr. Sect. F Struct. Biol. Cryst. Commun. *68*, 867–872. https://doi.org/10.1107/S1744309112027480.

68. Buchbender, A., Mutter, H., Sutandy, F.X.R., Körtel, N., Hänel, H., Busch, A., Ebersberger, S., and König, J. (2020). Improved library preparation with the new iCLIP2 protocol. Methods *178*, 33–48. https://doi.org/10.1016/j.ymeth.2019.10.003.

69. Cordiner, R.A., Dou, Y., Thomsen, R., Bugai, A., Granneman, S., and Heick Jensen, T. (2023). Temporal-iCLIP captures co-transcriptional RNA-protein interactions. Nat. Commun. *14*, 696. https://doi.org/10.1038/s41467-023-36345-y.

70. Busch, A., Brüggemann, M., Ebersberger, S., and Zarnack, K. (2020). iCLIP data analysis: A complete pipeline from sequencing reads to RBP binding sites. Methods *178*, 49–62. https://doi.org/10.1016/j.ymeth.2019.11.008.

71. Pronk, S., Páll, S., Schulz, R., Larsson, P., Bjelkmar, P., Apostolov, R., Shirts, M.R., Smith, J.C., Kasson, P.M., van der Spoel, D., et al. (2013). GROMACS 4.5: a high throughput and highly parallel open source molecular simulation toolkit. Bioinformatics *29*, 845–854. https://doi.org/10.1093/bioinformatics/btt055.

72. Huang, J., Rauscher, S., Nawrocki, G., Ran, T., Feig, M., de Groot, B.L., Grubmüller, H., and MacKerell, A.D., Jr. (2017). CHARMM36m: an improved force field for folded and intrinsically disordered proteins. Nat. Methods *14*, 71–73. https://doi.org/10.1038/nmeth.4067.

73. Harris, C.R., Millman, K.J., van der Walt, S.J., Gommers, R., Virtanen, P., Cournapeau, D., Wieser, E., Taylor, J., Berg, S., Smith, N.J., et al. (2020). Array programming with NumPy. Nature *585*, 357–362. https://doi.org/10.1038/s41586-020-2649-2.

STAR★METHODS

KEY RESOURCES TABLE

REAGENT or RESOURCE	SOURCE	IDENTIFIER
**Antibodies**		
Rabbit polyclonal anti-ZAKα	Bethyl	Cat# A301-993A; RRID: AB_1576612
Mouse monoclonal anti-phospho-p38	Cell Signaling	Cat# 9216; RRID: AB_331296
Rabbit monoclonal anti-phospho-p38	Cell Signaling	Cat# 4511S; RRID: AB_2139682
Rabbit polyclonal antibody anti-p38	Cell Signaling	Cat# 9212; RRID: AB_330713
Mouse monoclonal anti-phospho-SAPK/JNK	Cell Signaling	Cat# 9255; RRID: AB_2307321
Rabbit monoclonal anti-phospho-SAPK/JNK	Cell Signaling	Cat# 4668, RRID: AB_823588
Rabbit monoclonal anti-SAPK/JNK	Cell Signaling	Cat# 9258; RRID: AB_2141027
Rabbit polyclonal anti-ZAK	Proteintech	Cat#14945-1-AP; RRID: AB_1064269
Mouse monoclonal anti-p150	BD biosciences	Cat# 610473, RRID: AB_397845
Mouse monoclonal anti-α-Tubulin	Merck	Cat# T9026, RRID: AB_477593
Mouse monoclonal anti-HA-tag	Santa Cruz Biotechnology	Cat# sc-7392 HRP, RRID: AB_2894930
Mouse monoclonal anti-Puromycin	Millipore	Cat# MABE343, RRID: AB_2566826
Rabbit polyclonal anti-RACK1	Bethyl	Cat# A302-545A, RRID: AB_1999012
Rabbit monoclonal-anti RPS10	Abcam	Cat# ab151550, RRID: AB_2714147
Mouse monoclonal anti-GFP	Roche	Cat# 11814460001, RRID: AB_390913
Mouse monoclonal anti-gamma-tubulin	Sigma	Cat# T5326, RRID: AB_532292
Mouse monoclonal anti-RPL19	Novus Biologicals	Cat# H00006143-M01, RRID: AB_509253
Rabbit polyclonal anti-GCN2	Cell Signaling	Cat# 3302, RRID:AB_2277617
Rabbit polyclonal anti-GCN1L1	ThermoFisher	Cat# A301-843A, RRID:AB_1264319
Rabbit polyclonal anti-GST	Sigma	Cat# G7781, RRID: AB_259965
**Chemicals, peptides, and recombinant proteins**		
Doxycycline	Merck	Cat# D3347
Anisomycin	Merck	Cat# A9789
Puromycin	Cayman Chemical	Cat# 13884
Blasticidin S	Thermo Fisher	Cat# 3513-03-9
Lactimidomycin	Merck	Cat# 5062910001
dTAGV-1	Tocris	Cat# 6914
Torin	InvivoGen	Cat# inh-tor1
Harringtonine	Cayman Chemical	Cat# 15361
AP20718	MedChemExpress	Cat# HY-13992
ZAK inhibitor 6p	Gift from Xiaoyun Lu (Jinan University, China)	N/A
Zeocin	Thermo Fisher	Cat# R25001
Blastidicin	Thermo Fisher	Cat# R21001
CC-885	MedChemExpress	Cat# HY-101488
FUGENE6	Promega	Cat# E2692
Lipofectamine™ 2000	Thermo Fisher	Cat# 11668027
Strep-Tactin Sepharose	IBA Life Sciences	Cat# 10049889
RiboLock RNase Inhibitor	Thermo Fisher	Cat# EO0381
Protease Inhibitor Cocktail	Sigma	Cat# P2714
Micrococcal Nuclease (MNase)	NEB	Cat# M0247
TURBO DNase	Invitrogen	Cat# AM2238

(Continued on next page)

CellPress
OPEN ACCESS

Continued

REAGENT or RESOURCE	SOURCE	IDENTIFIER
RNase I	Invitrogen	Cat# AM2295
RNase I	Biosearch Technologies	Cat# N6901K
SUPERase-In RNase Inhibitor	Invitrogen	Cat# AM2696
T4 Polynucleotide Kinase, Cloned	Biosearch Technologies	Cat# P0503K
T4 RNA Ligase 2, truncated KQ	NEB	Cat# M0373L
5′ Deadenylase	NEB	Cat# M0331S
Rec Jf Exonuclease	NEB	Cat# M0264L
EpiScript™ RNase H- Reverse Transcriptase	Biosearch Technologies	Cat# ERT12925K
Exonuclease I	Biosearch Technologies	Cat# X40520K
Hybridase	Biosearch Technologies	Cat# H39500
Circligase II ssDNA Ligase	Biosearch Technologies	Cat# CL9025K
Phusion® High-Fidelity PCR Master Mix with HF Buffer	NEB	Cat# M0531S
RNA Clean & Concentrator™-5 (200 Preps) w/ Zymo-Spin	Zymo Research	Cat# R1016
Cycloheximide	Sigma-Aldrich	Cat# C7698
Cytiva illustra™ MicroSpin™ S-400 HR Columns	Fisher Scientific	Cat# 27-5140-01
MagStrep Tactin-XT beads	IBA	Cat# 2-5090-010
cOmplete™ protease inhibitor cocktail tablet	Merck	Cat# 11697498001
ZAKα CR2 WT peptide: ^{603}HFDGQDSYAAAVRRPQVPIK^{623}	This paper, JPT Peptide Technologies	N/A
ZAKα CR2 mutant peptide: ^{603}HFDGQDSGGARGREPQVPIK[49]	This paper, JPT Peptide Technologies	N/A
**Critical commercial assays**		
NextSeq™ 1000/2000 P^2 XLEAP-SBS™ Reagent Kit (100 Cycles)	Illumina	Cat# 20100987
NextSeq™ 2000 P3 XLEAP-SBS™ Reagent Kit (100 Cycles)	Illumina	Cat# 20100990
siTools Biotech rRNA depletion kit	siTOOLs	Cat# dp-K096-000101
miRNeasy kit	Qiagen	Cat# 217004
HiFi DNA Assembly Master Mix	NEB	E2621
**Deposited data**		
ZAKα iCLIP2-seq data	This paper	GEO: GSE292064
U2OS anisomycin Ribo-seq data	This paper	GEO: GSE314163
Original Data in Mendeley Data	This paper	10.17632/tr4wzcp7h4.1
**Experimental models: Cell lines**		
Female human osteosarcoma cells (U2OS)	ATCC	HTB-96; RRID: CVCL0042
Female human malignant cervical epithelial cells (HeLa)	ATCC	CCL-2; RRID: CVCL0030
Female human embryonic kidney (HEK293)	ATCC	CRL-1573; RRID: CVCL0045
Female human embryonic kidney (HEK293FT)	ATCC	CRL-3216; RRID:CVCL_0063
Male human near haploid cells (HAP1)	Gift from Thijn Brummelkamp	RRID: CVCLY019
**Oligonucleotides**		
ΔRACK1 cell line: gRNA-RACK1-1-Fw; 5′-CACCGATTCCACAGCGTGCTCTTGCG	Park and Walsh[50]	N/A

(*Continued on next page*)

Continued

REAGENT or RESOURCE	SOURCE	IDENTIFIER
ΔRACK1 cell line: gRNA-RACK1-1-Rv; 5′-AAACCGCAAGAGCACGCTGTGGAATC	Park and Walsh[50]	N/A
ΔGCN1 cell line: gRNA-GCN1-Fw: 5′- AGACACTAAAGCGTTTTGCA	This paper	N/A
ΔGCN1 cell line: gRNA-GCN1-Rv: 5′- TGCAAAACGCTTTAGTGTCT	This paper	N/A
ΔGCN2 cell line: gRNA-GCN2-Fw: 5′-CAAATCCACTTTTACATATA	This paper	N/A
ΔGCN2 cell line: gRNA-GCN2-Rv: 5′-TATATGTAAAAGTGGATTTG	This paper	N/A
RiboSeq primer: 8NI-810: /5Phos/NNN NNN NNA TCG TAG ATC GGA AGA GCA CAC GTC TGA A/3ddC/	McGlincy and Ingolia[51]	N/A
RiboSeq primer: 8NI-811: /5Phos/NNN NNN NNA GCT AAG ATC GGA AGA GCA CAC GTC TGA A/3ddC/	McGlincy and Ingolia[51]	N/A
RiboSeq primer: 8NI-812: /5Phos/NNN NNN NNC GTA AAG ATC GGA AGA GCA CAC GTC TGA A/3ddC/	McGlincy and Ingolia[51]	N/A
RiboSeq primer: 8NI-814: /5Phos/NNN NNN NNG ATC AAG ATC GGA AGA GCA CAC GTC TGA A/3ddC/	McGlincy and Ingolia[51]	N/A
RiboSeq primer: 8NI-815: /5Phos/NNN NNN NNG CAT AAG ATC GGA AGA GCA CAC GTC TGA A/3ddC/	McGlincy and Ingolia[51]	N/A
RiboSeq primer: 8NI-816: /5Phos/NNN NNN NNT AGA CAG ATC GGA AGA GCA CAC GTC TGA A/3ddC/	McGlincy and Ingolia[51]	N/A
RiboSeq primer: NI-802 : /5Phos/RNA GAT CGG AAG AGC GTC GTG TAG GGA AAG AG/iSp18/G TGA CTG GAG TTC AGA CGT GTG CTC	McGlincy and Ingolia[51]	N/A
**Recombinant DNA**		
pSpCas9(BB)-2A-Puro (PX459) V2.0	Ran et al.[52]	Addgene plasmid #62988
pSpCas9(BB)-2A-GFP (PX458)	Ran et al.[52]	Addgene plasmid #48138
pX330-U6-Chimeric_BB-CBh-hSpCas9 (px330)	Cong et al.[53]	Addgene plasmid #42230
pcDNA6/TR	Invitrogen	V102520
pcDNA4/TO/Strep-HA-ZAKα FL (WT)	Vind et al.[24]	Addgene plasmid #141193, RRID: Addgene_141193
pcDNA4/TO/Strep-HA-ZAKα Δ417-422	This paper	N/A
pcDNA4/TO/Strep-HA-ZAKα Δ611-617	This paper	N/A
pcDNA4/TO/Strep-HA-ZAKα Δ417-422 Δ611-617 (ΔRACK1)	This paper	N/A
pcDNA4/TO/Strep-HA-ZAKα R->A	Johansen et al.[33]	N/A
pcDNA4/TO/Strep-HA-ZAKα ΔCTD	Vind et al.[24]	Addgene plasmid # 141196, RRID: Addgene_141196
pcDNA4/TO/Strep-HA-ZAKα R->A ΔCTD	Johansen et al.[33]	N/A
pcDNA4/TO/Strep-HA-ZAKα Δ768-780	This paper	N/A
pcDNA4/TO/Strep-HA-ZAKα 1–713	This paper	N/A
pcDNA4/TO/Strep-HA-ZAKα 1–332	This paper	N/A
pcDNA4/TO/FKBP12-HA-ZAKα FL	This paper	N/A

(*Continued on next page*)

CellPress
OPEN ACCESS

Continued

REAGENT or RESOURCE	SOURCE	IDENTIFIER
pcDNA4/TO/FKBP12-HA-ZAKα 1–332	This paper	N/A
pcDNA4/TO/Strep-HA-ZAKα ΔLZ	This paper	N/A
pcDNA4/TO/Strep-HA-ZAKα ΔYLD	This paper	N/A
pcDNA4/TO/Strep-HA-ZAKα K458Y, W500D	This paper	N/A
pcDNA4/TO/Strep-HA-ZAKα Δ768-772	This paper	N/A
pcDNA4/TO/Strep-HA-ZAKα R->A Δ768-772	This paper	N/A
pcDNA4/TO/Strep-HA-ZAKα Δ670-712	This paper	N/A
pcDNA4/TO/Strep-HA-ZAKα 625–760	This paper	N/A
pCDH-RACK1 WT(untagged)	This paper	N/A
pCDH-RACK1 dAH mutant (untagged)	This paper	N/A
Lentiviral plasmid: psPAX2	Gift from Didier Trono	Addgene plasmid # 12260; RRID: Addgene_12260
Lentiviral plasmid: pCMV-VSV-G	Gift from Bob Weinberg[54]	Addgene plasmid # 8454; RRID: Addgene_8454
Software and algorithms		
ProtParam ExPASy	Wilkins et al.[55]	https://web.expasy.org/protparam/
MicroCal PEAQ-ITC Analysis Software v1.41	Malvern	https://www.malvernpanalytical.com/en/support/product-support/software/microcal-peaq-itc-analysis-software-v141
Python 3	Python Software Foundation	https://www.python.org
Polars v1.30.0	Polars python package	https://pypi.org/project/polars/
Seaborn v0.13.2	N/A	https://pypi.org/project/seaborn/
Matplotlib v3.9.2	N/A	https://pypi.org/project/matplotlib/
Ribominer v0.2	Li et al.[56]	https://github.com/xryanglab/RiboMiner
Plastid v0.6.1	Dunn and Weissman[57]	https://github.com/joshuagryphon/plastid
Bedtools v2.31.0	Quinlan[58]	http://code.google.com/p/bedtools
Samtools v1.22.1	Li et al.[59]	https://samtools.sourceforge.net/
Subread v2.0.6	Liao et al.[60]	https://subread.sourceforge.net/
STAR v2.7.11b	Dobin et al.[49]	https://code.google.com/archive/p/rna-star/
Fastqc v0.11.9	N/A	https://www.bioinformatics.babraham.ac.uk/projects/fastqc/
Trimgalore v 0.6.10	N/A	https://www.bioinformatics.babraham.ac.uk/projects/trim_galore/
Pensa v0.5.0	Vögele et al.[61]	https://github.com/drorlab/pensa
MDAnalysis v2.2.0	Michaud-Agrawal et al.[62]	http://mdanalysis.googlecode.com/
Gromacs v2024.5	N/A	https://gitlab.com/gromacs/gromacs
Pymol v3.1	Schrodinger Foundation	https://www.pymol.org/
ChimeraX v1.10	Meng et al.[63]	https://www.cgl.ucsf.edu/chimerax/download.html
Alphafold3 v3.0.1	Abramson et al.[25]	https://github.com/google-deepmind/alphafold3
Pyfaidx v0.8.1.3	N/A	https://pypi.org/project/pyfaidx/
Code for AF3 prediction mapping on to a.cif structure	This paper	10.5281/zenodo.19709634
Code for crosslink mapping on to a.cif structure	This paper	10.5281/zenodo.19709634

EXPERIMENTAL MODEL AND STUDY PARTICIPANT DETAILS

Cell lines

The following cell lines were used in this study: Female human osteosarcoma cells (U2OS) cells (ATCC, HTB-96; RRID: CVCL0042), female human malignant cervical epithelial cells (HeLa) (ATCC, CCL-2; RRID: CVCL0030), female human embryonic kidney (HEK293) (ATCC, CRL-1573; RRID: CVCL0045) and female HEK293FT (ATCC CRL-3216, RRID:CVCL_0063).

Bacterial strains

The following bacterial strains were used in this study: RosettaTM2 *E. coli* cells (recombinant protein production of human RACK1 for ITC) and competent DH5α *E. coli* cells (plasmid cloning).

METHOD DETAILS

Plasmids

Plasmids containing truncations of ZAKα were PCR-cloned into pcDNA4/TO/Strep-HA using NotI restriction sites and small internal deletions were generated using an overlapping primer-based method.[64] The W500D+K458Y mutant was synthesized by Integrated DNA Technologies (IDT) and subcloned into pcDNA4/TO/Strep-HA using NotI restriction sites. The untagged RACK WT and dAH mutant cDNAs were synthesized and cloned into pcDNA3.1+ by IDT. For generation of pCDH-RACK-BlastR constructs, both RACK1 WT and dAH mutant cDNAs were recloned into pCDHblast-MCSNard OST-LMNAd50 (Addgene #22662), upon restriction digestion with BamHI and EcoRI, via Gibson assembly using the HiFi DNA Assembly Master Mix (NEB). For generation of pcDNA4/TO/FKBP12-HA-ZAKα (FL and 1-332), FKBP12 was cloned into the vector at the expense of the strep-tag.

HAP1 ΔRACK1 cells were previously described.[50] gRNAs for CRIPSR/Cas9-mediated generation of HeLa ΔRACK1 cells were cloned using the pX459 plasmid (Addgene, #62988[52]). In brief, gRNA DNA oligos were ordered as complimentary sequences with overhangs and mixed at a 1:1 ratio for annealing. pX459 was digested with BbsI, and the gRNA was introduced using standard ligation reaction (NEB). The following gRNA sequences were used[65]: RACK1-1-Fw; 5'-CACCGATTCCACAGCGTGCTCTTGCG and RACK1-1-Rv; 5'- AAACCGCAAGAGCACGCTGTGGAATC. All constructs were verified by sequencing, and all plasmid transfections were done using FUGENE6 (Promega, #E2692) according to the manufacturer's protocol.

HAP1 ΔGCN1 and ΔGCN2 cell lines were generated using CRISPR/Cas9 with different selection strategies, respectively. For ΔGCN1, the sgRNA was cloned into the pSpCas9(BB)-2A-GFP (PX458, Addgene #48138[52]), and the px458-gGCN1 plasmid was transfected into HAP1 cells using Lipofectamine 2000 (Thermo Fisher, 11668027) according to the manufacturer's instructions. 24h post-transfection, single GFP-positive cells were sorted into 96-well plates by FACS (BD FACSAria™ Fusion) for clonal outgrowth. For ΔGCN2, the sgRNA was cloned into pX330-U6-Chimeric_BB-CBh-hSpCas9 (px330, Addgene #42230[53]), and the px330-gGCN2 plasmid was co-transfected with a blasticidin resistance cassette plasmid (10:1 ratio) using Lipofectamine 2000. 24h post-transfection, cells were selected with 30 μg/ml blasticidin S HCl (Thermo Fisher, A1113902) for 48 h. Surviving cells were expanded and single-cell clones were isolated by limiting dilution. ΔGCN1 and ΔGCN2 clones were validated by immunoblotting. Ploidy of KO cell lines was assessed by flow cytometry and all clones confirmed to be haploid. The sgRNA sequences used were: sgGCN1-Fw: 5'- AGACACTAAAGCGTTTTGCA, sgGCN1-Rev: 5'- TGCAAAACGCTTTAGTGTCT, sgGCN2-Fw: 5'-CAAATC CACTTTTACATATA, sgGCN2-Rev: 5'- TATATGTAAAAGTGGATTTG.

Cell culture and reagents

Female human osteosarcoma cells (U2OS) cells (ATCC, HTB-96; RRID: CVCL0042), female human malignant cervical epithelial cells (HeLa) (ATCC, CCL-2; RRID: CVCL0030), female human embryonic kidney (HEK293) (ATCC, CRL-1573; RRID: CVCL0045) and female HEK293FT (ATCC, CRL-3216; RRID:CVCL_0063) were cultured in Dulbecco's Modified Eagle's Medium (DMEM, Biowest # L0104-500) supplemented with a 10% fetal bovine serum, penicillin and streptomycin. Male human near haploid cells (HAP1) (RRID: CVCLY019) were cultured in Iscove's Modified Dulbecco's Medium (IMDM) GlutaMAX™ Supplement (Gibco # 31980030) supplemented with 10% FBS, penicillin, and streptomycin. All cells were cultured at 37°C in a humidified 5-8% CO2 cell incubator. Derivative cell lines U2OS/ΔZAK, U2OS/ΔZAK/strep-HA-ZAKα_WT, U2OS/ΔZAK/strep-HA-ZAKα_R/K→A, U2OS/ΔZAK/strep-HA-ZAKα_ΔCTD, U2OS/ΔZAK/strep-HA-ZAKα_Δ670-712, U2OSΔZAK_R/K→A ΔCTD have been previously described.[24,33] HAP1/ΔRACK1 and HAP1/ΔRACK1/FLAG_RACK1 cells were a gift from Eric Bennett. To generate cell lines stably expressing truncations, internal deletions and point mutants of ZAKα under doxycycline inducible promoters, cells were co-transfected with pcDNA4/TO/Strep-HA-ZAKα constructs and pcDNA6/TR (Invitrogen, #V102520) in a 1:4 ratio and selected for 14 days with zeocin (200 μg/ml) and blasticidin (5 μg/ml). Individual clones were picked, and expression analysed by immunofluorescence and Western blotting. Unless otherwise indicated in figure legends, all U2OS/ΔZAK rescue cell lines were treated with doxycycline overnight to induce expression of the transgene. Chemicals and inhibitors used in this study were: Doxycycline (Merck, #D3347, 0.13 μg/ml, overnight), anisomycin (Merck, A9789), puromycin (Cayman Chemical, #13884), lactimidomycin (Merck, #5062910001), AP20718 (MedChemExpress #HY-13992), CC-885 (MedChemExpress, #HY-101488), harringtonine (Cayman Chemicals, #15361) ZAK inhibitor 6p (a gift from Xiaoyun Lu), zeocin (Thermo Fisher, #R25001) and blastidicin (Thermo Fisher, # R21001).

Lentivirus production and transduction

For lentivirus production, HEK 293FT cells were cultured in DMEM supplemented with a 10% fetal bovine serum. After 24h, cells were co-transfected with pCDH-BlastR lentiviral plasmid (expressing wt and dAH mutant forms of RACK1), pCMV-VSV-G (Addgene #8454[54]), and psPAX2 (Addgene #12260), using polyethylenimine (PEI). Supernatants containing lentiviral particles were collected 48h after transfection and filtered through a 0.45 μm PES filter. HAP1 RACK KO cells were transduced by addition of viral supernatant and 10 μg/ml polybrene, following antibiotic selection with blasticidin (5 μg/ml).

Phosphatase treatment

Cells were lysed in EBC buffer without EDTA and phosphatase inhibitors and $MnCl_2$ was added to a final concentration of 1 mM. 400 U of lambda phosphatase (NEB) were added or not, and the extracts were incubated for 30 min at 30 °C, directly mixed with Laemmli sample buffer, and boiled for 5 min before western blotting.

Western blotting, pull-down and antibodies

For whole-cell extracts, cells were lysed in EBC buffer (50 mM Tris, pH 7.5, 150 mM NaCl, 1 mM EDTA, 0.5% NP-40, protease and phosphatase inhibitors), mixed with Laemmli sample buffer and boiled for 5-10 min. Pull-downs were done with Strep-Tactin Sepharose (IBA Life Sciences, #2-1201-010). Protein samples were resolved by SDS-PAGE and transferred to nitrocellulose membranes. Membranes were blocked in PBS-T + 5% milk before incubation with primary antibody overnight at 4 °C. Membranes were then washed in PBS-T and incubated with secondary antibody for 1 h at room temperature, before being washed in PBS-T and visualized by chemiluminescence (Clarity Western ECL substrate, Bio-Rad) using the Bio-Rad Chemidoc imaging system. Antibodies used: Rabbit polyclonal anti-ZAKα (Bethyl, Cat#A301-993A; RRID: AB_1576612), Mouse monoclonal anti-phospho-p38 (Cell Signaling, Cat#9216; RRID: AB_331296) Rabbit monoclonal anti-phospho-p38 (Cell Signaling, Cat#4511S; RRID: AB_2139682), Rabbit polyclonal antibody anti-p38 (Cell Signaling, Cat#9212; RRID: AB_330713), Mouse monoclonal anti-phospho-SAPK/JNK (Cell Signaling, Cat#9255; RRID: AB_2307321), Rabbit monoclonal anti-phospho-SAPK/JNK (Cell Signaling, Cat#4668, RRID:AB_823588), Rabbit monoclonal anti-SAPK/JNK (Cell Signaling, Cat#9258; RRID: AB_2141027), Rabbit polyclonal anti-ZAK (Proteintech, Cat#14945-1-AP; RRID: AB_1064269), Mouse monoclonal anti-p150 (BD biosciences, Cat#610473; RRID: AB_397845), Mouse monoclonal anti-α-Tubulin (Merck, Cat#T9026; RRID: AB_477593), Mouse monoclonal anti-HA-tag (Santa Cruz Biotechnology, Cat#sc-7392 HRP; RRID: AB_2894930), Mouse monoclonal anti-Puromycin (Millipore, Cat#MABE343; RRID: AB_2566826), Rabbit polyclonal anti-RACK1 (Bethyl, Cat#A302-545A; RRID: AB_1999012), Rabbit monoclonal anti-RPS10 (Abcam # ab151550; RRID: AB_2714147), Rabbit polyclonal anti-GST (Sigma, Cat#G7781; RRID: AB_259965), Rabbit polyclonal anti-GCN1 (Thermo Fisher, A301-843A; RRID: AB_1264319), Rabbit polyclonal anti-GCN2 (Cell Signaling, Cat#3302S; RRID: AB_2277617), Mouse monoclonal anti-GFP (Roche Cat# 11814460001; RRID:AB_390913), Mouse monoclonal anti-gamma-tubulin (Sigma, #T5326; RRID:AB_532292), Mouse monoclonal anti-RPL19 (Novus Biologicals #H00006143-M01; RRID:AB_509253).

Sucrose cushions

Crude cellular ribosome pellets were generated by lysing cells in lysis buffer (15 mM Tris, pH 7.5, 0.5% NP40, 6 mM $MgCl_2$, 300 mM NaCl, RiboLock RNase inhibitor (Thermo Fisher Scientific, #E00381)) and clearing the lysate at 12,000 g, 4 °C, 10 min. The supernatant was carefully layered onto a sucrose cushion (30% sucrose in 20 mM Tris, pH 7.5, 2 mM $MgCl_2$, 150 mM KCl) and ultra-centrifuged at 38,800 rpm for 16 h using a Sorvall wX+ Ultrafuge and a FIBERlite F50L-8x39 rotor. Supernatants were discarded and pellets were washed in PBS and re-suspended (100 mM KCl, 5 mM $MgCl_2$, 20 mM HEPES, pH 7.6, 1 mM DTT and 10 mM NH_4Cl). Pellets and whole cell extracts were boiled in Laemmli buffer and analyzed by Western Blot.

Ribo-seq libraries

U2OS WT cells were treated for 15 min with DMSO or with 0.19 μM or 76 μM anisomycin (ANS). After incubation, cells were harvested in cold PBS + 100 μg/ml CHX cycloheximide (CHX, Sigma, #C7698) + 100 μg/ml ANS. Lysis of the cells was performed in the buffer described in Janich et al.,[66] complemented with 100 μg/ml ANS. RPFs (ribosome-protected fragments) were then generated by RNase I (Invitrogen, # AM2295, 43 units/OD) and Turbo DNase (Invitrogen, #AM2238, 0.3 units/OD) digestion of the extracts. Digestion was stopped by the addition of 8.7 μl of SUPERase-In (Invitrogen, #AM2696), and RPFs were purified on pre-washed S-400 HR columns (Cytiva, #27-5140-01) before their extraction (Qiagen - miRNeasy kit, #217004). 2.5 μg of RPFs were migrated and size-selected on a 15% TBE–urea gel. As described and adapted from McGlincy and Ingolia,[51] RNA was repaired at the 3′ end with PNK (Biosearch Technologies, #P0503K). The 5′-adenylated adaptors containing a barcode and an 8N UMI were then ligated using T4 RNA Ligase 2 Deletion Mutant (NEB, #M0373L). Samples were multiplexed, and adaptor removal was performed for 1 h at 30 °C and 1 h at 37 °C by treating libraries with 2 μl of a 1:1 mix of 5′ deadenylase (NEB, #M0331S) and RecJf (NEB, #M0264L). Ribosomal RNA was depleted according to the siTOOLs Biotech rRNA depletion kit (siTOOLs, #dp-K096-000101) specifications, with clean-up steps performed using Zymo Clean & Concentrator columns (Zymo Research, #R1016). Further library preparation steps were carried out as described.[51] Briefly, libraries were reverse transcribed using EpiScript (Biosearch Technologies, #ERT12925K) and cleared by sequential treatment with Exonuclease I (Biosearch Technologies, #X40520K) and a 1:1 mix of RNase I and Hybridase (Biosearch Technologies, #H39500). After circularization of the libraries with CircLigase II (Biosearch Technologies, #CL9025K),

PCR amplification (Phusion polymerase – NEB, #M0531S) was carried out using i5 (Nextera D503 or Nextera D504) and i7 (#iA_03 or #iA_04) primers. The libraries were single-read sequenced on an Aviti sequencer.

Ribo-seq data processing

Reads were trimmed using TrimGalore (v. 0.6.10; auto-detect adapter type and minimum length=30nt) and UMIs were extracted from each read with UMItools (v. 1.0.0git; parameters: –extract-method string –bc-pattern NNNNNNNNCCCCC –3prime –filter-cell-barcode –error-correct-cell). Remaining reads were then size-filtered (26-35nt), quality-filtered using fastq_quality_filter (fastx_toolkit v. 0.0.14) and trimmed for 2 nt in the read's 5'-end.

Sequential mapping was performed with STAR (v. 2.7.11b) on rRNA and tRNA (Homo sapiens GRCh38.111; parameters: –seedSearchStartLmax 28 –outSAMmultNmax 1 –outSAMtype BAM SortedByCoordinate Unsorted –outReadsUnmapped Fastx). Unmapped reads were then demultiplexed (split by barcode using demuxbyname.sh from BBTools) and mapped to the genome (parameters: –outSAMunmapped Within –outFilterType BySJout –outSAMattributes NH HI AS NM MD –outFilterMultimapNmax 20 –outFilterMismatchNmax 999 –alignIntronMin 20 –alignIntronMax 1000000 –alignMatesGapMax 1000000 –alignSJoverhangMin 8 –alignSJDBoverhangMin 1 –sjdbScore 1 –genomeLoad NoSharedMemory –outSAMtype BAM Unsorted –quantMode TranscriptomeSAM).

Genome-aligned reads were then projected to the transcriptome and filtered to keep only the reads mapped to the single most expressed transcript per gene. Metagene plots were generated from a predicted A-site counts table (offset: +15 nt from read 5'-end) normalized by library depth. Relative expression was then calculated for each transcript. Read density position was expressed as a percentage of the transcript features (5' UTR, CDS and 3' UTR – Figure 4D) or relative to start and stop codons (Figure 4E).

Polysome profiling

Cells were exposed to 500 J/m^2 UVB, different anisomycin concentrations, or nothing (mock) as indicated in the figure legends. Following treatment, cytosolic lysates were prepared using 20 mM Hepes pH 7.5, 100 mM NaCl, 5 mM $MgCl_2$, 100 μg/ml digitonin, 100 μg/ml cycloheximide, 1X protease inhibitor cocktail (Sigma, #P2714) and 200 U RiboLock RNase Inhibitor (Thermo Fisher Scientific, #EO0381).[31] Extracts were pushed 10 times through a 26G needle and incubated on ice for 5 min prior to centrifugation at 17,000 g for 5 min at 4 °C. After adding $CaCl_2$ to a final concentration of 1 mM, lysates were optionally digested with 500 U micrococcal nuclease (MNase) (NEB, #M0247) for 30 min at 22 °C. Digestion was terminated by adding 2 mM EGTA. Equivalent amounts of lysate (180 μg of undigested RNA or 200 μg of MNase-digested RNA) were resolved on 15-50% sucrose gradients by centrifugation at 38,000 rpm in a Sorvall TH64.1 rotor for 2.5 h at 4 °C. The gradients were analyzed using a Biocomp density gradient fractionation system with continuous monitoring of the absorbance at 260 nm.

Protein expression and purification

Purification of the last 100 amino acids of ZAKα was described previously.[24] The plasmid for recombinant expression of *H. sapiens* RACK1 was kindly donated by the Lescar group.[67] Human RACK1 was expressed in Rosetta^{TM2} *E. coli* cells (NEB). Cells were grown in Terrific Broth at 37 °C to an OD600 of ~1, and expression was induced overnight at 18 °C by addition of 1 mM isopropyl β-D-1-thiogalactopyranoside. Protein purification was adapted from previous reports.[29] Cell pellets were resuspended in 50 mM Tris base pH 8, 300 mM NaCl, 10 mM imidazole, 5% glycerol, one cOmplete™ protease inhibitor cocktail tablet (Merck) per 50 mL of buffer, 2 mM phenylmethylsulfonyl fluoride, 0.01 mg/ml DNAse I (Sigma), and 200 μg/ml lysozyme (Sigma). Cells were lysed by sonication (Fisherbrand) on ice, and cleared by centrifugation at 38000 *g* at 4 °C. The protein was first purified on a Ni-immobilised affinity chromatography on a 5 ml His-Trap FF affinity column (Cytiva) equilibrated in 50 mM Tris base pH 8, 300 mM NaCl, 10 mM imidazole, 5% glycerol, and eluted with a shallow linear imidazole gradient (10–500 mM). RACK1 was further purified through a 5 ml HiTrap Heparin HP affinity column (Cytiva) in 50 mM Tris base pH 7.5, 100 mM KCl, 0.2 mM EDTA, 1 mM DTT, 10% glycerol, and eluting with a linear gradient of 0.1–2 M KCl. Fractions containing RACK1 were pooled and buffer-exchanged into 20 mM HEPES pH 7.5, 300 mM NaCl, 10% glycerol, 1 mM DTT, using Vivaspin centrifugal concentrators (Sartorius) with a 30 kDa cut-off.

Protein concentration was determined from absorbance at 280 nm using the theoretical extinction coefficient calculated by ProtParam ExPASy.[55] Purity was assessed by SDS-PAGE. Samples were flash frozen with liquid nitrogen and stored at -80 °C for use in further experiments.

Isothermal titration calorimetry (ITC)

The peptides spanning the putative short, conserved region-2 (CR2) of ZAKα (CR2 WT—^{603}HFDGQDSYAAAVRRPQVPIK^{623}) and its mutant (CR2 mut—^{603}HFDGQDSGGARGREPQVPIK^{623}) were purchased from JPT Peptide Technologies. The lyophilised peptides were resuspended in MilliQ water to final concentrations ranging from 10 to 12 mM. ITC experiments were performed on a PEAQ-ITC microcalorimeter (Malvern Panalytical) in 20 mM HEPES pH 7.5, 300 mM NaCl, 10% glycerol, and 1 mM DTT (ITC buffer). The RACK1 protein and the peptides were diluted to the desired concentration in the ITC buffer. The ZAKα peptides at a concentration of 300 μM were injected into solution of human RACK1 at a concentration of 30 μM. The experiments were conducted at 25 °C following standard procedures. Briefly, each ITC experiment consisted of nineteen 2-μl injections of 2 seconds each, with a spacing of 125 seconds. The instrument was set up to high-feedback mode, reference power 5 μcal/sec, and stirring speed of 700 rpm, and a

60-seconds pre-injection delay was applied for baseline stabilization after equilibration. Heat peaks were integrated and data fitted using the MicroCal PEAQ-ITC Analysis Software (Malvern). ΔH (reaction enthalpy change in kcal/mol), K_D (dissociation constant), and N (molar ratio between the two components of the interaction) were the fitting parameters. The reaction entropy was calculated using the equations $\Delta G = -RT \ln 1/K_D$ and $\Delta G = \Delta H\text{-}T\Delta S$. Experiments were repeated at least in three times. Buffer-into-buffer and peptides-into-buffer control experiments were also performed (not shown).

UV crosslinking, extraction, and immunoprecipitation of crosslinked ZAKα -RNA complexes

Cell lysate preparation and immunoprecipitation (IP) were performed as described in [68] with minor modifications from.[69] In brief, U2OS cells at 80% confluency were subjected to 254 nm (UVC) irradiation at a dose of 150 mJ/cm^2 using a STRATALINKER2000. Two 15 cm plates were used per IP, and experiments were run and sequenced in duplicate for each condition. Cells were harvested, lysed, and sonicated using Branson Digital Sonifier. Whole-cell extracts were treated with TURBO DNAse (Invitrogen, #AM2238) and RNAse I (Invitrogen, # AM2295, 1:500 dilution) prior to IP using MagStrep Tactin-XT beads (IBA, # 2-5090-010). Protein-RNA complexes were subjected to high salt washes, including freshly added 2M Urea in the wash buffer, L3-App linkers were then ligated to 3' ends of co-immunoprecipitated RNA, followed by ^{32}P radiolabelling. To prevent UV-induced autophosphorylation and degradation of ZAKα, the ZAK inhibitor-6p was added at a final concentration of 10 μM in the lysis buffer and 25 μM in the hot PNK reaction mixture during the radiolabelling step. After separation by PAGE electrophoresis and wet transferring to a nitrocellulose membrane, radiolabeled ZAK-RNA complexes were then excised from the membranes and digested with proteinase K to remove ZAKα. RNA was subsequently precipitated by following the protocol from.[68]

Electrophoretic mobility shift assay (EMSA)

For EMSA experiments, 4% native polyacrylamide gels were run in 0.25 x TBE buffer. Samples were prepared in a total volume of 20 μl shift buffer (20 mM HEPES pH 7.6, 3 mM $MgCl_2$, 40 mM KCl, 5 % (v/v) glycerol, 2 mM DTT). 100 fmol of IRDye-labelled RNA probe was added to the purified proteins and incubated for 20 min on ice. Samples were then mixed with 5 x loading buffer (20 % (v/v) glycerol; brome phenol blue) and run for approximately 55 min at 80 V. Gels were pre-run for a minimum of 15 min at 70 V for equilibration. For super-shift experiments, RNA:protein complexes were pre-incubated for 10 min on ice before adding 2.5 μg of GST antibody. This was followed by another 15 min on ice before mixing with loading buffer. Gels were scanned using an Odyssey CLx imaging system (LI-COR). The following 5'-IRDye-labelled RNA probe was used: ARE-IRDye700: 5'- AGCUUAGGAAUAUCAAUGUU AAGUAG-3'. Bacterial 16S/23S rRNA (Roche #10206938001) was used in competition assays.

iCLIP library preparation and sequencing

Libraries were prepared as described in [68] using barcoded adapters and pooled at equimolar concentrations prior to sequencing on an Illumina NextSeq 2000 platform. Sequencing was performed in three batches. In batches 1 and 2, libraries were pooled and applied to a NextSeq™ 2000 P2 XLEAP-SBS™ Reagent Kit (100 Cycles) (Illumina Cat. No. 20100987). In batch three, libraries were pooled and sequenced using a NextSeq™ 2000 P3 XLEAP-SBS™ Reagent Kit (100 Cycles) (Illumina Cat. No. 20100990). Demultiplexing, adapter trimming, quality filtering and PCR-duplicate removal were performed as previously described.[70] Most experimental conditions in each batch were performed in duplicate, downstream analysis and visualization were conducted using the replicate, for each condition, that harbored the most mapped reads after alignment.

Alignment to reference genome and crosslink mapping

Genome assembly (FASTA) and annotation files (GTF, GFF3) were obtained from the NCBI RefSeq human genome assembly (GCF_000001405.40). Quality-filtered reads were aligned to the using the STAR[49] aligner with the parameters described in [70] except for –outFilterMultimapNmax 999 to allow multimapping reads. After alignment, the resulting.bam files were further processed with BEDTools[58] as described in [70] to obtain.bed files harboring the observed number of crosslinks at every genomic location. These.bed files were then converted to.bedgraph format (command bedtools genomecov -bg …) for genome track visualization and to per-base coverage tables (command bedtools genomecov -dz …) for individual transcript visualization. Lastly, from each multimapping-permissive STAR alignment, we generated a corresponding non-multimapping version containing only primary read alignments using Samtools view[59] with parameters -F 0x100 -F 0x800 to filter out secondary and supplementary read alignments.

Crosslinked features quantification

Crosslinked feature quantification was performed on the multimapping-permisive alignments using the featureCounts function from Subread[60] with respect to the relevant RefSeq annotation. The parameters –-readExtension5 1 –-read2pos 5 –-primary were set in all featureCounts calls to count only the crosslinked nucleotide site and avoid counting multimapping reads multiple times.

For mRNA/rRNA/tRNA/other transcript biotype quantification, the RefSeq GTF annotation served as a reference. For UTR/CDS quantification, 3' UTR, 5' UTR, and CDS regions were first extracted from the RefSeq GFF3 annotation file and saved onto a new GFF3 file. Then, the GFF3 with the extracted regions was used as annotation for featureCounts.

Ribosomal RNA crosslinking visualization

For any rRNA gene, track visualization of rRNA crosslinks was achieved by obtaining the genomic coordinates for the location of the given gene (RNA18SN1, RNA5S1, RNA5-8SN1, or RNA28SN1) from the RefSeq annotation and extracting the crosslinks at those coordinates from the per-base coverage tables. Track normalization (conversion from Number of crosslinks per position to Number of crosslinks per million reads per position) was done individually for each aligned iCLIP library by dividing the number of crosslinks at every position in the per-base coverage table by the total number of mapped reads for that library and then multiplying by 10^6. All these operations were performed in Python using Polars, and visualizations were done by Seaborn/Matplotlib.

Metagene profiles of whole transcripts

The package Ribominer was used to calculate transcript-wide metagene profiles as detailed in the Ribominer repository.[56] Briefly, the RefSeq GTF was preprocessed using the module's *prepare_transcripts* function. The output of this step was then fed into the *OutputTranscriptInfo* function to extract all transcripts, and the longest transcripts per gene were extracted from those via a Python script to use as regions of interest (ROIs) for the metagene profiles.

Next, the Ribominer module MetageneAnalysisForTheWholeRegions was used to generate the metagene profiles from the extracted transcripts using as input the non-multimapping alignments, allowing reads of any length and setting an offset of -1. For each condition, transcripts with CDS regions harboring less than 100 codons or less than 10 crosslinks were excluded from the analysis (parameters -l 100 -n 10). The resulting profiles were then graphed using Polars, Seaborn and Matplotlib in Python.

Metagene profiles around start and stop codons

First, ROIs were generated from the RefSeq GTF annotation using Plastid[57] (command metagene generate -–landmark [cds_start|cds_stop] …). ROIs consisted of 100 nt upstream to 400 nt downstream from the start codon or 400 nt upstream to 100 nt downstream from the stop codon, in mRNA coordinates.

Metagene profiles were calculated for both Plastid ROI groups (start and stop) from each non-multimapping alignment (plastid command metagene count), counting only the 5'-most position in each read (–fiveprime) with an offset of 0 (–offset 0), excluding transcripts with less than 20 crosslinks in total in the whole region (–min_counts 20), and normalizing the reads per ROI by the total number of reads in the whole ROI (–normalize_over -100 400 for start ROIs, (–normalize_over -400 100 for stop ROIs). Results were plotted in Python using Polars, Seaborn and Matplotlib.

Visualization of Alphafold scores and iCLIP values in ChimeraX

Alphafold 3 contact probability scores were calculated, for each possible interactor reference sequence, from the output contact probability matrices C_n for the 5 predicted models 0 to 4. First, a consensus contact probability matrix ($CP_{i,j}$) was obtained by

$$CP_{i,j} = \max_{i,j}\left(C_{n \in (0,1,2,3,4)}\right)$$

Then, an array representing the maximum contact probability (iCP) with chain A (ZAKα) for each residue in chain B (possible interactor) was defined as

$$iCP = \max_{i}\left[\operatorname*{mean}_{i \in A, j \in B}\left(CP_{i,j}, CP_{i,j}{}^{T}\right)\right]$$

A corresponding reference sequence and array for the number of crosslinks per nucleotide was attained from the iCLIP data for the rRNA transcripts. Upon obtaining the genomic coordinates for a given rRNA gene, its nucleotide sequence was extracted from the reference genome file, and crosslinks were obtained from the per-base coverage tables. This allowed the assignment of individual crosslink values to each individual base in the nucleotide sequence of any given ribosomal transcript. These residue- and nucleotide-resolution values were then mapped from the reference protein (from AF3 predictions) and nucleotide (from genomic coordinates) sequences to those present in the target ribosome structure (4UG0)[27] by sequence alignment. First, each sequence in the ribosome structure was assigned to the best aligning reference sequence. Then, iCP or number of crosslinks were transferred from reference to structure residues/nucleotides directly following the alignment, by mapping iCP or crosslink values from each reference residue/nucleotide to its corresponding aligned residue/nucleotide in the ribosome structure. The resulting structural residue-value pairs and structural nucleotide-value pairs were then used to write a ChimeraX[63] attribute file for visualization.

Molecular dynamics simulation

Molecular dynamics simulations were run using GROMACS 2024.5[71] in the CHARMM36[72] force field. Simulation solvent consisted of 150 mM NaCl in water, plus any extra ions to reach net neutrality in the solution. The simulation pipeline consisted of an energy minimization step, followed by 5 ns NPT and 5 ns NVT equilibration, and finally a 100 ns production run, with 2 fs timesteps and a constant temperature of 310 K. RACK1 residues in direct contact with other ribosomal components (as per 4UG0) were restrained during production runs. We first ran the top ranked ZAKα-RACK1 Alphafold 3 structure once through the pipeline and used the output topology as input for all subsequent simulations. Both RACK1-present and -absent simulations were run in triplicates, generating random velocities for the atoms of each replicate once at the start of each NPT step. The initial topology for the RACK1-absent simulations was

obtained by removing RACK1 from the input ZAKα-RACK1 and re-solvating ZAKα before starting the pipeline. Finally, simulation data was analysed using the Numpy[73], PENSA[61] (JSD calculation), and MDAnalysis[62] (inter-domain distance calculation) Python packages, while movies and superimpositions were composed in Pymol v3.1.

QUANTIFICATION AND STATISTICAL ANALYSIS

All statistical analyses were performed in Python and the statistical details can be found in the figure legends and in the Method details.

Molecular Cell, Volume *86*

Supplemental information

Mechanism of RACK1-dependent ZAKα activation at stalled and collided ribosomes

Anna Constance Vind, José Francisco Martínez, Zhenzhen Wu, Andrii Bugai, Kelly Mordente, Giancarlo Abis, Sébastien Chamois, Sofia Ramalho, Catarina Pechincha, Laura Ryder, Qiuyan Chen, Mads Rasmussen, Xinyao Shi, Dandan He, Jesper Q. Svejstrup, Peter Haahr, David Gatfield, Maria R. Conte, Torben Heick Jensen, Melanie Blasius, and Simon Bekker-Jensen

Vind et al, Figure S1

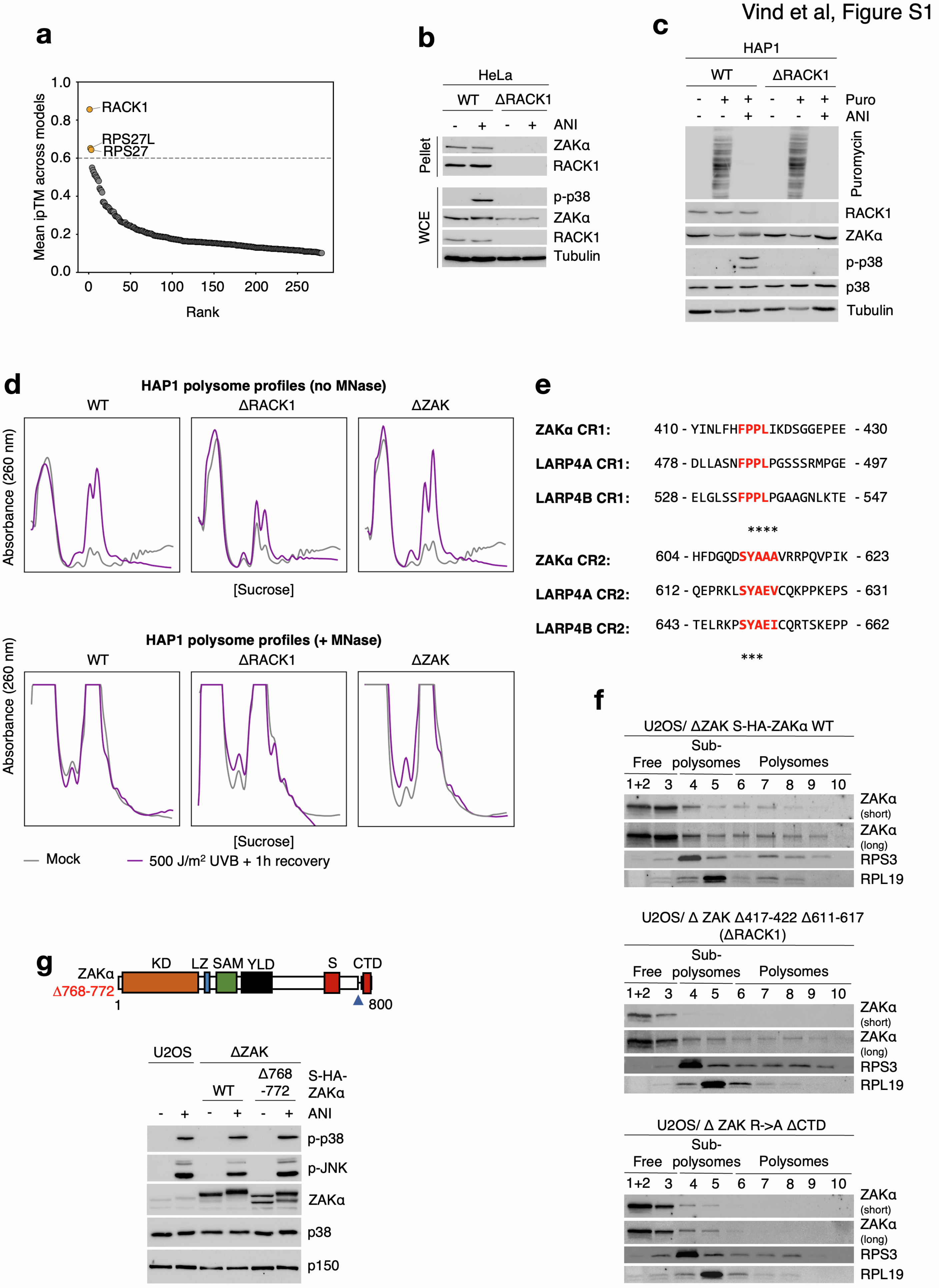

Figure S1.

RACK1 is required for ZAKα-ribosome interaction, related to Figure 1 and Figure 2

a. Alphafold3 (AF3) prediction scores for ZAKα binding to proteins in the Reactome (reactome.org) pathway "Translation", R-HSA-72766. Predictions are ranked according to ipTM score and dashed line indicates our cutoff score of ipTM = 0.6. **b.** Hela WT and ΔRACK1 cells were treated with ani (1 μM - 1 h) and lysates were ultracentrifuged through sucrose cushions. Whole cell extract (WCE) and pelleted material (pellet) containing ribosomes were analyzed by immunoblotting with the indicated antibodies. **c.** HAP1 WT and ΔRACK1 cells were treated with anisomycin (Ani - 0.5 μg/ml, 1 hour). Puromycin (10 μg/ml) was added to the culture 10 min prior to harvest and lysates were analyzed by immunoblotting with the indicated antibodies. **d.** HAP1 WT, ΔRACK1 and ΔZAK cells were irradiated with UVB (500 J/m^2 - 1 hour). Lysates were treated (bottom) or not (top) with MNase to convert polysomes to monosomes. Materials were separated on a linear sucrose gradient and UV absorbance was measured with a fraction collector to indicate RNA (ribosome) content. **e.** Alignment of ZAKα short linear interaction motifs (SLIMs) binding to RACK1 with motifs in LARP4A and LARP4B (CR1 and CR2) previously shown to occupy the same binding sites on RACK1. Note the high identity / similarity between residues that directly contact RACK1. **f.** Polysomal material from U2OS / ΔZAK cells stably rescued with WT and mutated forms of strep-HA-tagged ZAKα were separated by sucrose gradient centrifugation. Proteins in collected fractions were precipitated and analyzed by immunoblotting with the indicated antibodies. **g.** U2OS / ΔZAK cells stably rescued with WT and an RPS27 binding-deficient mutant of strep-HA-tagged ZAKα were treated as in (b) and analyzed by immunoblotting with the indicated antibodies.

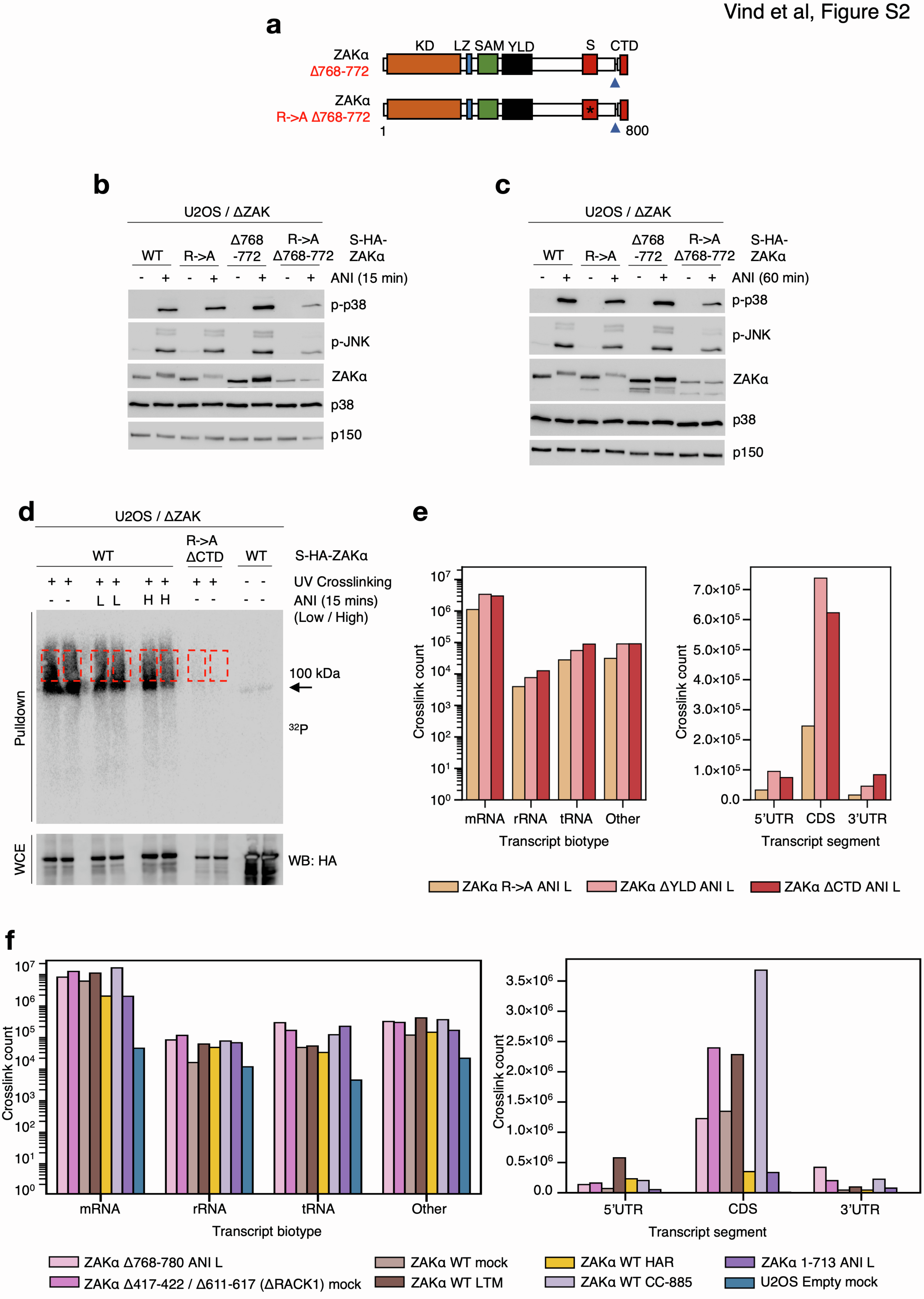
a
ZAKα
Δ768-772
ZAKα
R->A Δ768-772
KD
LZ
SAM
YLD
S
CTD
1
800
b
U2OS / ΔZAK
WT
R->A
Δ768 -772
R->A Δ768-772
S-HA-ZAKα
ANI (15 min)
p-p38
p-JNK
ZAKα
p38
p150
c
U2OS / ΔZAK
WT
R->A
Δ768 -772
R->A Δ768-772
S-HA-ZAKα
ANI (60 min)
p-p38
p-JNK
ZAKα
p38
p150
d
U2OS / ΔZAK
WT
R->A ΔCTD
WT
S-HA-ZAKα
UV Crosslinking
ANI (15 mins) (Low / High)
Pulldown
100 kDa
32P
WCE
WB: HA
e
Crosslink count
mRNA
rRNA
tRNA
Other
Transcript biotype
5'UTR
CDS
3'UTR
Transcript segment
ZAKα R->A ANI L
ZAKα ΔYLD ANI L
ZAKα ΔCTD ANI L
f
Crosslink count
mRNA
rRNA
tRNA
Other
Transcript biotype
5'UTR
CDS
3'UTR
Transcript segment
ZAKα Δ768-780 ANI L
ZAKα Δ417-422 / Δ611-617 (ΔRACK1) mock
ZAKα WT mock
ZAKα WT LTM
ZAKα WT HAR
ZAKα WT CC-885
ZAKα 1-713 ANI L
U2OS Empty mock

Figure S2.

RPS27 binding is dispensable for ZAKα activation, related to Figure 2

a. Schematics of ZAKα mutants deficient for RPS27 binding (Δ768-772) with and without (R->A) S domain functionality. **b.** U2OS / ΔZAK cells stably rescued with strep-HA-tagged ZAKα mutants from (a) were treated with anisomycin (ani – 1 µM, 15 min). Lysates were analyzed by immunoblotting with the indicated antibodies. **c.** As in (b), except that cells were treated with ani for 60 min. **d.** U2OS / ΔZAK cells stably rescued with WT and ribosome-binding deficient (R->A ΔCTD) forms of strep-HA-tagged ZAKα were treated with ZAK inhibitor (10 µM, 30 min) and anisomycin (ani L – 0.19 µM; Ani H - 76 µM) for 15 min as indicated. Cells were crosslinked by UVC irradiation (150 mJ/cm^2) and lysates were treated with DNase and RNase and subjected to strep purification. RNA 3' ends were ligated to L3-App linkers and radiolabelled with ^{32}P. Material was separated by PAGE and developed by autoradiography. Red boxes indicate the areas of the gel that were excised and processed for library preparation and sequencing. **e.** Left: Total number of ZAKα crosslinks from samples of sequencing batch 2 from Fig. 2f according to RNA category. Right: As in (left), except according to mRNA elements. **f.** As in (e), except for crosslinks from batch 3 from Fig. 2f.

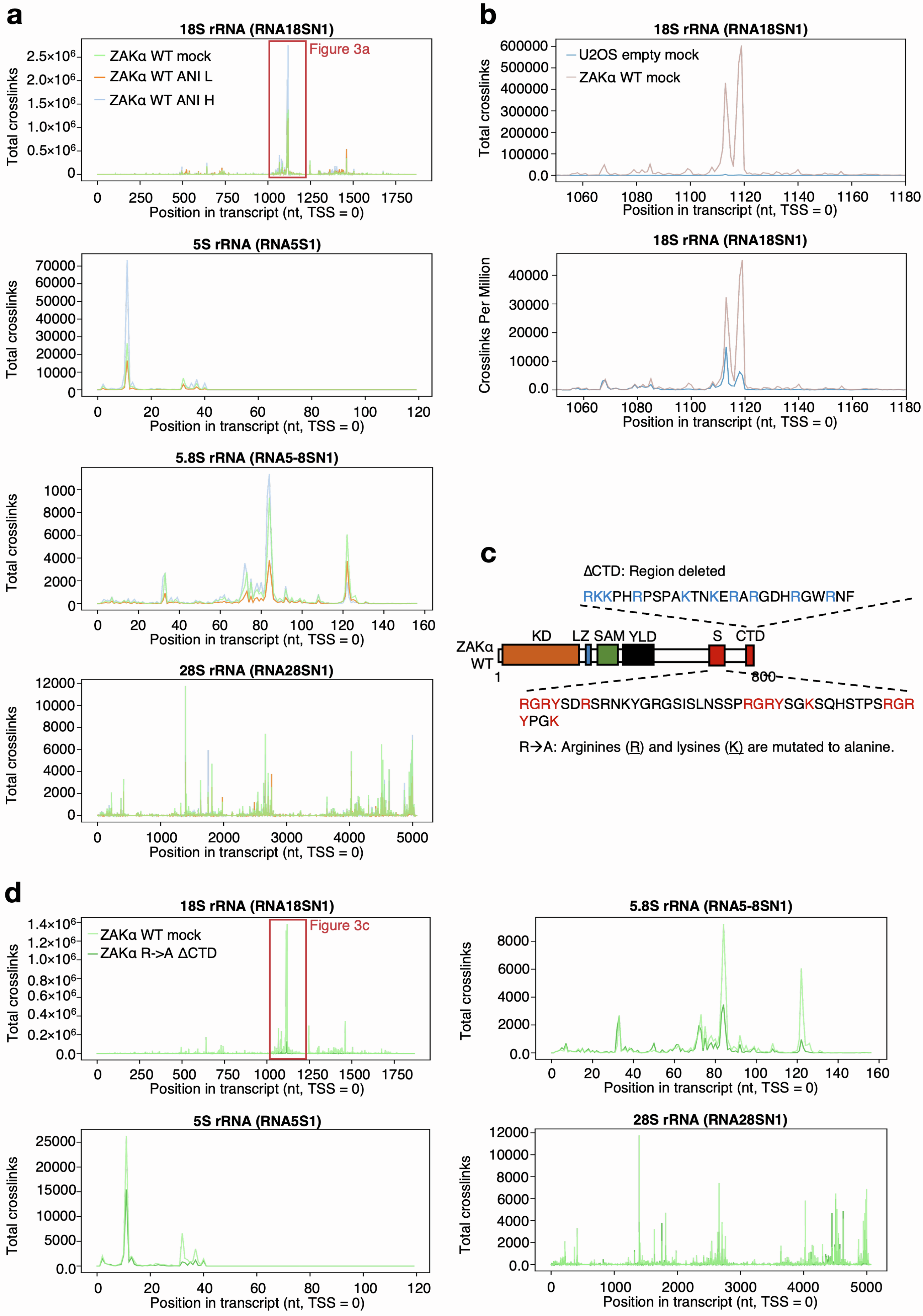
a
18S rRNA (RNA18SN1)
Figure 3a
ZAKα WT mock
ZAKα WT ANI L
ZAKα WT ANI H
Total crosslinks
Position in transcript (nt, TSS = 0)
5S rRNA (RNA5S1)
5.8S rRNA (RNA5-8SN1)
28S rRNA (RNA28SN1)
b
18S rRNA (RNA18SN1)
U2OS empty mock
ZAKα WT mock
Total crosslinks
Crosslinks Per Million
Position in transcript (nt, TSS = 0)
c
ΔCTD: Region deleted
RKKPHRPSPAKTNKERARGDHRGWRNF
KD
LZ
SAM
YLD
S
CTD
ZAKα WT
1
800
RGRYSDRSRNKYGRGSISLNSSPRGRYSGKSQHSTPSRGR
YPGK
R→A: Arginines (R) and lysines (K) are mutated to alanine.
d
18S rRNA (RNA18SN1)
Figure 3c
ZAKα WT mock
ZAKα R->A ΔCTD
5.8S rRNA (RNA5-8SN1)
5S rRNA (RNA5S1)
28S rRNA (RNA28SN1)
Total crosslinks
Position in transcript (nt, TSS = 0)

Figure S3.

iCLIP highlights a single prominent rRNA interaction site for ZAKα, related to Figure 3

a. Total number of sequenced ZAKα crosslinks for individual nucleotides across the four rRNAs. Mock treatment, low anisomycin (ani L) and high anisomycin (ani H) (15 min) conditions have been overlaid. **b.** Total (top) and normalized (bottom) 18S rRNA crosslink counts for WT strep-HA-ZAKα compared to empty U2OS cells subjected to strep purification. **c.** Schematic of ZAKα domain composition. The CTD consists of a span of positively charged amino acids (R, K). The S domain contains three peptide repeats of similar sequence. Mutation of Rs and Ks (red) to As in the S domain combined with deletion of the CTD gives rise to the activation- and ribosome binding-deficient mutant of ZAKα (R->A ΔCTD). KD, kinase domain; LZ, leucine zipper; SAM, sterile alpha-motif; YLD, Yeats-like domain; S, sensor domain; CTD, C-terminal domain. **d.** As in (a), except that crosslinks from WT and R->A ΔCTD ZAKα have been overlaid.

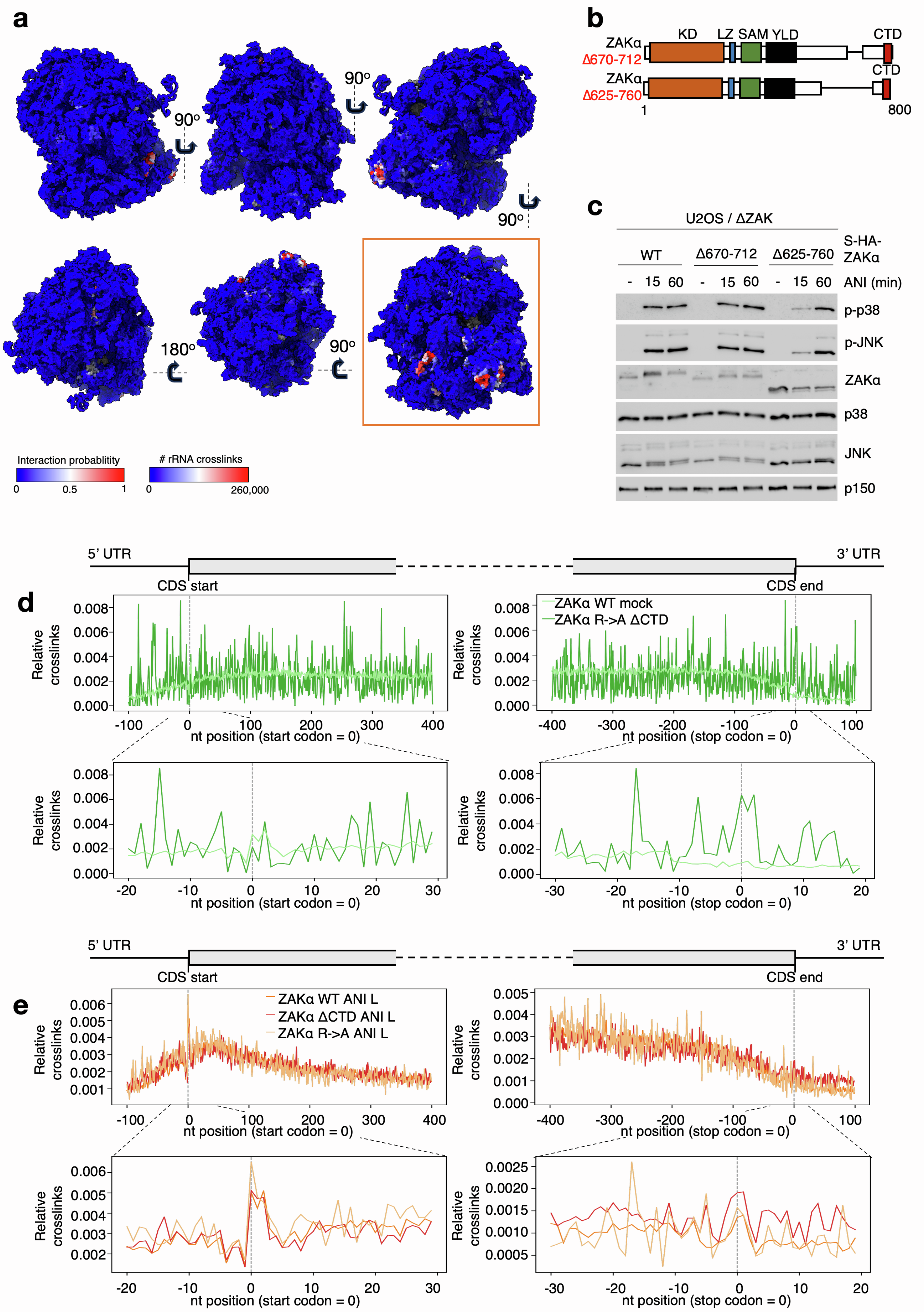
a
90º
90º
90º
180º
90º
Interaction probablility
0
0.5
1
rRNA crosslinks
0
260,000
b
ZAKα
Δ670-712
ZAKα
Δ625-760
KD
LZ
SAM
YLD
CTD
CTD
1
800
c
U2OS / ΔZAK
WT
Δ670-712
Δ625-760
S-HA-ZAKα
- 15 60 - 15 60 - 15 60
ANI (min)
p-p38
p-JNK
ZAKα
p38
JNK
p150
d
5' UTR
CDS start
CDS end
3' UTR
ZAKα WT mock
ZAKα R->A ΔCTD
Relative crosslinks
nt position (start codon = 0)
nt position (stop codon = 0)
e
ZAKα WT ANI L
ZAKα ΔCTD ANI L
ZAKα R->A ANI L

Figure S4.

Crosslinking of ZAKα S and CTD mutants to mRNA, related to Figure 3 and Figure 4

a. Structure of the human ribosome (PDB 4UG0) painted by per-residue ZAKα interaction probabilities and number of ZAKα crosslinks per rRNA residue. Pictures represent the structure from Fig. 3f viewed from all six sides, highlighting two likely ZAKα interaction sites on the ribosome. **b.** Schematic of ZAKα mutants with shortened linkers (Δ670-712 and Δ625-760). **c.** U2OS / ΔZAK cells stably rescued with strep-HA-tagged ZAKα mutants from (b) were treated with anisomycin (ani – 1 μM) for the indicated times. Lysates were analyzed by immunoblotting with the indicated antibodies. **d.** Analysis of normalized mRNA crosslinks for WT and R->A ΔCTD ZAKα around the start (left) and stop codons (right) shown at low (top) and high (bottom) resolution. **e.** As in (d), except that cells expressing WT, R->A and ΔCTD forms of strep-HA-tagged ZAKα were treated with ani (1 μM, 15 min). nt, nucleotide.

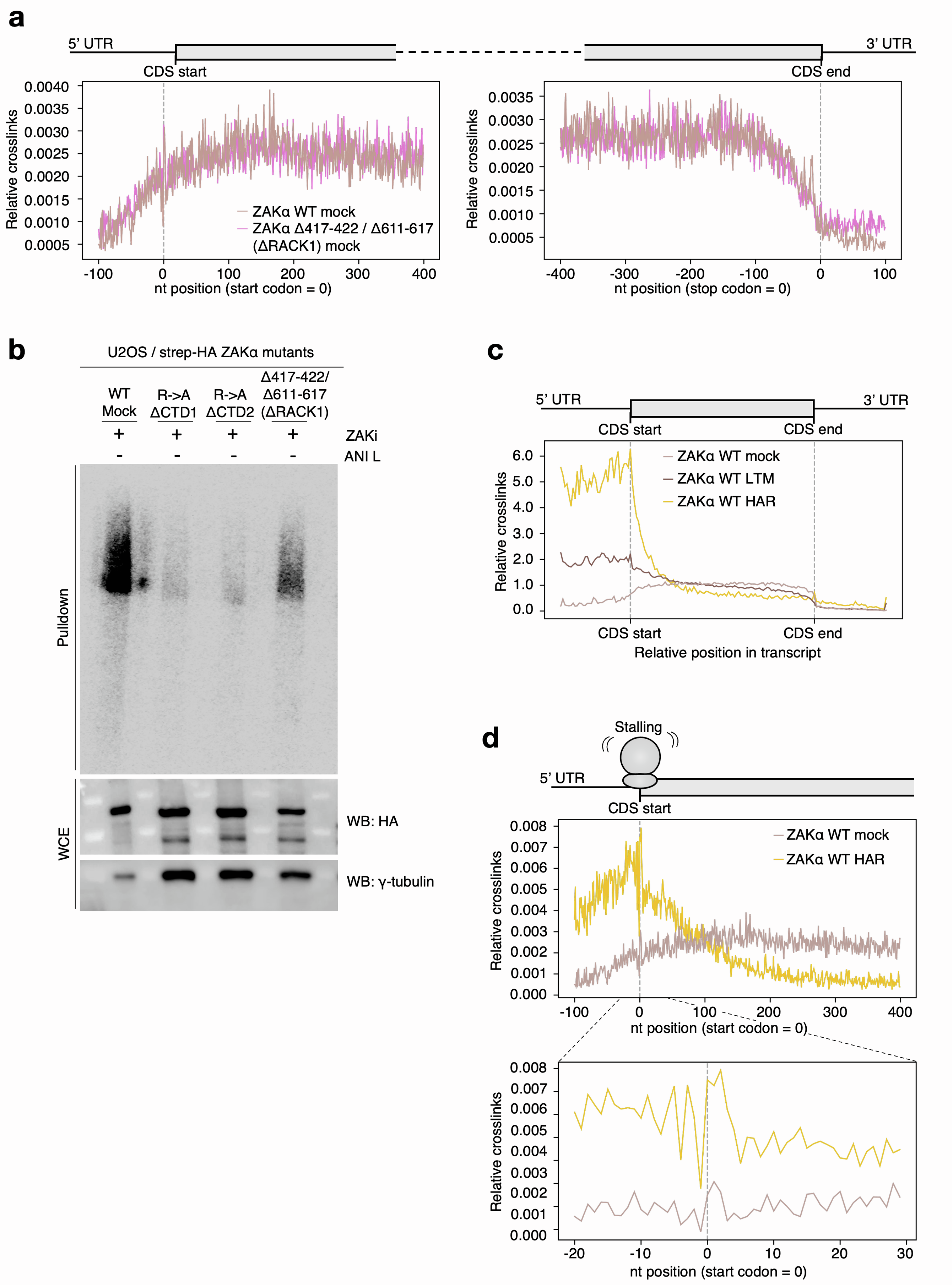
a
5' UTR
3' UTR
CDS start
CDS end
Relative crosslinks
ZAKα WT mock
ZAKα Δ417-422 / Δ611-617 (ΔRACK1) mock
nt position (start codon = 0)
nt position (stop codon = 0)
b
U2OS / strep-HA ZAKα mutants
WT Mock
R->A ΔCTD1
R->A ΔCTD2
Δ417-422/ Δ611-617 (ΔRACK1)
ZAKi
ANI L
Pulldown
WCE
WB: HA
WB: γ-tubulin
c
5' UTR
3' UTR
CDS start
CDS end
Relative crosslinks
ZAKα WT mock
ZAKα WT LTM
ZAKα WT HAR
Relative position in transcript
d
Stalling
5' UTR
CDS start
Relative crosslinks
ZAKα WT mock
ZAKα WT HAR
nt position (start codon = 0)

Figure S5.

mRNA crosslinking of ZAKα relative to position of stalled vs. collided ribosomes, related to Figure 6

a. Analysis of normalized mRNA crosslinks for RACK1 binding-deficient ZAKα (ZAKα Δ417-422 Δ611-617) around the start (left) and stop codons (right). **b.** Comparison of crosslinking efficiency of WT, mRNA binding-deficient (ZAKα R->A ΔCTD) and RACK1 binding-deficient (ZAKα Δ417-422 Δ611-617) mutants of ZAKα. Crosslinked RNA was radiolabelled with ^{32}P. Input materials were analyzed by immunoblotting with the indicated antibodies. **c.** Metagene profiles of total number of crosslinks for mock, LTM-treated (15 μM, 1 h) and HAR-treated (30 μM, 1 h) WT ZAKα along scaled length of spliced mRNAs determined by iCLIP. **d.** Analysis of normalized mRNA crosslinks for ZAKα from cells treated with HAR (30 μM – 1 h) around the start codon shown at low (top) and high (bottom) resolution. nt, nucleotide; LTM, lactimidomycin; HAR, harringtonine.

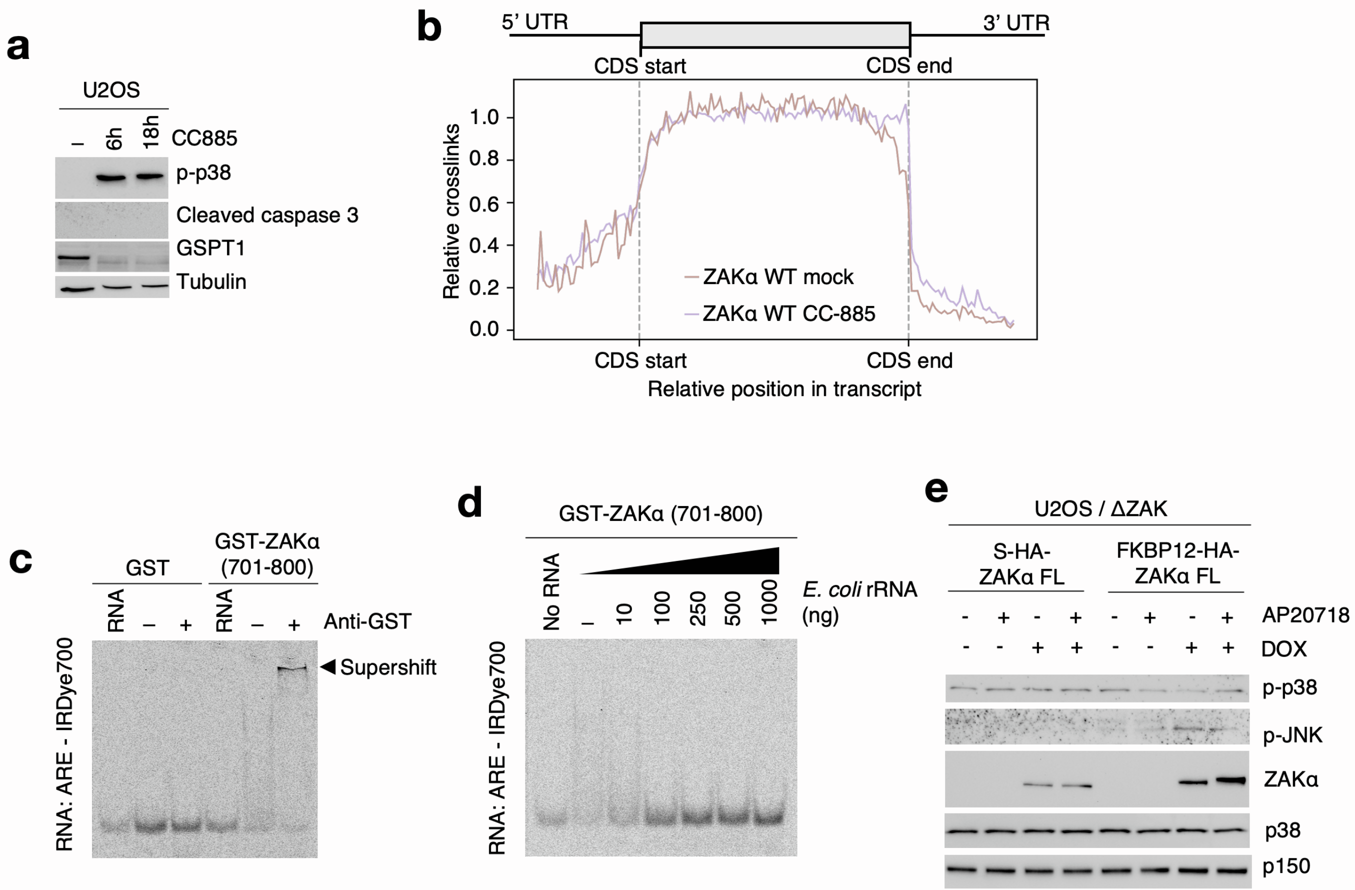
a
U2OS
– 6h 18h CC885
p-p38
Cleaved caspase 3
GSPT1
Tubulin
b
5' UTR
3' UTR
CDS start
CDS end
Relative crosslinks
1.0
0.8
0.6
0.4
0.2
0.0
ZAKα WT mock
ZAKα WT CC-885
CDS start
CDS end
Relative position in transcript
c
GST
GST-ZAKα (701-800)
RNA – + RNA – + Anti-GST
Supershift
RNA: ARE - IRDye700
d
GST-ZAKα (701-800)
No RNA – 10 100 250 500 1000
E. coli rRNA (ng)
RNA: ARE - IRDye700
e
U2OS / ΔZAK
S-HA-ZAKα FL
FKBP12-HA-ZAKα FL
AP20718
DOX
p-p38
p-JNK
ZAKα
p38
p150

Figure S6.

Forced degradation of GSPT1 leads to ribosome stalling and collision at stop codons and activation of ZAKα, related to Figure 6 and Figure 7

a. U2OS cells were treated with the GSPT1/ERF3 degrader CC-885 for 6 and 18 h. Lysates were analyzed by immunoblotting with the indicated antibodies. **b.** Metagene profiles of total number of crosslinks for mock and CC-885-treated (6 h) WT ZAKα along scaled length of spliced mRNAs determined by iCLIP. Notice the accumulation of termination-deficient ribosomes around stop codons. **c.** Recombinant GST or a GST-tagged ZAKα fragment (aa 701-800) was incubated with an AU-rich RNA probe labelled with an infrared dye. GST antibody was added to the indicated reactions (resulting in a supershifted band) and complexes were separated by native gel electrophoresis. **d.** GST-ZAKα-RNA complexes from (c) were incubated with unlabeled *E. coli* rRNA (to compete for binding) in the indicated amounts and analyzed as in (c). **e.** U2OS / ΔZAK cells conditionally expressing FKBP12-HA-ZAKα full-length (FL) from Fig. 7a were treated with doxycycline (DOX - overnight) and AP20187 (50 nM, 1 h) as indicated. Lysates were analyzed by immunoblotting with the indicated antibodies.

Vind et al, Figure S7

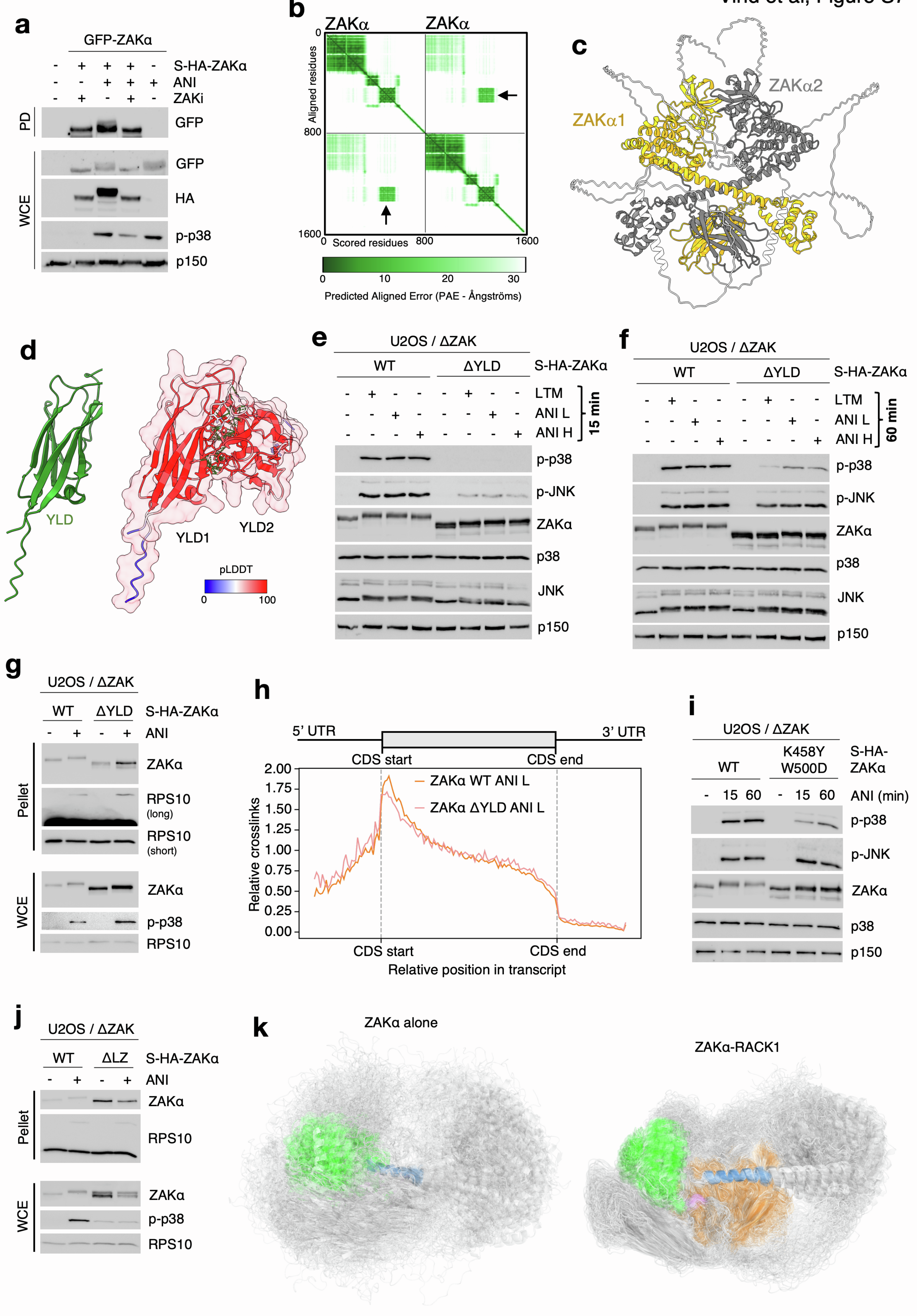

Figure S7.

ZAKα YLD is required for optimal kinase activation, related to Figure 7

a. U2OS cells stably expressing GFP-ZAKα were transfected with Strep-HA-ZAKα and treated with anisomycin (ani - 1 μM) and ZAK inhibitor (ZAKi - 10 μM) for 1 h as indicated. Lysates were subjected to strep purification and pull-down (PD) material and whole cell extract (WCE) were analyzed by immunoblotting with the indicated antibodies. **b.** Predicted aligned error (PAE) matrix plot of AF3-generated prediction of a ZAKα dimer. Values for YLD-YLD binding are indicated with black arrows. **c.** AF3-generated structure from (b). Sequences from start until end of the YLD domain are highlighted by color. **d.** Left: Predicted structure of an isolated ZAKα YLD domain. Right: Predicted structure of a ZAKα YLD dimer. Hydrogen bonds between chains are indicated and chains and interacting side chains are colored according to the predicted local distance difference test (pLDDT) score. **e.** U2OS / ΔZAK cells stably rescued with WT and ΔYLD forms of strep-HA-tagged ZAKα were treated with ribotoxic stress agents ani (L – 0.19 μM; H - 76 μM) or lactimidomycin (LTM – 1 μM) for 15 min and analyzed by immunoblotting with the indicated antibodies. **f.** As in (e), except that cells were treated with ani and LTM for 60 min. **g.** Cells from (e) were treated with ani (1 μM, 1 h) and lysates were ultracentrifuged through sucrose cushions. WCE and pelleted material (pellet) enriched for ribosomes were analyzed by immunoblotting with the indicated antibodies. **h.** Metagene profiles of total number of crosslinks for ani-treated (1 μM, 15 min) ZAKα WT and ΔYLD along scaled length of spliced mRNAs determined by iCLIP. **i.** U2OS / ΔZAK cells stably rescued with WT and YLD point mutated (K458Y W500D) forms of strep-HA-tagged ZAKα were treated with ani (1 μM) for the indicated times. Lysates were analyzed as in (e). **j.** As in (g), except that WT was compared to ΔLZ ZAKα. **k.** Full-length ensembles corresponding to (and colorcoded as in) Fig. 7g.
